# Feasibility and Acceptability of Barbershop-Based HIV Prevention Among Heterosexual Men in Kalangala Islands, Uganda: Protocol for a Cluster Randomized Trial (HPTN 111)

**DOI:** 10.2196/87612

**Published:** 2026-04-17

**Authors:** Caitlin Scoville, Brenda Gati Mirembe, Emily Voldal, Joel Maena, Juliane Etima, Jayla Harris-Wisecarver, Ivan Rukundo, Hassan Ssemere, Doreen Kemigisha, Teopista Nakyanzi, Rita Nakalega, Donaldson F Conserve, Maggie Albano, Molly Dyer, Estelle Piwowar-Manning, Hans Spiegel, Clemensia Nakabiito, Andrew Mujugira, Deborah Donnell, Zubair Lukyamuzi

**Affiliations:** 1 Family Health International 360 Durham, NC United States; 2 Makerere University - Johns Hopkins University Research Collaboration Kampala, Central Region Uganda; 3 Fred Hutch Cancer Center Seattle, WA United States; 4 Milken Institute School of Public Health George Washington University Washington, DC United States; 5 Department of Pathology School of Medicine Johns Hopkins University Baltimore, MD United States; 6 Kelly Government Solutions Rockville, MD United States; 7 Makerere University Infectious Diseases Institute Kampala, Central Region Uganda; 8 Department of Pharmacology and Therapeutics King Ceasar University Kampala Uganda

**Keywords:** barbershop, heterosexual men, HIV prevention, HIV risk reduction counseling, HIV testing, risk factors, sexually transmitted diseases, STI testing, Uganda

## Abstract

**Background:**

Globally, successful strategies to engage high-risk heterosexual men in HIV prevention are scarce, resulting in limited access and uptake. Barbershops offer a potential venue for HIV prevention.

**Objective:**

The primary objective was to evaluate the feasibility and acceptability of a barbershop-based HIV prevention initiative. The secondary objectives were to compare completion of self-initiated HIV testing between intervention and control groups, evaluate the preliminary effectiveness of the intervention on change in behaviors associated with HIV acquisition, compare interest in or use of HIV prevention services between intervention and control groups, and assess interest in long-acting preexposure prophylaxis among all participants and by study arm. The exploratory objective was to evaluate the preliminary effectiveness of the intervention on incident sexually transmitted infections.

**Methods:**

HIV Prevention Trials Network 111 (HPTN 111; Testing a Barbershop-based HIV Prevention Initiative Among Men [TRIM]) is a cluster randomized trial conducted in Kalangala district, Uganda, among barbershop-going men. Approximately 250 men were assigned to either an intervention barbershop (n=12) or a control barbershop to receive the standard of care (n=6). Participants assigned to intervention barbershops received an intervention package that included HIV-status neutral education, distribution of HIV self-test kits, and barber-led peer group sessions. Feasibility and acceptability of the intervention were assessed from participants in the intervention group at week 26 and week 52. The study will also assess the effectiveness of the intervention on changes in HIV testing and use of prevention services. Self-reported sexual behaviors associated with HIV incidence and sexually transmitted infection incidence rates will also be compared to the standard of care.

**Results:**

Data collection began in March 2024 and concluded in June 2025. Participants were followed for 12 months. Data analysis has been completed. The primary manuscript is expected to be submitted for publication by March 30, 2026.

**Conclusions:**

The results of this study will provide crucial information about the feasibility and acceptability of novel interventions, such as barbershops, to impact behavior change, as well as about the engagement of heterosexual men in high HIV transmission settings in HIV prevention and treatment trials in the future.

**Trial Registration:**

ClinicalTrials.gov NCT06148584; https://clinicaltrials.gov/study/NCT06148584

**International Registered Report Identifier (IRRID):**

RR1-10.2196/87612

## Introduction

### Background

The Joint United Nations Program on HIV/AIDS (UNAIDS) and partners are focused on ending the HIV/AIDS epidemic using a variety of strategies [1,2]. However, over the past decade, it has become increasingly clear that the global HIV response is inadequately engaging men [3]. This shortfall has been largely due to a lack of emphasis on acceptable and approachable ways to reach men, which has greatly affected clinical and public health outcomes and targets. Global evidence shows that, when compared to women, there are 1 million more men living with HIV who are unaware of their HIV status and 1.8 million more men not on treatment [1,2]. More AIDS-related deaths have occurred among men than among women over the last decade [4,5].

In 2023, the 95-95-95 strategy for HIV testing and treatment was 83-86-94 among men aged ≥15 years versus 91-91-94 among women counterparts [6]. However, in 2022, a meta-analysis of 129 studies from sub-Saharan Africa (SSA) found that 49% (95% CI 0.41-0.58) of men did not know their HIV status, 58% (95% CI 0.51-0.65) were not on treatment, and 21% (95% CI 0.19-0.23) on treatment were not virally suppressed [7]. The observed difference may be attributable to the meta-analysis incorporating studies conducted between 2014 and 2020, whereas the comparison data are derived from 2023 program reports. Notably, gender disparities in HIV prevention and treatment outcomes have persisted through 2024. Recent estimates indicate that 84%, 87%, and 94% of men know their HIV status, are on treatment, and are virally suppressed, respectively, compared with 92%, 91%, and 95% of women, according to UNAIDS. Suboptimal HIV prevention and care services for men disproportionally increase both their risk of death and HIV transmission [4]. Unfortunately, undiagnosed and untreated HIV among men leads to continued transmission of HIV, especially to adolescent girls and young women, the most susceptible and affected age group for new HIV infections [4,8]. Therefore, diagnosing and treating HIV in men is not only essential for promoting men’s health but also for breaking the cycle of HIV transmission [4,9].

In Eastern and Southern Africa, the HIV epidemic is largely heterosexually transmitted, and heterosexual men are greatly affected [10,11]. However, HIV services increasingly target key populations [12-15], with limited emphasis on heterosexual men [7,16-18]. Fishing communities are especially affected; for example, the Kalangala Islands have an HIV prevalence of 12%, twice the national average of 5.1%, and an HIV incidence of 231 per 100,000 persons [19-21]. Additionally, men make up four times the population of women in the islands, with 48.4% of them unaware of their HIV status [20,22-24].

Compared to women, men and boys are less likely to test for HIV, initiate antiretroviral therapy, and remain engaged in care [4,25,26]. A significant body of research and experience shows a range of complex, multilevel barriers to male engagement in HIV-related services [4,26-28]. These include fear of knowing one’s HIV status and related stigma [29,30], and prevailing cultural, social, and gender norms—such as equating illness with “weakness” and viewing clinical settings as “female” spaces [31,32]. Men in Africa often assume their status matches their partner’s [33] and worry about loss of income from taking time off work [30,34-37]. Health systems also create structural barriers by focusing on reproductive-age women who can access care through antenatal care, and maternal and child health programs—entry points that do not exist for men [4]. Limited clinic hours and facility-based service models further exclude men, especially those who work [4,26].

HIV testing is the gateway to prevention and care. The World Health Organization recommends HIV self-testing (HIVST) as an additional HIV testing approach, and it is feasible and acceptable among various populations, including heterosexual men [38-40]. Over 4.8 million test kits have been distributed in SSA, including Uganda [41]. However, a recent systematic review found that, despite high uptake, many questions remain about HIVST’s effectiveness in reaching historically underserved men and ensuring linkage to prevention and care [42].

Community-based HIV testing services (CHTS), such as mobile and home testing [4], show high potential for reaching men [4,38]. Men prefer CHTS over facility-based testing [26,38-40,43,44] due to its convenience and lower stigma [40,44-48]. Specifically, CHTS improves testing among low- and middle-income men, young men, and those who would otherwise avoid health facilities [40,44-48]. Still, broader reach and linkage to care mechanisms are lacking [38].

Peer intervention approaches, which use individuals with similar demographic and social backgrounds to the target population, have proven effective in promoting behavior change, sustaining reductions in HIV risk behaviors [49,50]. Peer-led education enhances knowledge exchange, reduces stigma, builds trust, and supports uptake of services such as HIVST [40,44,51-53]. Notably, community-based peer support has improved HIV service uptake and adherence in several settings [54,55].

Despite this progress, affordable and scalable combination programs tailored for heterosexual men in high-burden settings remain scarce [56]. Thus, innovative community-based strategies are urgently needed to improve men’s HIV testing and linkage to prevention and care services. Barbershops offer informal, easily accessible (ie, convenient location and hours of operation), and collegial spaces where men often feel safe to talk about personal health issues, and the majority use the same barber most of the time [57]. Studies in the United States on barbershop-based health promotion interventions for Black men showed that barbershop-based interventions achieved satisfactory recruitment and retention of men in health services [58]. Another study in the United States found barbershop business venues appropriate for HIVST promotion and distribution [59].

Therefore, the purpose of HIV Prevention Trials Network (HPTN) 111, also known as TRIM (Testing a Barbershop-based HIV Prevention Initiative Among Men), was to develop and assess the feasibility, acceptability, and preliminary effectiveness of a barbershop-based HIV prevention initiative among heterosexual men in a high–HIV-prevalence setting. The study leverages trusted relationships between men and their barbers to deliver a prevention package consisting of HIV education, HIVST distribution, and barber-led peer group sessions. This protocol responds to a critical need to develop focused interventions to engage men in HIV prevention and care to bring male HIV prevention and care outcomes on par with those of women.

### Objectives

The primary objective was to evaluate the feasibility and acceptability of a barbershop-based HIV prevention initiative. The secondary objectives were to compare completion of self-initiated HIV testing between intervention and control groups, evaluate the preliminary effectiveness of the intervention on change in behaviors associated with HIV acquisition, compare interest in or use of HIV prevention services between intervention and control groups, and assess interest in long-acting preexposure prophylaxis (PrEP) among all participants and by study arm. The exploratory objective was to evaluate the preliminary effectiveness of the intervention on incident sexually transmitted infections (STIs).

## Methods

### Overview

**Figure 1:**
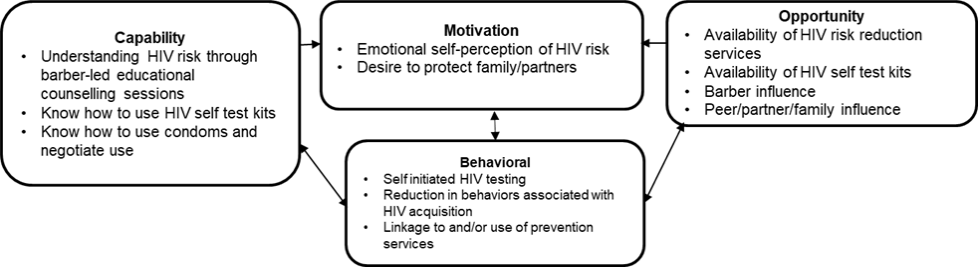
The Capability, Opportunity, and Motivation Model of Behavior Change used to motivate and design the barbershop-based HIV prevention intervention in the HIV Prevention Trials Network 111 trial.

HPTN 111 (TRIM) was a pilot study conducted through the HPTN. HPTN 111 is funded by the National Institute of Allergy and Infectious Diseases (NIAID), under the National Institutes of Health. NIAID provides technical assistance and coordination of the study, including regulatory reviews and study conduct oversight. HPTN 111 is registered on ClinicalTrials.gov (NCT06148584). The study is based on the capability, opportunity, or motivational components of the Capability, Opportunity, and Motivation Model of Behavior Change (COM-B; Figure 1) [60]. The COM-B postulates that changing the behavior of an individual, a group, or a population requires changing either their capability, opportunity, and/or motivation needed to perform the behavior [61]. Therefore, in this study, we used the COM-B to provide a barbershop-based HIV prevention intervention to men living in a high heterosexual HIV incidence setting and who could benefit from HIV prevention services. The initiative aimed to increase men’s capability, opportunity, and motivation through the provision of HIV education and prevention measures directly from their barbers and based at their barbershops. This study will provide valuable information about the feasibility and acceptability of a barbershop-based HIV prevention initiative and the potential for scale-up in other settings where barber-client relationships are an important component of men’s social structure. Lastly, we followed the SPIRIT (Standardized Protocol Items: Recommendations for Interventional Trials) reporting guidelines during the drafting of this protocol paper.

### Study Design

**Figure 2:**
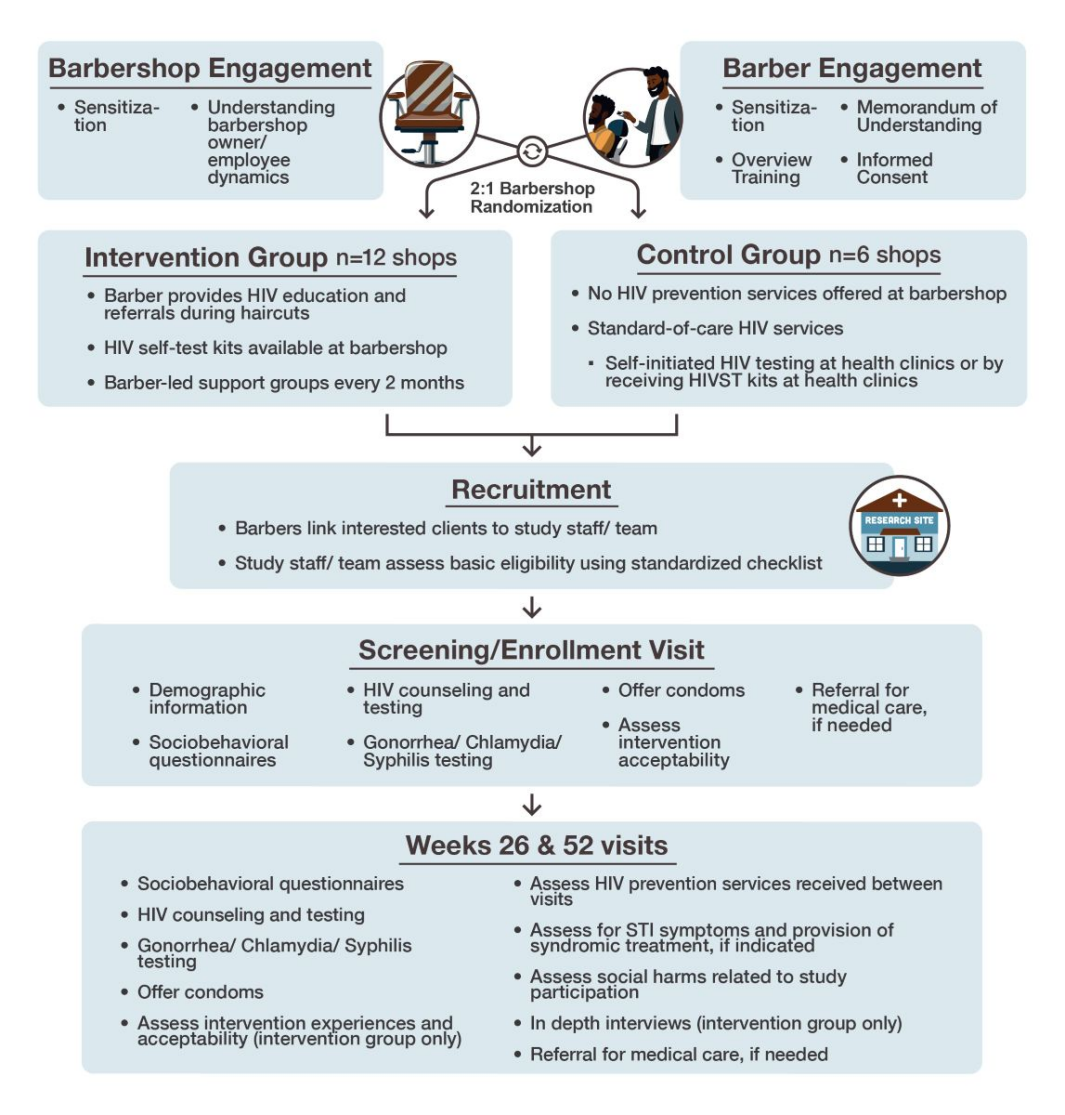
Overview schema of the HIV Prevention Trials Network 111 study design conducted in barbershops in the Kalangala Islands. HIVST: HIV self-testing; STI: sexually transmitted infection.

HPTN 111 (TRIM) is a cluster randomized trial testing the feasibility and acceptability of a barbershop-based HIV prevention intervention among heterosexual men who have certain risk factors for HIV acquisition. The study was conducted in Kalangala district, Uganda, among barbershop-going men. Barbershops acted as clusters, and 18 purposively selected barbershops were randomized in a 2:1 ratio to the intervention or control group (Figure 2).

Men enrolled from the intervention barbershops received a barbershop-based HIV prevention intervention from their barber, while those enrolled in control barbershops received standard-of-care (SoC) HIV services. At intervention barbershops, trained barbers provided HIV status-neutral education, distributed HIVST kits at the barbershop, and led peer group sessions. The SoC services included existing HIV prevention services—namely, HIV testing and related services from nearby health facilities. All enrolled participants were followed for 12 months with biannual study visits at a designated study clinic. Intervention barbers also had quarterly follow-up visits to assess social impacts, acceptability, and feasibility of the intervention, and interviews to help better understand their experience within the intervention. Study objectives and corresponding end points are provided in Table 1.

**Table 1:** HIV Prevention Trials Network 111 study objectives and end points to be evaluated among 18 barbershops randomized 2:1 to implement the barbershop-based HIV prevention services package or receive standard of care.

Objective and topic				End point assessment		Population
**Primary**						
	**Evaluate the feasibility and acceptability of a barbershop-based HIV prevention initiative**					
		Participant survey responses about feasibility/acceptability	Questions about how participants feel about the proposed intervention before receiving it Questions about acceptability after receiving the intervention		Both groups Intervention group	
		Barber survey responses about feasibility/acceptability	Questions about how barbers feel about the proposed intervention before delivering it Questions about the feasibility/acceptability of talking to clients about the study Questions about acceptability after delivering the intervention		Both groups Both groups Intervention group	
		Recruitment and retention	Participant recruitment in the study Participant visit completion Barber visit completion		Both groups Both groups Intervention group	
		Barber-participant interactions and intervention delivery	Participant-reported frequency of interactions with the barber and interventions received Barber-reported frequency of intervention delivery and interventions delivered Frequency and content of group sessions as observed by study staff		Both groups Intervention group Intervention group	
**Secondary**						
	**Compare the completion of self-initiated HIV testing between intervention and control groups**					
		Self-initiated HIV testing by participants	Whether participants test for HIV between visits		Both groups	
	**Evaluate the preliminary effectiveness of the intervention on the change in behaviors associated with HIV acquisition**					
		Self-reported participant risk behaviors	Participant survey responses about risk behaviors, such as condom use and the number of sexual partners		Both groups	
	**Compare interest in or use of HIV prevention services between intervention and control groups**					
		Self-reported participant use of HIV prevention services	Uptake of VMMC^a^ PEP^b^ access and use PrEP^c^ access and use		Both groups Both groups Both groups	
	**Assess interest in long-acting PrEP among all participants and by study arm**					
		Self-reported participant survey on long-acting PrEP	Interest in the use of long-acting injectable PrEP		Both groups	
**Exploratory**						
	**Evaluate the preliminary effectiveness of the intervention on incident STIs**					
		Diagnostic testing results	Incidence of HIV Incidence of gonorrhea and chlamydia		Both groups Both groups	

^a^VMMC: voluntary medical male circumcision.

^b^PEP: postexposure prophylaxis.

^c^PrEP: preexposure prophylaxis.

### Sample Size

HPTN 111 (TRIM) is a pilot study with a primary objective of assessing feasibility and acceptability, measured in the intervention group only (12 barbershops). The sample size was determined based on the following two key considerations: (1) logistical and implementation feasibility [62] and (2) sufficient power to detect differences in a key secondary outcome of HIV testing uptake. A total of 18 barbershops were to be strategically selected, reflecting the number that could be feasibly supported by the study team and within the available budget, as guided in feasibility studies [63]. Selection would be guided by the predefined eligibility criteria, with additional consideration for logistical efficiency. Specifically, barbershops should not be located excessively far from one another, yet should also avoid being in very close proximity. A minimum separation radius of approximately 5 km between sites would be considered to prevent contamination between intervention and control barbershops. While logistical feasibility was the primary driver, to inform client recruitment targets, we also assessed the number of participants per shop needed to detect meaningful differences in HIV testing between study arms, defined as a difference between arms of 20% in the proportion of participants self-initiating HIV testing [63]. A 2:1 randomization was applied, which both increased the number of intervention shops contributing data on feasibility and acceptability and allowed comparisons between arms on secondary outcomes. Enrollment targets of at least 10 participants per each of the 18 barbershops were based on the key considerations above, as well as maintaining at least 80% power for the HIV testing analyses under a variety of possible scenarios (Multimedia Appendix 1). Therefore, each shop would recruit at least 10 participants to reduce the variability in sample size between barbershops, and the protocol team would be informed that it is optimal for power if the 250 participants are evenly distributed across the 18 barbershops, although the variability in the barbershop and village sizes might prevent this.

### Community Engagement, Barbershop Selection, and Barber Training

The study team obtained administrative approval and permission from local leaders to engage various communities and stakeholders in the study area (Kalangala district). This process involved barbershop assessment and selection, district and community stakeholder engagement, and barber training. The study team conducted stakeholder engagement meetings organized with the support of district and local leaders. Stakeholders included local and religious leaders, community representatives from fishing communities, boda-boda cyclists, palm plantation workers, nongovernmental organizations, and village health teams. Meetings were used to discuss perceptions, attitudes, and challenges surrounding the barbershop-based HIV prevention initiative and possible ongoing mitigation strategies.

Participating barbershops were purposively selected on Bugala Island (the largest island in Kalangala District) based on set criteria (Textbox 1) assessed by the study team. The barbershops were largely selected from busy trading centers/towns and landing sites. The barbers participating in each shop were also required to meet set criteria listed in Textbox 1.

**HIV Prevention Trials Network (HPTN) 111 eligibility criteria for the barbershops, barbers, and participants recruited in the Kalangala Islands.**

**Barbershop inclusion criteria**

- Adequate space to deliver the intervention to clients
- Existence as a shop for at least 6 months
- A customer base of at least 15 clients per week
- A minimum radius of 5 km between other participating shops

**Barber inclusion criteria**

- At least a primary level of education
- Willing and able to participate in the study, recruit participants, and deliver the intervention if their shop is randomized to the intervention group

**Participant**

- Inclusion criteria
  At least 16 years of age
  Any participants 16-17 years of age must meet the definition of a mature or emancipated minor per the Uganda National Council for Science and Technology guidelines.
  Identifies as a heterosexual male
  Able and willing to provide informed consent
  Has certain risk factors for HIV acquisition, based on self-report of at least one of the following in the last 3 months: (1) had condomless sex with a person of unknown HIV status or a person living with HIV; (2) had more than one sexual partner
  HIV negative, per the Ugandan Ministry of Health guidelines and the HPTN 111 Study Specific Procedural Manual
  A regular customer at a participating barbershop, defined as visiting a specific participating barbershop for barber services at least three times prior study participation
- Exclusion criteria
  Not planning to stay in the study catchment area in the next 12 months
  Any other condition that, in the opinion of the Investigator of Record, would preclude informed consent, make study participation unsafe, complicate interpretation of study outcome data, or otherwise interfere with achieving the study objectives

On completion of the barbershop assessment and selection, barbers from the 18 selected barbershops were further engaged and trained through consultative and participatory approaches. They were engaged to assess commitment and trained on the study overview, general HIV education, communication skills, and study recruitment requirements. The study team used didactic presentations, illustrations, group discussions, and role plays for these trainings.

Barbers from barbershops assigned to the intervention group received additional training on the implementation of the intervention. Pre- and posttraining assessments were used to ensure that barbers had sufficient knowledge about HIV and the intervention before participant recruitment. Training was ongoing during the study, with study staff providing support to barbers and assessing knowledge and confidence in participant recruitment and intervention delivery. Refresher training and cross-sharing with other barbers were completed if knowledge gaps were identified.

### Randomization

The randomization procedures assigned the 18 selected barbershops to either the intervention or control arm in a 2:1 ratio. Due to the relatively small number of units being randomized (n=18), covariate-constrained randomization was used to balance 3 barbershop characteristics between treatment groups: the estimated population of the village or town where the barbershop is located, the average number of customers a barber served per day (as reported by the lead barber), and the estimated travel distance between the barbershop and the nearest health facility (in km). The restrictions would ensure that the difference between groups is within 300 for the average village population, within 4 for the average number of clients per day, and within 0.75 km for the average distance to the nearest health facility.

Randomization was done in 2 stages. In the first stage, the shops were allocated into 3 groups of 6 shops each using covariate-constraint randomization described above. In the second stage, a public randomization event was conducted, and 2 groups (12 shops total) were assigned to the treatment group, and 1 group (6 shops) was assigned to the control group. The first stage of randomization was executed by statisticians at Fred Hutchinson Cancer Center; the second stage involved barbers publicly drawing opaque balls from a transparent bucket to randomly determine treatment group assignment, ensuring transparency and fostering community engagement.

Due to the nature of the intervention, neither barbers nor study participants were blinded. Barbers were asked not to disclose their treatment assignment during recruitment activities; however, because of the public randomization process and visible delivery of the intervention to other clients, clients would become aware of their barbershop’s treatment assignment prior to enrollment.

### Ethical Considerations

The HPTN Ethics Working Group developed the HPTN Ethics Guidance for Research, a network-wide ethical principles document, which guided the HPTN 111 (TRIM) study conduct.

The protocol and the informed consent forms (ICFs) were reviewed and approved by the HPTN Scientific Review Committee with respect to scientific content and compliance with applicable research and human participants’ regulations.

The protocol, site-specific ICFs, participant education and recruitment materials, other requested documents, and any subsequent modifications were reviewed and approved by the Joint Clinical Research Center (JCRC 2023-54) and the Johns Hopkins Medicine (IRB00420132) and cleared by the Uganda National Council for Science and Technology (HS3430ES), which are responsible for oversight of research conducted at the study site.

After initial review and approval, the responsible institutional review boards (IRBs)/ethics committees will review the protocol at least annually. The investigator will make safety and progress reports to the IRBs/ethics committees at least annually and provide a study termination or completion report within 3 months after the study is completed. The annual reports include the total number of participants enrolled in the study, the number of participants who completed the study, all changes in the research activity, and all unanticipated problems involving risks to participants or others. The study site submits documentation of continuing review to the Division of AIDS (DAIDS) Protocol Registration Office, in accordance with the current DAIDS Protocol Registration Policy and Procedures Manual.

All participants provided informed consent prior to their involvement in any study procedure (Multimedia Appendix 2). We ensured participant privacy and confidentiality by using anonymized and deidentified data. Participant data, including ICFs, are kept under lock and key and only accessed by the research team or authorized personnel. Participants were reimbursed and compensated for the time spent at the study site at the rate approved by the IRB.

### Study Procedures

#### Recruitment, Screening, and Enrollment

Participating barbers were responsible for recruiting their male clients into the study. Barbers were provided with posters and information brochures to help with recruitment. All barbers were asked to complete a simple, anonymous log of the number of men contacted and linked to the study for screening. Men who were interested in the study following barber-led recruitment were referred directly to study staff who would complete a prescreening assessment. During the prescreening, potential participants were assessed for sexual behaviors associated with HIV acquisition, their residence, barbershop attendance status, and age. Potential participants who remained eligible following this prescreening assessment would proceed to the central study clinic for further eligibility assessment and enrollment. Study procedures are shown in Figure 2.

At the study clinic, potential participants completed a full screening visit, including providing written informed consent, collection of basic demographic and sociobehavioral information, and blood collection for HIV and syphilis testing. Participants who remained eligible after screening procedures would immediately proceed to enrollment or return for enrollment within 14 days. Eligibility criteria for participants are provided in Textbox 1. Ineligible individuals would be referred to appropriate medical care, including HIV prevention services or HIV care, based on their needs. Participants could be rescreened up to 2 times if found to be ineligible at the initial screening attempt. Participants who would have a reactive HIV test at screening would not be able to rescreen.

Individuals were considered enrolled in the study once study staff verified that all eligibility criteria were met. Subsequent enrollment procedures included gonorrhea and chlamydia testing, administration of additional sociobehavioral questionnaires via audio computer–assisted self-interview (ACASI), assessment of the acceptability of the barbershop-based intervention, and counseling on the recommended quarterly HIV testing schedule. This recommendation aligned with the Uganda Ministry of Health National Implementation for HIV Counselling and Testing Services, which advises that individuals with certain HIV acquisition risk factors undergo HIV testing every 3 months [64]. Participants were encouraged to contact the study team between visits to share results of any HIV tests they would have completed.

Enrolled participants were informed of the study group to which their barbershop was randomized, so that they were aware of whether they would receive the intervention from their barber. For participants from an intervention shop, the respective barber was notified to ensure that the participant received the barbershop-based intervention during their next regular visit to the barbershop.

#### Follow-Up Study Visits

Following enrollment, follow-up study visits occurred at 26 weeks and 52 weeks post enrollment. HIV testing and counseling, as well as gonorrhea and chlamydia testing, were conducted at both visits. A symptom-directed physical exam was completed, and any STI symptoms were treated as per the SoC syndromic management. Participants were asked to provide information on their HIV testing behaviors between visits, use of other HIV prevention services (eg, HIV PrEP, postexposure prophylaxis, voluntary medical male circumcision), sexual behavior (via ACASI), and social harms experienced due to study participation. All participants were asked about their experiences of receiving barbershop-based HIV information to assess fidelity in the intervention group and cross-contamination in the control shops.

Interim study visits were completed upon participant request or as deemed necessary by the study team. For example, interim visits would occur if a participant contacted study staff with HIV test results, either requiring follow-up testing or referral to care, or if they received a positive STI test result and required treatment.

Participants either received the barbershop-based HIV prevention initiative (intervention group) or SoC HIV prevention services (control group) based on the barbershop from which they were recruited. Participants were expected to continue visiting their respective barbershops for regular services, but these barbershop visits were not considered formal study visits.

#### Qualitative Interviews

Participants in the intervention group were further asked about the acceptability of receiving the intervention from their barber. A subset of approximately 25 participants from the intervention group were purposively selected to participate in individual in-depth interviews at week 26 and/or week 52. Additional interviews were conducted if saturation was not reached—that is, the point at which no new themes or insights emerge, ensuring a comprehensive understanding of participants’ experience within the study—barbers and clients alike. The interviews explored the acceptability of the barbershop-based HIV prevention initiative and use of HIV prevention strategies to gain deeper insight into how the intervention may influence behavioral change (Multimedia Appendix 3).

Sample size was guided by the principle of informational adequacy and thematic saturation [65]. Studies examining relatively homogeneous populations with narrowly defined research objectives have demonstrated that saturation of major themes often occurs within 20-30 interviews [66]. The study population in this protocol, heterosexual men attending selected barbershops in the Kalangala Islands, represents a geographically and socially bounded group exposed to a standardized intervention.

Purposive sampling further strengthens informational yield. Participants were selected based on characteristics such as age, HIV risk behaviors, use of prevention services, experiences during the study, and barbershop attended. This approach ensures variation within key implementation domains while maintaining contextual coherence. A sample of approximately 25 interviews is therefore methodologically appropriate to generate an in-depth understanding of acceptability, behavioral responses, and contextual influences within a feasibility trial framework.

### Intervention Group

Participants in the intervention group received the HIV prevention intervention from their barber. The intervention includes (1) status-neutral HIV education and referral information for HIV services, (2) distribution of HIVST kits through the barbershops, and (3) barber-led peer group sessions.

#### Status-Neutral HIV Education and Referral Information for HIV Services

During the routine barber-participant interaction, barbers would provide general, status-neutral HIV education while providing haircut services. A counseling guide was provided to ensure that barbers follow standardized messaging during participant education. If other clients were present while an enrolled participant was receiving a haircut and study-related services, the barbers were trained to extend the education to everyone in the shop at that time. The provided HIV education and counseling was neutral and general information as guided by the Ugandan Ministry of Health manuals for HIV education in public spaces and, therefore, approved by the IRB to share in spaces with nonparticipants. Participants were educated on the risks of HIV acquisition, strategies to reduce those risks, the recommended HIV testing schedule, and where to find other HIV prevention services. Barbers would also share knowledge and information about existing HIV prevention services and resources in Kalangala district, including comprehensive HIV education and counseling, PrEP, postexposure prophylaxis, and voluntary medical male circumcision services.

#### Distribution of HIVST Kits Through the Barbershops

Barbers maintained a stock of study-provided HIVST kits in the shop and offered them to participants to take home and use at their convenience. Participants would take as many test kits as needed, including for use with their partners. Barbers would educate participants on how to properly complete the test and inform them of the recommended testing frequency (ie, every 3 months for individuals with certain risk factors for HIV). HIVST kits were packed in opaque bags to allow participants to carry them discreetly out of the shop. If a nonstudy client was present during HIV education and expressed interest in taking an HIVST kit, they would be permitted to receive one.

#### Barber-Led Peer-Group Sessions

Each barber led his study clients in a peer group session every 2 months to provide additional HIV education and facilitate discussion. These sessions would foster peer support and the sharing of experiences among participants. Study staff assisted with coordinating the peer groups, recording attendance, documenting the HIV education topics covered, and assessing the quality and accuracy of the information delivered.

**Figure 3:**
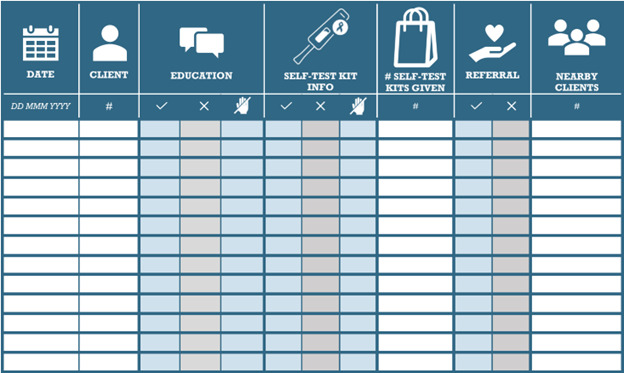
Template for tracking barber delivery of the intervention to their male clients, used by barbers in the intervention arm during participating clients’ haircuts.

The barbers documented the provision of the intervention to their enrolled clients using a study intervention tracking document (Figure 3). They were trained on best practices for documentation, and study staff would provide real-time refresher training as needed.

Study staff conducted approximately weekly check-ins with barbershops to review processes, troubleshoot issues, address concerns, and encourage barbers to deliver the intervention to study clients during routine barbering services (see Multimedia Appendix 4 for participant schedule of events).

### Control Group

Participants in the control group would not receive any HIV prevention services from their barbershop or barber. During study visits at the clinic, they received counseling on routine HIV services, including HIV testing, HIVST kits, and other HIV prevention services available at nearby health facilities. Barbers in the control group were not notified about participants enrolled from their shops and were withdrawn from the study upon completion of the enrollment phase (see Multimedia Appendix 4 for participant schedule of events).

### Retention

The study aimed to achieve participant retention of 90% at week 26 and week 52 visits. Strategies such as the collection of full locator information for participants, flexible study clinic hours, and the option for off-site visits would be used. However, to prevent undue influence in HIV prevention-seeking behaviors, the study team limited contact with participants between study visits.

### Barber Study Procedures

Barbers in this study had a unique role as recruiters, implementers of the intervention, and study participants. Therefore, barbers participating in HPTN 111 (TRIM) carried out a series of administrative and study-specific activities. Barbers/barbershop owners and the study team signed a memorandum of understanding to clearly outline expectations of both parties before the commencement of study activities. All participating barbers also completed written informed consent to ensure they fully understood and voluntarily agreed to participate in the research procedures required throughout the study.

Initially, study staff collected basic information about each participating shop, such as location, total number of clients, hours of operation, cost of services, etc. At baseline, barbers provided demographic information and completed a questionnaire on the acceptability and feasibility of the intervention. At the end of the recruitment period, barbers were asked to complete brief questionnaires about their recruitment experiences. Barbers in the intervention group remained in the study and completed brief questionnaires on acceptability, feasibility, and social impacts of delivering the intervention approximately quarterly during the study follow-up. Intervention barbers also participated in individual in-depth interviews at approximately 6 and 12 months after starting intervention delivery to further understand their views on feasibility, acceptability, facilitators and barriers, and overall experiences of delivering the intervention (see Multimedia Appendix 5 for barber-specific schedule of events and Multimedia Appendix 6 for barber in-depth interview guide). Intervention barbers completed a simple checklist (Figure 3) of the services provided to each client enrolled in the study each time the participant visited the barbershop. The checklist did not contain any direct identifiers of the clients and was stored in a lockable cabinet at the shop, to be collected weekly by study staff*.*

### Data Management

HPTN 111 (TRIM) data were managed in REDCap (Research Electronic Data Capture; Vanderbilt University) Cloud, a platform used by the HPTN Statistical and Data Management Center to receive and manage study data. The study site completed electronic case report forms (CRFs) by entering data into the REDCap Cloud study database. Data would be entered directly into the study database, collected first on paper CRFs and then entered into the study database, entered directly by study participants (ACASI responses only), and/or entered into the study database based on other non-CRF source documents (eg, laboratory reports, testing logs, and chart notes).

### Data Analysis

The primary objective of HPTN 111 (TRIM) is to assess the feasibility and acceptability of a barbershop-based HIV prevention intervention. To achieve this, a variety of end points will be assessed (Table 1).

To assess feasibility and acceptability of the intervention, the following were assessed from participants in the intervention group: participant responses from surveys at week 26 and week 52 regarding acceptability of the intervention, barber responses from surveys assessing feasibility and acceptability of delivering the intervention, recruitment rates during enrollment and participant retention in the study at week 26 and week 52, and the frequency of barber-participant interactions (Multimedia Appendix 7). These outcomes were prespecified and will be summarized and described, but specific progression criteria or targets for these outcomes were not prespecified, which is a limitation driven by the novelty of this intervention. The study also assessed the effectiveness of the intervention on changes in HIV testing and use of prevention services. Self-reported sexual behaviors associated with HIV incidence and STI incidence rates will also be compared to SoC.

For end points involving comparisons between groups, as well as those assessed only within the intervention group, analyses will follow intention-to-treat principles. Formal comparisons will account for the clustering by aggregating data at the barbershop level and adjusting for the covariates constrained by randomization [67]. No interim analyses are planned. Secondary end points (Table 1) will be assessed using the same statistical methods. Because feasibility and acceptability outcomes overlap with critical information about study conduct, certain data, such as recruitment and enrollment summaries and information on the frequency and quality of intervention delivery by the barber—would be shared with investigators and study staff. Summaries of other end points not related to study conduct (eg, self-initiated HIV testing) would remain unavailable to investigators and study staff for the duration of the trial. Data and safety monitoring procedures for the trial will involve summaries of reported outcomes and social impacts. Reporting of end points not related to study conduct would be available only to members of the study monitoring committee (SMC) for this study and will be withheld from study investigators and staff members.

Qualitative interviews were digitally recorded, transcribed, translated where necessary, and analyzed systematically. A hybrid thematic analysis approach, combining deductive and inductive coding strategies [68], was used to analyze the data. The intervention itself is guided by COM-B [69]. Therefore, our analytic framework incorporated both deductive coding informed by COM-B constructs and inductive coding.

Emergent themes not predetermined by the framework, such as trust in barbers, social cohesion within fishing communities, mobility between islands, or unintended social impacts, were identified through open coding. Thematic analysis proceeded through the following stages [70]: (1) review of interview debrief reports, (2) familiarization with transcripts, (3) generation of initial codes, (4) development and refinement of a codebook, (5) double coding of selected transcripts to enhance analytic rigor and for determination of interrater reliability among the coders, (6) identification and refinement of themes, and (7) interpretation of themes in relation to feasibility, acceptability, and preliminary effectiveness.

This hybrid approach allowed theoretical alignment with the COM-B while ensuring contextual sensitivity to the lived experiences of participants in Kalangala. Such integration is consistent with implementation science methodologies that combine theory-driven and data-driven analysis [71].

Determination of saturation included descriptive saturation, interpretive saturation, and team-based review. Descriptive saturation was assessed based on no new themes emerging from successive interviews and would be implemented by team-based reviews of interview debrief reports, which were discussed at weekly team meetings. Similarly, at the weekly team-based meetings, which included the protocol team and the interviewers, interpretive (meaning) saturation was assessed. Meaning saturation occurs when themes are fully elaborated, and additional interviews do not provide new depth, nuance, or variation [72]. The team used the interview debrief reports to document thematic development, review variation across purposively selected subgroups (eg, age groups, different barbershops, and levels of HIV prevention uptake), and determine whether thematic explanations sufficiently captured contextual complexity. This guided the team in determining that saturation had been reached.

### Data Safety and Monitoring

An SMC conducted interim reviews of study progress, including participant accrual, retention, social harms, and quality of data collection. Reviews occurred approximately every 6 months. The SMC provided recommendations on study conduct to ensure that the study can meet the primary objectives.

Due to the minimal-risk nature of the study and absence of a biomedical intervention, there was no centralized reporting of adverse events. However, the study site would report all adverse events and social harms according to local IRB and regulatory requirements.

## Results

Data collection began in March 2024 and concluded in June 2025. Participants were followed for 12 months. Data analysis has been completed. The primary manuscript is expected to be submitted for publication by March 30, 2026.

## Discussion

This protocol is designed to evaluate the feasibility and acceptability of a barbershop-based HIV prevention intervention and test its preliminary effect on male engagement in HIV prevention services. The intervention was piloted among men in Kalangala district, Uganda—a population that may benefit significantly from expanded HIV prevention efforts. Barbershops, which serve as popular gathering places for men in many settings, especially in African communities, provide a promising platform for this intervention. The intervention involved 3 main components delivered by the barbers: HIV education and referral information for HIV services, distribution of HIV self-test kits through barbershops, and barber-led peer group sessions. Given the documented barriers men face while engaging in HIV services [26,73]—such as stigma around testing, services that are often tailored to women, inconvenient service locations and hours [29,30,33-37,74]—this protocol offers an opportunity to explore a novel, community-based strategy to address these gaps. Ultimately, this approach aims to reach a historically underserved population within the HIV prevention and care continuum.

Barbershops may be convenient and ideal venues for health promotion [75]. Earlier studies showed that health initiatives based in barbershops improved condom use [51] and high blood pressure control [76,77]. More recently, a trustworthy relationship between barbers and their clients has been reported as a facilitator for collaboration with barbershop businesses to reach Black men for HIVST distribution [59]. Similarly, salon-based PrEP initiatives also improved PrEP knowledge and uptake among Black women in the United States [78]. These successes suggest that salon or barbershop-based interventions may be transferable across settings, informing the development of similar HIV prevention initiatives targeting barbershop-going men in Uganda and other settings. HPTN 111 was implemented in a rural fishing community, where the lessons learned may be transferable to similar rural contexts, although they may not be generalizable to urban and nonfishing communities. The study area was selected due to the high HIV burden in the Ugandan fishing communities [73] and the demographic reality that more men than women live in these areas. This imbalance has contributed to high rates of transactional sex, with the majority of men being unaware of their HIV status [79-82].

Peer support in this study included barber-led education and barber-led group sessions. Peer support draws on shared personal experience to provide knowledge, social interaction, emotional assistance, or practical help, often in a mutually beneficial way, where the source of support is a peer with relevant lived experience [83-85]. In this study, the barbers provided education, actively listened, built rapport, and offered emotional and social support to promote behavioral change, consistent with recommended peer support practices [51-53]. The intervention used status-neutral education during barber-client interactions, a strategy shown to be both feasible and effective in community-based interventions [86,87]. The barber-delivered peer support was provided face-to-face during routine haircuts, in line with evidence that peer support is most effective when delivered regularly in familiar and comfortable settings [88,89]. Barbers also facilitated group sessions, allowing men to discuss HIV prevention practices among peers. We anticipated that these regular interactions would improve HIV prevention knowledge and enhance men’s engagement in HIV prevention practices.

Male-led, community-based interventions can help overcome stigma and masculinity-related barriers that typically deter men from using HIV services [40,44]. Moreover, during and after adolescence, men often rely on peer networks for support, guidance, and advice in the development of a sense of belonging and identity [57,90]. This study leverages this notion since most barbers in this setting are men. Further, qualitative evidence suggests that peer counseling fosters trust and confidence toward HIV testing, including HIVST [51-53].

HIVST is a robust tool that could benefit historically hard-to-reach populations in need of HIV prevention and care services. However, concerns remain about whether individuals who use HIVST are adequately linked to follow-up care. HPTN 111 seeks to address this gap via barbershops to expand access to HIVST among men who may face barriers in obtaining it from health facilities and improve their linkage to HIV prevention services and care.

Data on the feasibility and acceptability of the intervention, collected through quantitative surveys and qualitative interviews, will provide critical insights into barriers and facilitators to implementation. Systematic evaluation and analysis of these data will inform refinement of the intervention package and guide the development of strategies for broader scale-up. In addition, findings from both predefined quantitative objectives and exploratory analyses will help assess the potential effectiveness of the intervention and identify areas for improvement. Collectively, these data will generate practical insights on the provision of barbershop-based HIV services in high HIV-burden fishing communities and similar settings.

Subsequently, if successful, this intervention will contribute to the community-based decentralization of HIV services by enhancing men’s awareness of HIV and risk-reduction strategies, increasing access to and use of HIV testing services, reducing HIV-related stigma associated with testing, and strengthening linkage to HIV prevention and care services. Overall, the intervention will improve male engagement in HIV prevention and care, reduce HIV infections among men, and ultimately decrease HIV transmission to adolescent girls and young women.

This protocol is limited by the absence of prespecified progression criteria, as the novelty of the intervention meant there was no sufficient prior data to serve as a benchmark. Nevertheless, the protocol clearly outlines how feasibility and acceptability will be assessed and how the results will inform subsequent steps, in line with guidance from the literature [41,91].

In conclusion, the HPTN 111 (TRIM) study will answer the question of whether the barbershop-based HIV prevention initiative is feasible, acceptable, and provide insight into preliminary effectiveness in improving HIV testing, promoting behavioral change, and minimizing HIV and STI incidence. This study highlights the potential of barbershops as accessible, trusted, and far-reaching venues for delivering HIV prevention services to men. This barber-led intervention is anticipated to effectively engage and sustain heterosexual men in HIV prevention and care. If found feasible and acceptable, the data obtained will inform modifications and guide the implementation of the intervention in a future larger-scale effectiveness trial.

## Data Availability

The datasets generated or analyzed during this study are not publicly available due to the pending publication of the primary manuscript. Protocol and letter of amendment files are available at the HIV Prevention Trials Network [92].

## Appendix

Multimedia Appendix 1 HIV Prevention Trials Network 111 protocol V2.0.

Multimedia Appendix 2 Sample participant and barber informed consent forms.

Multimedia Appendix 3 HIV Prevention Trials Network 111 (Testing a Barbershop-based HIV Prevention Initiative Among Men) participant in-depth interview guide.

Multimedia Appendix 4 Participant schedule of events.

Multimedia Appendix 5 Barber schedule of events.

Multimedia Appendix 6 HIV Prevention Trials Network 111 (Testing a Barbershop-based HIV Prevention Initiative Among Men) barber in-depth interview guide.

Multimedia Appendix 7 HIV Prevention Trials Network 111 feasibility and acceptability scoring details.

Multimedia Appendix 8 Peer review report by the HIV Prevention Trials Network (HPTN) Science Review Committee (SRC), National Institute of Allergy and Infectious Diseases (NIAID) (National Institutes of Health, USA).

## Abbreviations

ACASI: audio computer–assisted self-interview
CHTS: community-based HIV testing services
COM-B: Capability, Opportunity, and Motivation Model of Behavior Change
CRF: case report form
DAIDS: Division of AIDS
HIVST: HIV self-testing
HPTN: HIV Prevention Trials Network
ICF: informed consent form
IRB: institutional review board
NIAID: National Institute of Allergy and Infectious Diseases
PrEP: preexposure prophylaxis
REDCap: Research Electronic Data Capture
SMC: study monitoring committee
SoC: standard of care
SPIRIT: Standardized Protocol Items: Recommendations for Interventional Trials
SSA: sub-Saharan Africa
STI: sexually transmitted infection
TRIM: Testing a Barbershop-based HIV Prevention Initiative Among Men
UNAIDS: Joint United Nations Program on HIV/AIDS
