# Supplementary material for: Feasibility and Acceptability of Barbershop-Based HIV Prevention Among Heterosexual Men in Kalangala Islands, Uganda: Protocol for a Cluster Randomized Trial (HPTN 111)
Source: JMIR Res Protoc. 2026 Apr 17;15:e87612. doi: 10.2196/87612 (PMC13135168; doi:10.2196/87612)
Supplement: Multimedia Appendix 1 [file resprot_v15i1e87612_app1.docx]

HPTN 111

Feasibility and Acceptability of a Barbershop Based HIV Prevention Initiative Among Heterosexual Men in Kalangala Islands, Uganda: A Cluster Randomized Trial

DAIDS Document ID: 39062

A Study by the HIV Prevention Trials Network (HPTN)

Sponsored by:

Division of AIDS (DAIDS), United States (US) National Institute
of Allergy and Infectious Diseases (NIAID), US National Institutes of Health (NIH)

Non-IND Study

Protocol Chair:

Zubair Lukyamuzi, MBChB, MPH, Msc

Protocol Co-Chair:

Brenda Gati Mirembe, MBChB, Dip. Epi, Msc. Epi

Version 2.0

31 January 2025

**HPTN 111**

**Feasibility and Acceptability of a Barbershop Based HIV Prevention Initiative Among Heterosexual Men in Kalangala Islands, Uganda: A Cluster Randomized Trial**

TABLE OF CONTENTS

Contents

[1.0 Introduction 13](#_Toc187834199)

[1.1 Background 13](#_Toc187834200)

[1.2 African men in the HIV care cascade 14](#_Toc187834201)

[1.3 Evidence-based HIV prevention tools 14](#_Toc187834202)

[1.4 Innovative approaches to reach men 17](#_Toc187834203)

[1.5 Rationale 18](#_Toc187834204)

[2.0 Study Objectives 19](#_Toc187834205)

[2.1 Primary Objective 19](#_Toc187834206)

[2.2 Secondary Objectives 20](#_Toc187834207)

[2.3 Exploratory Objective 20](#_Toc187834208)

[3.0 Study Design 20](#_Toc187834209)

[3.1 Participating Sites 21](#_Toc187834210)

[3.1.1 Barbershops 22](#_Toc187834211)

[3.1.2 Study Clinic Site 23](#_Toc187834212)

[3.2 Study Duration 23](#_Toc187834213)

[4.0 Study Population 23](#_Toc187834214)

[4.1 Participants 23](#_Toc187834215)

[4.1.1 Inclusion Criteria 23](#_Toc187834216)

[4.1.2 Exclusion Criteria 24](#_Toc187834217)

[4.1.3 Recruitment Process 24](#_Toc187834218)

[4.1.4 Co-Enrollment Guidelines 25](#_Toc187834219)

[4.1.5 Participant Retention 25](#_Toc187834220)

[4.2 Barbers 26](#_Toc187834221)

[4.3 Participant Withdrawal 26](#_Toc187834222)

[5.0 Study Procedures for Participants 26](#_Toc187834223)

[5.1 Pre-Screening Assessment 27](#_Toc187834224)

[5.2 Screening and Enrollment Visit 27](#_Toc187834225)

[5.3 Follow-Up Study Visits (Week 26 & 52) 29](#_Toc187834226)

[5.4 Procedures for Participants with Suspected or Confirmed HIV Infection 29](#_Toc187834227)

[5.5 Sexually Transmitted Infections (STIs) 30](#_Toc187834228)

[5.6 Questionnaires Data Collection 30](#_Toc187834229)

[5.7 Interim Contacts and Visits 30](#_Toc187834230)

[5.8 Barbershop-Based Intervention Procedures 31](#_Toc187834231)

[5.9 Qualitative Data Collection 31](#_Toc187834232)

[5.10 Study Visit Windows 32](#_Toc187834233)

[6.0 Study Procedures for Barbers 32](#_Toc187834234)

[6.1 Barber Study Initiation 32](#_Toc187834235)

[6.2 Barber Training 33](#_Toc187834236)

[6.3 Questionnaires Data Collection for Barbers 33](#_Toc187834237)

[6.4 Qualitative Data Collection for Barbers 34](#_Toc187834238)

[6.5 Barber Delivery of the Intervention 34](#_Toc187834239)

[7.0 Safety Monitoring 35](#_Toc187834240)

[7.1 Safety Monitoring 35](#_Toc187834241)

[7.2 Social Impact Reporting 35](#_Toc187834242)

[7.3 Study Monitoring Committee Review 36](#_Toc187834243)

[8.0 Statistical Considerations 36](#_Toc187834244)

[8.1 Objectives and Endpoints 36](#_Toc187834245)

[8.1.1 Primary Objective and Endpoints 36](#_Toc187834246)

[8.1.2 Secondary Objectives and Endpoints 36](#_Toc187834247)

[8.1.3 Exploratory Objective and Endpoints 37](#_Toc187834248)

[8.2 Sample Size 38](#_Toc187834249)

[8.3 Accrual and Retention 38](#_Toc187834250)

[8.4 Random Assignment/Study Arm Assignment 39](#_Toc187834251)

[8.5 Blinding 39](#_Toc187834252)

[8.6 Statistical Analysis 39](#_Toc187834253)

[8.6.1 Primary Analyses 39](#_Toc187834254)

[8.6.2 Secondary Analyses 40](#_Toc187834255)

[8.6.3 Exploratory Analyses 40](#_Toc187834256)

[8.6.4 Qualitative Analyses 40](#_Toc187834257)

[8.6.5 Interim Stopping Plan 41](#_Toc187834258)

[9.0 Laboratory Specimens and Biohazard Containment 41](#_Toc187834259)

[9.1 Local Laboratory Specimens 41](#_Toc187834260)

[9.1.1 Specimen Storage 42](#_Toc187834261)

[9.2 Quality Control and Quality Assurance Procedures 42](#_Toc187834262)

[9.3 HIV Diagnostic Testing 42](#_Toc187834263)

[9.4 Biohazard Containment 42](#_Toc187834264)

[10.0 Human Subjects Considerations 43](#_Toc187834265)

[10.1 Ethical Review 43](#_Toc187834266)

[10.2 Informed Consent 43](#_Toc187834267)

[10.3 Risks 44](#_Toc187834268)

[10.4 Benefits 44](#_Toc187834269)

[10.5 Incentives 45](#_Toc187834270)

[10.6 Confidentiality 45](#_Toc187834271)

[10.7 Study Discontinuation 46](#_Toc187834272)

[11.0 Administrative Procedures 46](#_Toc187834273)

[11.1 Protocol Registration 46](#_Toc187834274)

[11.2 Study Activation 46](#_Toc187834275)

[11.3 Study Coordination 47](#_Toc187834276)

[11.4 Study Monitoring 47](#_Toc187834277)

[11.5 Protocol Compliance 47](#_Toc187834278)

[11.6 Investigator’s Records 47](#_Toc187834279)

[11.7 Use of Information and Publications 48](#_Toc187834280)

[11.8 ClinicalTrials.gov 48](#_Toc187834281)

**HPTN 111**

**Feasibility and Acceptability of a Barbershop Based HIV Prevention Initiative Among Heterosexual Men in Kalangala Islands, Uganda: A Cluster Randomized Trial**

Protocol Signature Page

**DAIDS Document ID # 39062**

**Version 2.0**

**31 January 2025**

I will conduct the study in accordance with the provisions of this protocol and all applicable protocol-related documents. I agree to conduct this study in compliance with United States (US) Health and Human Service regulations (45 CFR 46); applicable US Food and Drug Administration regulations; standards of the International Conference on Harmonization Guideline for Good Clinical Practice (E6); Institutional Review Board/Ethics Committee determinations; all applicable in-country, state, and local laws and regulations; and other applicable requirements (e.g., US National Institutes of Health, Division of AIDS) and institutional policies.

I have read and understand the information in this protocol and will ensure that all associates, colleagues, and employees assisting in the conduct of the study are informed about the obligations incurred by their contribution to the study.

____________________________________

Name of Investigator of Record (print name)

____________________________________ _______________________

Signature of Investigator of Record Date (DD/MONTH/YYYY)

**HPTN 111**

**Feasibility and Acceptability of a Barbershop Based HIV Prevention Initiative Among Heterosexual Men in Kalangala Islands, Uganda: A Cluster Randomized Trial**

List of Abbreviations and Acronyms

|  |  |
| --- | --- |
| ACASI | Audio computer assisted self-interview |
| AIDS | Acquired immunodeficiency syndrome |
| ARV | Antiretroviral |
| CAB | Community Advisory Board |
| CFR | Code of Federal Regulations |
| CLIA | Clinical Laboratory Improvement Amendments of 1988 |
| CRM | Clinical Research Manager |
| CRMS | (DAIDS) Clinical Research Management System |
| CRPMC | (DAIDS) Clinical Research Products Management Center |
| CRF | Case Report Form |
| DAIDS | Division of AIDS |
| DHHS | US Department of Health and Human Services |
| EC | Ethics Committee |
| EQA | External Quality Assurance |
| EWG | (HPTN) Ethics Working Group |
| FDA | (United States) Food and Drug Administration |
| GCLP | Good Clinical Laboratory Practices |
| HIV  HIVST | Human Immunodeficiency Virus  HIV self-test |
| HPTN | HIV Prevention Trials Network |
| ICF | Informed consent form |
| IoR | Investigator of Record |
| IQA | (DAIDS) Immunology Quality Assurance |
| IRB | Institutional Review Board |
| LAPrEP | Long-Acting PrEP |
| LC | (HPTN) Laboratory Center |
| LDMS | Laboratory Data Management System |
| LL | Local laboratory |
| LOC | (HPTN) Leadership and Operations Center |
| MO | (DAIDS) Medical Officer |
| MOP | (HPTN) Manual of Operations |
| MOU | Memorandum of understanding |
| MU-JHU | Makerere University – Johns Hopkins University |
| NAAT | Nucleic Acid Amplification Test |
| NIAID | (United States) National Institute of Allergy and Infectious Diseases |
| NIH | (United States) National Institutes of Health |
| PEP | (HIV) Post-exposure prophylaxis |
| PLHIV | People living with HIV |
| PrEP | (HIV) Pre-exposure prophylaxis |
| PRO | Protocol Registration Office |
| RE | Regulatory entity |
| RNA | Ribonucleic acid |
| RPR | Rapid Plasma Reagin |
| RSC | Regulatory Support Center |
| SDMC | (HPTN) Statistical and Data Management Center |
| SMC | Study Monitoring Committee |
| STI | Sexually transmitted infection |
| SOP | Standard Operating Procedures |
| SSP | Study Specific Procedures |
| TPHA | TP Hem-Agglutination |
| QA | Quality Assurance |
| QC | Quality Control |
| UNAIDS | United Nations Programme on HIV/AIDS |
| UNCST | Uganda National Council for Science & Technology |
| US | United States |
| UVRI | Uganda Virus Research Institute |
| VMMC | Voluntary medical male circumcision |
|  |  |
|  |  |
|  |  |
|  |  |
|  |  |
|  |  |

**HPTN 111**

**Feasibility and Acceptability of a Barbershop Based HIV Prevention Initiative Among Heterosexual Men in Kalangala Islands, Uganda: A Cluster Randomized Trial**

Protocol Team Roster

**PROTOCOL CHAIR
Zubair Lukyamuzi, MBChB, MPH, Msc. Pharm.**

Makerere University, Johns Hopkins University Collaboration (MU-JHU)

Phone: +256 771612323/704818950

Email: zlukyamuzi@mujhu.org

**PROTOCOL CO-CHAIR**

**Brenda Gati Mirembe, MBChB, Dip. Epi, Msc. Epi**

Makerere University, Johns Hopkins University Collaboration (MU-JHU)
Phone: +256 772881922/700390476

Email: bgati@mujhu.org

*Members listed in alphabetical order*:

**Maggie Albano, MA**

Community Program Associate

HPTN LOC, FHI 360

Phone: +1 202-464-3753

Email: malbano@fhi360.org

**Deborah Donnell, PhD**

Statistician

SCHARP, FHCRC

Phone: 206-667-5661

Email: deborah@scharp.org

**Molly Dyer, MPH**

Community Program Associate

HPTN LOC, FHI 360

Phone: 919-321-3851

Email: mdyer@fhi360.org

**Juliane Etima, MA**

Behavioral Scientist

MU-JHU Research Collaboration

Phone: +256-772-4368 29

Email: jetima@mujhu.org

**Jayla Harris-Wisecarver, MPH**

Clinical Research Manager

HPTN LOC, FHI 360

Phone: 919-321-3866

Email: jharris@fhi360.org

**Kameron Jacobs, MA**

Communications Specialist

HPTN LOC, FHI 360

Phone: 253-219-1041

Email: kjacobs@fhi360.org

**Doreen Kemigisha, MPH**

CWG Representative

Makerere University - Johns Hopkins Research Collaboration (MU-JHU)

Phone: +256 782493311/702251804

Email: [dkemigisha@mujhu.org](mailto:dkemigisha@mujhu.org)

**Johan H. Melendez, PhD**

HPTN LC STI Representative

HPTN LC - JHU

Phone: 410-614-0924

Email: [jmelend3@jhmi.edu](mailto:jmelend3@jhmi.edu)

**Ayana Moore, PhD**

Associate Director

HPTN LOC, FHI 360

Phone: 919-321-3468

Email: amoore@fhi360.org

**Heba Mostafa, MD, PhD, D(ABMM)**

HPTN LC Virologist

HPTN LC JHU

Phone: 410-955-5077

Email: hmostaf2@jhmi.edu

**Andrew Mujugira MBChB, PhD**

Senior Research Scientist

Makerere University Infectious Diseases Institute

Phone: +256-754-173 225

Email: amujugira@idi.co.ug

**Clemensia Nakabiito, MBChB, Mmed**

CRS Leader

MUJHU Care Ltd, MUJHU Research Collaboration

Phone: +256 200 404 100

Email: cnakabiito@mujhu.org

**Rita Nakalega, MBChB, MPH, MMED**

Investigator

MUJHU Research Collaboration

Phone: +256-782-179-622

Email: rnakalega@mujhu.org

**Kenneth Ngure MPH, PhD**

Behavioral Scientist

School of Public Health, JKUAT, Nairobi Kenya

Phone: +254-722-3622 19

Email: kngure@pipsthika.org

**Estelle Piwowar-Manning, MT**

HPTN LC Deputy Director, QA Core

HPTN LC, JHU

Phone: 410-614-6736

Email: epiwowa@jhmi.edu

**Hans Speigel, MD, PhD, DTMH, FAAP**

Sr. Medical Officer

PMPRB/Prevention Sciences Program, Pediatric Infectious Diseases, Contractor, Kelly Government Solutions, DAIDS, NIAID, NIH

Phone: 240-292-4633

Email: hans.spiegel@nih.gov

**Emily Voldal, PhD**

Protocol statistician

Fred Hutchinson Cancer Center

Phone: 206-667-6423

Email: evoldal@fredhutch.org

**HPTN 111**

**Feasibility and Acceptability of a Barbershop Based HIV Prevention Initiative Among Heterosexual Men in Kalangala Islands, Uganda: A Cluster Randomized Trial**

Schema

| **Purpose:** | To test community-based approaches to engage heterosexual men at risk for HIV and specifically to assess the feasibility and acceptability of a barbershop based HIV prevention program. |
| --- | --- |
| **Design:** | This is a cluster randomized study. Eighteen barbershops in the Kalangala district, Uganda will be purposively selected to participate in the study and will be randomized 2:1 to participate in the barbershop-based HIV prevention initiative (intervention) or standard-of-care (control). Individual participants will be recruited from purposively selected barbershops. Participants enrolled from the intervention shops will receive the barbershop based HIV prevention initiative. Participants enrolled from the control shops will receive standard-of-care HIV prevention services. |
| **Population:** | Heterosexual men behaviorally vulnerable to HIV, ages ≥ 16 years |
| **Study Size:** | Up to 250 men |
| **Study Duration:** | Approximately 24 months for study start-up, participant recruitment, and follow-up, with individual participants followed for 6-12 months. |
| **Study Location:** | Kalangala district (Islands), Uganda |
| **Study Regimen:** | Intervention group: participants recruited from intervention barbershops will receive the barbershop-based HIV prevention initiative. The trained barber will provide the following services during regular haircut services: general, status-neutral HIV education, HIV self-test kits, and information about where to receive HIV prevention services. The barber will also lead peer support group education every two months for clients enrolled in the study.  Control group: participants recruited from control barbershops will receive standard-of-care HIV prevention services which include facility-based HIV risk reduction counseling and testing and providing information about facility distributed HIV self-test kits.  All participants in both groups will complete study clinic visits for scheduled study procedures which include bio-behavioral data collection, HIV counseling and testing, and STI testing. Participants will be followed for 12 months. |
| **Primary Objective:** | - To evaluate the feasibility and acceptability of a barbershop-based HIV prevention initiative |
| **Secondary Objectives:** | - To compare completion of self-initiated HIV testing between intervention and control groups - To evaluate the preliminary effectiveness of the intervention on change in behaviors associated with HIV acquisition - To compare interest in or use of HIV prevention services between intervention and control groups - To assess the interest in long-acting PrEP among all participants and by study arm |
| **Exploratory Objective:** | - To evaluate the preliminary effectiveness of the intervention on incident STIs |

**HPTN 111**

**Feasibility and Acceptability of a Barbershop Based HIV Prevention Initiative Among Heterosexual Men in Kalangala Islands, Uganda: A Cluster Randomized Trial**

Overview of Study Design


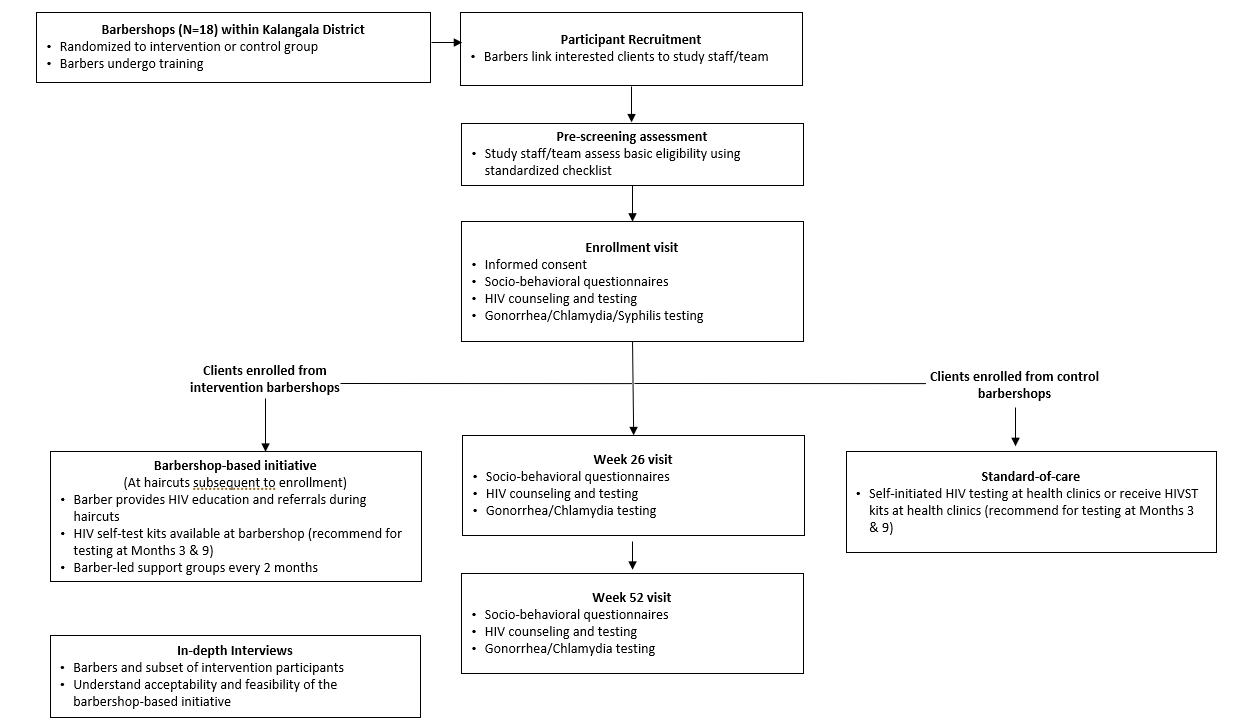


# Introduction

## Background

Human immunodeficiency virus (HIV) remains a major public health problem worldwide, with 38 million people globally infected by 2020 [[1](#_ENREF_1)]. Despite the global commitment to reduce AIDS-related deaths and new HIV infections to fewer than 500,000, 650,000 AIDS-related deaths and 1.5 million new infections were registered in 2021—which respectively resulted in 150,000 more AIDS-related deaths and 1 million more new infections than the global targets[[2](#_ENREF_2)]. These data showed that the AIDS response and the ambitious target of ending HIV/AIDs by 2030 are in danger [[2](#_ENREF_2), [3](#_ENREF_3)]. According to UNAIDS 2021 estimates, there are 20.6 million people living with HIV (PLHIV) in Eastern and Southern Africa, the region with the highest HIV burden. Although men represent only 37% (7.7 out of 20.6 million) of HIV infections, they were 14% more likely to die from AIDS related causes compared to women in 2020 [[2](#_ENREF_2), [3](#_ENREF_3)].

Through various strategies, such as increasing the number of PLHIV who are aware of their HIV status to 95%, [[3](#_ENREF_3), [4](#_ENREF_4)] UNAIDs and partners are focused to end HIV/AIDS. However, over the past decade it has become increasingly clear that we are missing men in the AIDS response [[5](#_ENREF_5)]. Men and boys are less likely to test for HIV, to initiate antiretroviral therapy (ART) and to remain engaged in care, therefore dying of AIDS-related illnesses and many other diseases at rates that are disproportionately higher than their female counterparts [[2](#_ENREF_2), [6](#_ENREF_6), [7](#_ENREF_7)]. In many countries, more than half of men aged 24-35 years living with HIV are unaware of their status and therefore not on treatment. When compared to women globally, there are 1 million more men living with HIV who are unaware of their HIV status and 1.8 million more men not on treatment [[3](#_ENREF_3), [4](#_ENREF_4)], which increases the risk of HIV transmission. This is due to the fact that an HIV infected man transmits HIV two times more than an infected woman [[8](#_ENREF_8)]. Therefore, the diagnosis of HIV in men is essential for promoting men's health and breaking the cycle of HIV transmission [[6](#_ENREF_6), [9](#_ENREF_9)].

A significant body of research and experience shows that a range of complex, multilevel factors contribute to men’s and boys’ low uptake of HIV-related services [[6](#_ENREF_6), [7](#_ENREF_7), [10](#_ENREF_10), [11](#_ENREF_11)]. Barriers to male partner involvement in HIV care services include fear of knowing one’s status and related stigma [[12](#_ENREF_12), [13](#_ENREF_13)]. Additional barriers are the product of prevailing harmful cultural, social, and gender norms—such as equating illness with “weakness” and viewing clinical settings as “female spaces”—which encourage men to take excessive risks and be over-controlling [[14](#_ENREF_14), [15](#_ENREF_15)]. However, there are many other factors that contribute to the status quo, which include lack of universal men and boys’ entry points to health systems that women and girls generally have. For instance, HIV prevention and care services in eastern and southern Africa focus more on women of reproductive age, and therefore, reproductive, maternal and child health services offer ideal entry points to HIV services which don’t exist for men and boys [[6](#_ENREF_6)]. Barriers within the healthcare system for men and boys include limited opening hours, facility-based service delivery models which further restrict access for men who work outside their communities during the day, and inadequate public health strategies or approaches that are aimed at improving men’s access to health and HIV services [[6](#_ENREF_6), [7](#_ENREF_7)].

To effectively achieve reduction in HIV incidence and prevalence among women and adolescent girls, a commensurate 80% reduction in new HIV infections among men by 2025 should be achieved, and this can be done by having 90% of men in high-prevalence settings access combination HIV prevention, 90% of men use condoms during sex with non-regular partners, and 90% of men aged 15-29 getting circumcised in priority countries [[6](#_ENREF_6)]. The health and lives of men and boys are intertwined with those of women and girls. Gender inequalities, cultural, and social norms about femininities and masculinities disproportionately affect women’s and girls’ sexual and reproductive health and rights, and consequently, only 55% of women have autonomy in reproductive health decision-making [[16](#_ENREF_16), [17](#_ENREF_17)]. This contributes significantly to the continued women’s and girls’ vulnerabilities to HIV infection [[18](#_ENREF_18), [19](#_ENREF_19)].

Therefore, it is currently recommended that the architecture of health service delivery urgently be reviewed to overcome the barriers to HIV services among men. Health institutions should promote access to services for men and boys and have HIV-related health systems, policies and strategies that include men, especially those more vulnerable to HIV. A broader supportive enabling environment for men to access health services is also critical [[6](#_ENREF_6)].

## African men in the HIV care cascade

In Eastern and Southern Africa, the HIV epidemic is largely heterosexually transmitted and heterosexual men are greatly affected [[20](#_ENREF_20), [21](#_ENREF_21)]. However, African men are missing from the HIV care cascade and have been left behind in the HIV response [[22](#_ENREF_22)] which has greatly affected clinical and public health outcomes and targets [[23](#_ENREF_23)]. For instance, HIV status knowledge is lower among men than women, leading to a higher proportion of undiagnosed HIV infection in men, [[24](#_ENREF_24), [25](#_ENREF_25)] and men who are unaware of their HIV status increase the risk of HIV transmission [[26](#_ENREF_26)]. A meta-analysis of 129 studies from sub-Saharan Africa found that 49% of men did not know their HIV status (95% CI: 0.41-0.58), 58% were not on treatment (95% CI: 0.51-0.65), and 21% on treatment were not virally suppressed (95% CI: 0.19-0.23) [[22](#_ENREF_22)]. Thus, the proportions of African men meeting UNAIDS 95-95-95 goals are only 51-42-79. In Uganda, 12% of men living with HIV are unaware of their HIV status compared to 4% of women [[3](#_ENREF_3), [4](#_ENREF_4)]. Fishing communities in Uganda have a high HIV burden; HIV prevalence in Kalangala Islands (the largest fishing community in Uganda) is approximately three times higher than the national prevalence of 6.2% with an HIV incidence of 5.2 per 100 person years; therefore, the prevalence of HIV in this setting is estimated at 16%. Furthermore, the majority of the islanders are men, with a 3:1 ratio of men to women and 48.4% of islander men are unaware of their HIV status [[27-30](#_ENREF_27)]. Although the national HIV incidence among men is significantly lower than that of women with a 47% difference [[28](#_ENREF_28)], in Kalangala, the new infections among men were only slightly lower than those of women in 2022. Specifically, there were 418 new infections among men aged at least 15 years compared to 470 new infections among women of the same age group [[31](#_ENREF_31)].

In addition to other multi-level factors that contribute to poor uptake of HIV services by men, African men largely believe that their status is the same as their partners (i.e., testing by ‘proxy’), [[32](#_ENREF_32)] and have serious concern about time away from work and the impact on their income [[13](#_ENREF_13), [33-36](#_ENREF_33)]. Therefore, offering men evidence-based, near location, and self-controlled HIV prevention services could motivate uptake and use of HIV prevention services.

## Evidence-based HIV prevention tools

Various evidence-based interventions and tools have been developed to improve HIV prevention and care and these include peer support groups, peer led counseling, and HIV self-testing.

*Peer support groups*

Peer support involves people drawing on shared personal experience to provide knowledge, social interaction, emotional assistance, or practical help to each other, often in a way that is mutually beneficial. Peer support is different from other types of support because the source of support is a similar person with relevant experience[[37-39](#_ENREF_37)]. Various categories of target people may be involved in peer support groups including people vulnerable to HIV. Support in peer groups may be set up by peers themselves or professionals; peer support sessions may be facilitated by peers alone or a professional; and peers may be trained. The type of support provided may include information and education provision, emotional, and social support. Support activities may include discussion, listening, mentoring, befriending, and peer delivered services such as HIV risk reduction counseling. Peer support may be delivered through telephone and face to face, and it may involve small groups of people meeting at a desired location for a specific time on a regular basis for a given period [[40](#_ENREF_40)].

Peer-support in HIV prevention and care has a long history. Already in the 1980s, groups of people living with HIV were supporting each other, sharing knowledge, and advocating for better treatment and care. Today, there are diverse terms for peer-support interventions and somewhat different conceptualizations, but since the introduction of ART, peer-support has become a more tailored, person-centered outreach to provide linkage to and adherence to HIV care, as well as support people living with HIV in taking an active role in self-management of their chronic health condition [[41](#_ENREF_41)]. Positively UK specifies that peer-support is a relationship in which people are equal partners and the focus is on mutual learning and growth [[42](#_ENREF_42)]. Dennis et al. similarly defines peer-support as “the giving of assistance and encouragement by an individual considered equal” [[43](#_ENREF_43)] A peer is thus someone who shares common characteristics (e.g., age, sex, disease status) with the supported individual, such that the peer can relate to and empathize with the individual on a level that a non-peer would be unable to [[44](#_ENREF_44)]. Peer-support fits within a social support model which states that, peer-support has the potential to reduce feelings of isolation and loneliness, provide information, and promote behaviors that improve personal health, well-being, and health practices [[45](#_ENREF_45)].

These principles of peer support have also been adapted for use in HIV prevention, for example in the FACTS 001 tenofovir gel trial in nine sites across South Africa [[46](#_ENREF_46), [47](#_ENREF_47)]. The trial used clubs to help the young women tackle immediate adherence challenges, such as managing side effects, and addressed underlying socio-structural barriers to gel use.

Peer support frequently includes education. Studies have found that having a higher HIV/AIDS-related knowledge was associated with improved engagement with HIV testing services and reduced sexual risk behavior [[48](#_ENREF_48), [49](#_ENREF_49)]. Educational strategies for men may also work best if they are driven by other men in order to fight the stigma and masculinity challenges that prevent men from testing for HIV [[50](#_ENREF_50), [51](#_ENREF_51)]. Educational programs may include basic HIV information, risk reduction strategies, and confidentiality [[48](#_ENREF_48), [52](#_ENREF_52), [53](#_ENREF_53)]. Different studies have indicated that such educational interventions should involve local leaders, including chiefs [[51](#_ENREF_51)] and/or be part of the community-based outreach campaigns. However, there remains an educational gap on HIV programs that are specifically tailored for men [[54](#_ENREF_54)]. Therefore, barber-led HIV education in peer groups may contribute to the bridging of this gap.

*Peer-based counseling*

Peer-based counseling has been noted to improve risk reduction behavior change and uptake of HIV prevention and care in various populations [[55-57](#_ENREF_55)]. Peer intervention approaches which employ the use of individuals who are similar to the target individuals in terms of social or demographic characteristics are a common strategy to promote and sustain HIV-risk reduction behaviors [[58](#_ENREF_58), [59](#_ENREF_59)]. Peers play various roles in HIV prevention and treatment such as offering social support, education of patients, and offering referrals; studies show positive effects of peer interventions on improving linkage and retention of HIV-care and programs [[60](#_ENREF_60)]. Compared to regular healthcare professionals, peer-led counseling strategies may be more efficient and cost effective in influencing behavioral modifications by addressing psychological needs without discrimination or stigmatization. Qualitative evidence suggests that peer counselling may help to build confidence and trust towards HIV prevention services including HIV self-testing (HIVST) [[55-57](#_ENREF_55)]. The use of peers or other men to convey information and deliver psycho-educational materials has been reported to improve health and help seeking behaviors in mental health. This has specifically assisted men to recognize and adopt active problem solving and motivating behavior change [[61](#_ENREF_61)]. Moreover, during and after adolescence, men turn to peers for support, guidance, and advice in developing a sense of belonging and identity [[62](#_ENREF_62), [63](#_ENREF_63)]. Therefore, use of barbers as peers in this study is a postulated powerful tool for increasing HIV testing rates and improving HIV risk reduction behaviors among men, particularly those who may face barriers to clinic-based HIV prevention services.

*HIV self-testing*

HIVST is a robust, evidence-based, and self-controlled HIV prevention tool that is currently available in many countries to enhance equity in HIV services and increase HIV testing. HIVST is an innovative approach for reaching key populations, recommended by the World Health Organization (WHO) as an additional HIV testing approach, and is feasible and acceptable among various populations, including among heterosexual men [[64-66](#_ENREF_64)]. HIVST has been rolled out through a variety of delivery models and more than 4.8 million test kits have been provided across 11 sub-Saharan African countries, including Uganda [[67](#_ENREF_67)]. A recent systematic review of studies in sub-Saharan Africa found that while uptake of testing has been high, many questions remain about the effectiveness of HIVST to reach populations that are traditionally unsuccessfully engaged particularly men and if HIVST facilitates their linkage to HIV care or prevention services [[68](#_ENREF_68)]. Therefore, innovative approaches to reach men are urgently needed.

*Use of long-Acting PrEP*

Long-Acting Pre-Exposure Prophylaxis (LAPrEP) refers to long-acting HIV prevention tools that eliminate the need for daily dosing. These tools can be swallowed, inserted, injected, infused, or implanted in the body to provide sustained protection against HIV acquisition, ranging from once a month to once a year [[69](#_ENREF_69), [70](#_ENREF_70)]. Currently, individuals at risk of HIV can reduce their chances of acquiring the virus by taking HIV prevention medication, known as pre-exposure prophylaxis (PrEP), as prescribed [[71](#_ENREF_71)]. When taken daily, oral PrEP pills can effectively prevent HIV from establishing itself in the body [[72](#_ENREF_72)]. However, adherence to oral PrEP remains a significant challenge [[72-74](#_ENREF_72)], highlighting the need for long-acting forms of PrEP. To address this need, studies are underway to develop and test various long-acting forms of HIV prevention, including oral, insertable, injectable, infusible, and implantable options that provide protection for at least a month [[70](#_ENREF_70), [75](#_ENREF_75)]. The ultimate goal is to offer individuals a range of acceptable, discreet, and convenient choices for highly effective HIV prevention. Notably, cabotegravir (integrase strand transfer inhibitor) injection is the only long-acting injectable PrEP approved by the U.S. Food and Drug Administration (FDA), World Health Organization (WHO), and other regulatory bodies [[76](#_ENREF_76), [77](#_ENREF_77)]. Recently, lenacapavir (capsid inhibitor) has demonstrated remarkable efficacy as a long-acting injectable PrEP, with 100% effectiveness in preventing HIV acquisition in cisgender women and 96% effectiveness in men who have sex with men, gay men, transgender men, transgender women, and gender non-binary individuals [[78-80](#_ENREF_78)]. While LAPrEP has proven effective and acceptable in various populations, including transgender men and men who have sex with men[[80](#_ENREF_80)], its effectiveness and acceptability among heterosexual men at risk of HIV in sub-Saharan Africa, where HIV transmission is predominantly heterosexual, remains unknown.

## Innovative approaches to reach men

Innovative approaches are needed to address the challenges associated with poor male engagement in care and to break the cycle of HIV transmission. Offering men evidence-based, self-controlled HIV prevention tools, in a comfortable and informal setting, could motivate HIV testing uptake and linkage to care. Community HIV testing, including mobile testing of men from their places of work, as well as HIVST strategies has shown great potential to increase HIV uptake among men in sub-Saharan Africa [[54](#_ENREF_54)]. Studies have indicated that most men preferred using community-based HIV testing services, as compared to facility-based testing [[7](#_ENREF_7), [50](#_ENREF_50), [51](#_ENREF_51), [54](#_ENREF_54), [81](#_ENREF_81), [82](#_ENREF_82)]. Community-based HIV testing was also reported to have a significant effect on reaching a high number of clients who tested for HIV [[81](#_ENREF_81)]. In addition to the reduced stigma associated with community-based HIV testing services, the strategy also reduces the financial costs associated with travelling to facilities and improves access to HIV testing services among men of lower socio-economic status as well as reaching younger men [[83](#_ENREF_83), [84](#_ENREF_84)]. For instance, studies reported that men are more open to HIV counselling testing when it is conducted at home [[50](#_ENREF_50), [54](#_ENREF_54), [83](#_ENREF_83)], due to fear of being stigmatized in clinic settings, as well as judgements by healthcare workers. Home-based testing not only improves men’s uptake to HIV testing, but also promotes couple’s testing, as well as HIV status disclosure among couples [[51](#_ENREF_51), [83](#_ENREF_83), [85](#_ENREF_85), [86](#_ENREF_86)]. Despite the robust evidence on the benefits to community HIV testing among men in sub-Saharan Africa, there remains a gap as far as linkage to regular HIV prevention and care services [[54](#_ENREF_54)]. Therefore, there is a critical need to have innovative community-based approaches to improve linkage to prevention and care services.

Barbershops offer informal, easily accessible (i.e., location and hours of operation), and collegial spaces where men feel safe to talk about personal health issues, and the majority use the same barber most of the time [[63](#_ENREF_63)]. A recent systematic review of implementation of barbershop based health promotion interventions for black men in the United States showed that barbershop based interventions that prioritize community engagement and intentional alignment to the gender and lived experiences result in satisfactory recruitment, retention, and health related changes among men [[87](#_ENREF_87)] . Specifically, health initiatives based in barbershops reduced HIV risk behaviors and increased consistent condom use among heterosexual men in the USA [[88](#_ENREF_88)]. However, there is limited evidence on the feasibility and acceptability of barbershop-based approaches on HIV risk reduction practices including the use of HIV self-testing among heterosexual men in resource-limited settings [[89](#_ENREF_89)]. Proven HIV prevention strategies and tools may be incorporated in the barbershop-based services including peer-led counseling and education and HIV self-testing. In our preliminary assessments of the acceptability of the intervention components, we interacted with 20 barbers at a main island of Kalangala district (Bugala Island), all of whom welcomed the proposed initiative and were ready to introduce it to their customers. The barbers were also positive about the intervention being welcomed by their customers. To this end, we reached out to some customers who were receiving haircuts during the assessment; and they also welcomed the initiative and emphasized the need for accessible HIV prevention services in their community.

Leveraging the strong connections and frequent interactions that barbers have with men, this study will use the Capability-Opportunity-Motivation, and-Behavior (COM-B) model to design a barbershop-based HIV prevention initiative for heterosexual men at risk of HIV in Kalangala Islands, Uganda. The initiative will comprise of 1) barber-led HIV risk reduction counseling and education, 2) distribution of HIVST at barbershops, and 3) barber-led peer support groups.

## Rationale

The purpose of this study is to develop and assess the feasibility, acceptability, and preliminary effectiveness of a barbershop-based HIV prevention initiative among persons who identify as a heterosexual male and are behaviorally vulnerable to HIV. This research is important because innovative strategies to reach heterosexual African men vulnerable to HIV are urgently needed to end the AIDS epidemic. This study is focused on men in the Kalangala Islands in Uganda, a district with the highest HIV prevalence and incidence in the country [[30](#_ENREF_30), [31](#_ENREF_31)]. The study will leverage trusted relationships between men and barbers to deliver an HIV prevention initiative.

The HIV prevention initiative will center around barber-led activities: 1) barber-led HIV risk reduction education, 2) barbershop distributed HIVST kits, and 3) barber-led support group discussions and education. Barbershops will be randomized to be an intervention or control shop. Intervention shops will have barbers trained by the study to recruit men for data collection and deliver the HIV prevention initiative. Shops that are participating as controls will have barbers trained by the study to recruit men for enrollment into the data collection portion of the study but will not deliver any of the HIV prevention initiative components. All men eligible and enrolled in the study will be followed to understand the feasibility and acceptability of the initiative, and its impact on HIV testing, behaviors associated with HIV incidence, STI incidence, and linkage to prevention services (e.g. PrEP, PEP [post-exposure prophylaxis], Voluntary Medical Male Circumcision [VMMC]).

The intervention is based on the Capability, Opportunity or Motivational components of the COM-B Model of behaviour change [[90](#_ENREF_90)] . Capability refers to the ability of an individual to psychologically or physically engage in an activity; opportunity refers to the factors outside an individual that will influence someone’s behavior while motivation are the thought processes that direct a person to carry out specific behavior, as shown in Figure 2. [[90](#_ENREF_90)] The COM-B model postulates that changing the behavior of an individual, a group or a population needs changing either their capability, opportunity, and/or motivation needed to perform the behavior.[[91](#_ENREF_91)] Therefore, in this study we will use the COM-B model to provide a barbershop based HIV prevention initiative to men living in a high incidence setting and who are behaviorally vulnerable to HIV. The initiative will aim to increase men’s capability and motivation through the provision of HIV education and prevention measures by their own barbers. The initiative will also improve the opportunity for the use of HIV prevention tools among men by availing HIVST kits at the barbershop so that they are easily accessible by barbershop going men.

Figure 2. Modified COM-B model for implementation of the barbershop-based initiative.


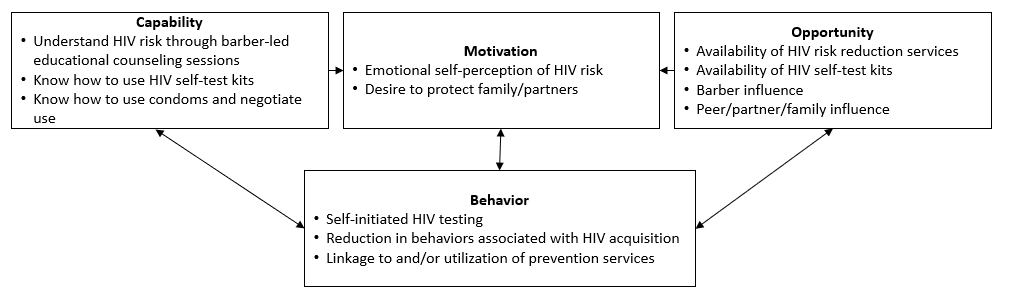


To ensure the successful global implementation of Long-Acting Pre-Exposure Prophylaxis (LAPrEP), it is essential to understand the perspectives and attitudes of heterosexual men behaviorally vulnerable to HIV, particularly in regions with high HIV incidence. Assessing the acceptability of LAPrEP among heterosexual men behaviorally vulnerable to HIV, such as those frequenting barbershops, will be crucial for informing the rollout of LAPrEP in these populations.

Overall, this study will contribute to the HPTN specific-aim of designing and conducting integrated strategies for HIV prevention, specifically using a diverse study design (cluster-randomized trial) to address HIV prevention needs of a priority, often-overlooked, population vulnerable to HIV. This study will provide valuable information about the feasibility of conducting the barbershop-based HIV prevention initiative and the potential for scale-up in other settings where barber-client relationships are an important component of men’s social structure. Understanding the fidelity to delivering the intervention will be an important component of scaling up this study into a larger trial. Fidelity will be assessed by understanding the adherence to the intervention, facilitation strategies used, quality of delivery, and participant responsiveness[[92](#_ENREF_92)]. Furthermore, the findings of this study will provide a crucial foundation for future research, including trials examining the use of LAPrEP in heterosexual men. Results will also inform the development of targeted interventions aimed at promoting LAPrEP uptake among heterosexual men, guide healthcare provider training and support, and facilitate the successful implementation of LAPrEP in this population.

# Study Objectives

The primary and secondary objectives of the study are listed below. Endpoints related to each objective are described in Section 8.1.

## Primary Objective

The primary objective of this study is:

1. To evaluate the feasibility and acceptability of a barbershop based HIV prevention initiative

## Secondary Objectives

The secondary objectives of this study are:

1. To compare completion of self-initiated HIV testing between intervention and control groups
2. To evaluate the preliminary effectiveness of the intervention on change in behaviors associated with HIV acquisition
3. To compare interest in or use of HIV prevention services between intervention and control groups
4. To assess interest in long-acting PrEP among all participants by study arm.

## Exploratory Objective

The exploratory objective of this study is:

1. To evaluate the preliminary effectiveness of the intervention on incident STIs

# Study Design

This is a cluster randomized trial that will randomly assign barbershops, in Kalangala district, Uganda, 2:1 to receive a barbershop-based HIV prevention intervention or the standard-of-care for HIV prevention and linkage to services. Prior to randomization, the barbershops will be purposively selected according to their radius from other shops, client base, and willingness and ability to deliver the intervention, as detailed in Section 3.1.1. Study participants will be recruited by the barbers from participating barbershops. Men attending barbershops will be informed about the study during haircuts by the barber and those interested will be linked to study staff for screening, and those who meet all eligibility criteria will be enrolled in the study. Participants will be persons who identify as heterosexual men and are behaviorally vulnerable to HIV, as defined by recent sexual practices. Up to 250 men ≥ 16 years will be enrolled across the 18 barbershops in the district. Young men ages 16 and 17 years old will be able to participate in this study if they meet local guidelines for being considered an emancipated or mature minor, contributing important information about this age demographic and their HIV prevention needs. Each participating shop will recruit a minimum of 10 men and continue until the maximum sample size of 250 is reached across study arms or until the end of the six-month enrollment period.

The study is designed to evaluate the feasibility and acceptability of a barbershop-based HIV prevention intervention and effectiveness of engaging men in HIV prevention through barbershops. The study will also provide data on the preliminary effectiveness of the intervention on changes in HIV testing and linkage to prevention services. Self-reported sexual behaviors associated with HIV incidence and STI incidence rates will also be compared.

In the intervention group, participants will be recruited from barbershops. Barbers will notify clients of the study and link them to study staff who will arrange for a brief pre-screening assessment followed by an enrollment visit at the study clinic. Study staff will provide transport to the study clinic for enrollment and will aim to complete all enrollment visits on the day that they are referred by the barber. Once enrolled in the study, at their next haircut, participants in the intervention group will receive status-neutral HIV education directly from their barber, barber-distributed HIV self-test kits for use outside of study visits, and invitations to barber-led support groups every two months.

In the control group, participants will be recruited from barbershops. Barbers will notify clients of the study and link them to study staff who will arrange for a brief pre-screening assessment followed by an enrollment visit at the study clinic. Study staff will provide transport to the study clinic for enrollment and will aim to complete all enrollment visits on the day that they are referred by the barber. Once enrolled, participants in the control group will be referred by study staff to local health facilities for standard-of-care HIV counseling and testing and self-test kit provision.

All participants (intervention and control group) will complete a Week 26 and Week 52 follow-up visit at the study clinic following enrollment. Study visits will include bio-behavioral data collection, including information on HIV testing practices and sexual behaviors. All participants will receive HIV counseling and testing, PrEP/HIV care referrals, STI (syphilis, gonorrhea, chlamydia) testing and treatment, and will complete a questionnaire on LAPrEP use at week 52. Participants in the intervention group will also be asked about the acceptability of the barbershop-based intervention and a subset will participate in in-depth interviews.

Prior to study initiation, barbers in both groups will be trained on the study, participant confidentiality, and study recruitment requirements. Barbers in the intervention group will have further training on delivering HIV education and specifics of HIV self-testing procedures. Barbers will also participate in in-depth interviews, questionnaires, and counseling skills assessments, to understand the acceptability and feasibility of the barbershop-based intervention.

See Appendix I for a schedule of study visits and procedures.

## Participating Sites

Central coordination of the study will occur at the MU-JHU clinical research site in Kampala, Uganda.

The barbershops and study visits will occur in the Kalangala Islands, on a maximum of four islands. Shops will be selected first from the largest island, Bugala Island, in an effort to keep all shops on a single island. If additional shops are needed to reach 18 shops, an additional three islands may be used. Table 1 shows the potential villages/towns, located at least 5km apart, from which shops can be selected to participate in the study. Not all shops will be selected for participation, as they must meet inclusion criteria listed in Section 3.1.1, and so that there are replacement shops if needed, as described in Sections 4.3 and 8.3.

Table 1. Villages/towns, at least 5km apart, that have a barbershop potentially eligible for study inclusion.

| **Island** | **Village/town** | **Population [**[**93**](#_ENREF_93)**]** |
| --- | --- | --- |
| Bugala | Kalangala | 1835 |
|  | Buyiri | 379 |
|  | Bumangi | 466 |
|  | Mutambala | 773 |
|  | Kyagalanyi | 1558 |
|  | Kibale | 695 |
|  | Bwendero | 1259 |
|  | Kibanga | 704 |
|  | Bweza-Dajje | 704 |
|  | Kaazi-Malanga | 815 |
|  | Kasamba | 194 |
|  | Kasenyi | 580 |
|  | Bbanga/Kagolomo | 569 |
|  | Senero | 739 |
|  | Bbeta | 1230 |
|  | Kasekulo | 2114 |
|  | Nakatiba | 988 |
|  | Bugoma | 1311 |
| Kachunga | Kachungwa | 484 |
| Bushozi | Misonzi | 1215 |
| Kachanga | Kachanga | 1716 |

### Barbershops

The barbershops will be purposively selected on the Kalangala Islands. In order to be selected for this study, barbershops must meet the following criteria:

- Adequate space to deliver the intervention to clients (e.g. space to store HIV self-test kits, laminated counseling sheets, lockable cabinet to store de-identified checklists, etc.)
- Have existed as a shop for ≥ six months
- Have a customer base of ≥ 15 clients per week
- A radius of ≥ 5km maintained between other participating shops

Barbershops may have multiple barbers and for a shop to be eligible for participation, participating barbers within the shop must also meet the following criteria:

- Barber must have at least a primary level of education
- Barber must be willing and able to participate in the study, recruit participants, and deliver the intervention if their shop is randomized to the intervention group

### Study Clinic Site

Research study visits will occur at the island-based satellite study site (co-located at an existing health facility). The study site is centrally located so that participants recruited from any barbershop on the island(s) will be able to attend study visits. In some instances, if a participant is unable to attend a visit at the clinic, the visit may occur at an alternative private location agreed upon by the participant and study staff.

Further details about the barbershops and satellite study site location are listed in the SSP Manual.

## Study Duration

Study duration is approximately two years total. Accrual is anticipated to occur over three – six months, with individual participants being followed on study for up to 12 months (52 weeks).

# Study Population

## Participants

Up to 250 heterosexual people who identify as male will be included in this study. Participants will be selected for the study according to the criteria in Section 4.1.1 and 4.1.2. and the guidelines in Section 4.1.3. Participants will be recruited, screened, and enrolled as described in Section 5.0. Requirements related to participant retention and withdrawal from the study are described in Sections 4.1.5 and 4.3, respectively.

### Inclusion Criteria

Persons who identify as a heterosexual male and meet all of the following criteria are eligible for inclusion in this study:

1. Age ≥ 16 years
   1. Any participants 16-17 years old will be enrolled following the Uganda National Council for Science and Technology (UNCST) guidelines for mature and emancipated minors
2. Able and willing to provide informed consent
3. Behaviorally vulnerable to HIV, based on self-report of at least one of the following in the last three months:
   1. Had condomless sex with a person of unknown HIV status or a person living with HIV
   2. Had more than one sexual partner
4. HIV negative per Ugandan Ministry of Health guidelines and the SSP Manual
5. Is a regular customer at a participating barbershop as defined in the SPP Manual

### Exclusion Criteria

Persons who meet any of the following criteria will be excluded from this study:

1. Not planning to stay in the study catchment area in the next 12 months
2. Any other condition that in the opinion of the Investigator of Record (IoR)/designee, would preclude informed consent, make study participation unsafe, complicate interpretation of study outcome data, or otherwise interfere with achieving the study objectives.

Participants who report sex with other men will not be excluded from this study so long as they meet the inclusion and exclusion criteria listed above.

### Recruitment Process

Study staff will work with stakeholders from the communities surrounding participating barbershops to provide education about the study and the involvement of the shops. Community sensitization will build trust within the study area, facilitate successful recruitment of participants, and contribute to a clear understanding of the barbers’ role in the study.

Study staff will work closely with barbers in both the intervention and control groups to recruit and screen clients from participating barbershops. Barbers will be trained on how to provide information about the study and link men to study staff if the client is interested. Barbers will not disclose which group (intervention or control) to which they have been randomized during participant recruitment. Once a potential participant is linked to study staff, the staff will contact the potential participant and administer a pre-screening checklist, either in-person or on the phone. If the man remains eligible based on pre-screening, study staff will proceed with facilitating the potential participant to attend the study clinic on the same day for an enrollment visit. In most cases, study staff will pick-up the client from the barbershop and transport directly to the clinic for an enrollment visit.

Appropriate recruitment processes and messaging geared towards the local community will be utilized and detailed in site standard operating procedures (SOPs). Site-specific advertising materials will undergo approval by the site’s responsible Institutional Review Board (IRB)/Ethics Committee (EC) prior to use.

### Co-Enrollment Guidelines

In general, participants in this study will not be allowed to take part in other concurrent HIV or interventional research studies during their participation in the study. This is due in part to concerns about participant study burden and to avoid confounding in the interpretation of the study data. The Protocol Team should be consulted for any possible exceptions, including for observational studies.

### Participant Retention

Once a participant enrolls in this study, the study site will make every effort to retain them for the duration of their follow-up to minimize possible bias associated with loss-to-follow-up. The study team will aim to achieve ≥90% retention at the Week 26 and Week 52 study visits. Retention procedures will be detailed in an SOP and may include:

- Study staff will ensure recruitment of men who are willing to participate in the study.
- During the informed consent process, study staff will thoroughly explain the study visits schedule, procedural requirements, and the importance of each study group; these points will be re-emphasized at each study visit.
- Study staff will collect detailed participant locator information during the screening visit. Locator information will be actively reviewed and updated at each subsequent visit.
- Study participants will be contacted or reminded of study visits and procedures, using their preferred mechanism of contact.
- Study staff will cultivate a supportive relationship with the study participants, barbers, and community to ensure high retention. The use of barbers who are in close contact with men is expected to augment retention plans. The barbers may support reminding the participants about study visits and missed visits.
- The MU-JHU Community Team, in consultation with the Community Advisory Board (CAB), will lead community engagement activities, including regular communication with the study community at large to increase awareness about HIV/AIDS and explain the purpose and importance of conducting and involving participants in HIV prevention research to enhance retention.
- Participants will indicate whether they can be visited at home, work, or other community locations by the study team. During the informed consent process, study staff will explain to the participant the requirements to conduct off-site visits, including type and duration of the visit, and procedures to maintain the participant’s information in a confidential manner. Study staff will take precautions not to disclose the purpose of the visits. Participants will be requested to authorize off-site visits on the informed consent form. Participants are free to decline home/off-site visits, and this will not affect participation in the study.
- The research clinics/sites will be designed to provide participant-friendly services such as having short duration clinic visits and flexible hours.

## Barbers

Barbershops will be selected to participate in the study if they meet the criteria listed in Section 3.1.1. Barbers at these shops will be asked to sign a memorandum of understanding (MOU) with the study regarding their role in recruiting participants and delivering the intervention if their shop is randomized to the intervention group. The MOU will also detail expectations in communication between the study team and the shop, particularly in case of any changes with study involvement. Barbers must also be willing and able to provide informed consent for research data collection (e.g. questionnaires and in-depth interviews) and be considered research participants (see Section 6.0). If a barbershop has more than one barber providing services, at least one barber at the shop will be asked to participate in client recruitment, delivering the intervention, and research procedures. Barbers are not required to be men.

## Participant Withdrawal

Regardless of the participant retention methods, participants (barbers or participants) may voluntarily withdraw from the study for any reason at any time. The IoR/designee may also withdraw participants from the study to protect their safety, if they are unwilling or unable to comply with required study procedures, and/or for other reasons, after consultation with the Protocol Chair, DAIDS Medical Officer, SDMC Protocol Statistician, and Leadership and Operations Center (LOC) Clinical Research Manager (CRM).

Participants also may be withdrawn if the study sponsor, government or regulatory authorities, or site Institutional Review Boards/Ethics Committees (IRBs/Ecs) terminate the study prior to its planned end date.

Every reasonable effort will be made to complete a final study visit (as described in Section 5.3) for participants who terminate from the study prior to their end of follow-up period (52 weeks), and study staff will record the reason(s) for all withdrawals from the study in participants’ study records.

If a barber or barbershop withdraws from the study, the Protocol Team will determine if replacement of the barbershop is feasible.

# Study Procedures for Participants

An overview of the study visits and procedures is presented in Appendix I. Additional information on specific study procedures is provided below. Detailed instructions to guide and standardize study procedures will be provided in the SSP Manual.

## Pre-Screening Assessment

Men who are interested in the study following barber-led recruitment will complete a pre-screening assessment (checklist) with the study staff. This pre-screening may occur in-person or over the phone and identifiable data will not be recorded during the pre-screening process. Study staff will aim to pre-screen potential participants in-person at or near the barbershop from where they are recruited; study staff will find a private space to administer the pre-screening assessment. If the participant is pre-screened over the phone, study staff will confirm that the potential participant is in a place they feel comfortable answering questions over the phone. During the pre-screening, potential participants will be assessed for sexual behaviors associated with HIV, their stay in the study catchment area, barbershop going status, and age.

If the potential participant remains eligible following this pre-screening assessment, they will proceed to the study clinic for an enrollment visit where all eligibility criteria will be assessed.

## Screening and Enrollment Visit

If a participant remains eligible and interested in study participation after pre-screening, they will complete full screening and enrollment procedures at the study clinic. The study team will aim to have all potential participants screen and enroll on the same day as the referral from the barber. For each participant, independent written informed consent will be obtained before any study related procedures are initiated.

*Screening*

A single informed consent form will be used for screening and enrollment into the study. To confirm eligibility criteria for the study, study staff will:

- Obtain written informed consent
- Collect basic locator and demographic information
- Collect socio-behavioral information
- Draw blood for HIV and syphilis testing
- Complete HIV testing and counseling according to Uganda Ministry of Health testing guidelines (provider-initiated HIV testing and counseling) as described in the SSP Manual.
  - If any result or interpretation other than negative or non-reactive is obtained for any HIV test, the person is not eligible for the study. Additional testing to confirm suspected HIV infection will be performed in accordance with local guidelines. If HIV infection is confirmed, participants will receive counseling and be referred for appropriate care, as necessary.

Participants who remain eligible after screening procedures will immediately proceed to enrollment. If the participant does not enroll within 14 days of the start of the screening visit, the participant can be re-screened up to two times at the discretion of the IoR/designee.

All participants will be offered condoms. Individuals deemed not eligible will be informed that they do not meet the eligibility criteria for the study, and referred for appropriate medical care, including HIV prevention services or HIV care, based on their needs.

*Enrollment*

An individual will be considered enrolled in the study once delegated study staff have completed an enrollment checklist that confirms all eligibility criteria. Once the participant is enrolled, study staff will:

- Collect full locator information and confirm any demographic information, if needed
- Collect additional socio-behavioral information. Audio computer assisted self-interview (ACASI) may be used to collect some self-reported measures.
- Collect information about medical history and concomitant medications
- Complete a symptom-directed physical exam, if clinically indicated.
- Ask participant to provide urine for gonorrhea and chlamydia testing.
- Disclose which group a participant’s barber was randomized to, so that the participant will know whether they will receive the intervention from their barber.
- Ask about initial feelings about the acceptability of the barbershop-based intervention.
- Counsel participants on the recommended HIV testing schedule for people behaviorally vulnerable to HIV: complete HIV testing quarterly, i.e. at three and nine months after study enrollment (participants will also have in-clinic HIV testing completed at their Enrollment, Week 26 & 52 study visits).
  - All participants will be asked to report on HIV testing behaviors between visits by contacting study staff when they test, using a toll-free study number. Participants will also be asked to send a picture of the used test kit or bring the used kit to the study clinic, if possible.
  - Participants in the intervention group will be reminded that they can access HIVST kits from their barber and participants in the control group will be provided with information about accessing standard-of-care testing.
- Refer participant for additional medical care if clinically indicated, including HIV prevention services.
- Offer condoms

Once a participant recruited from an intervention shop is enrolled, the study team will notify the participant’s recruiting barber to ensure that the individual receives the barbershop-based intervention at their next regular visit to the barbershop. Study staff will provide the barber with the client’s name but will not provide any study-related data to the barber. Enrolled participants will also be provided with a small study card. For participants in the intervention group, the card can be shown to the barber at their next haircut to indicate they should receive the intervention at the barbershop.

## Follow-Up Study Visits (Week 26 & 52)

Participants will be followed for up to 52 weeks, with visits scheduled at Week 26 and Week 52 after Enrollment.

At each follow-up visit, study staff will:

- Update locator information
- Collect self-reported socio-behavioral information. ACASI may be used to collect some self-reported measures.
- Collect information on HIV testing behaviors between visits
- Draw blood for HIV testing and storage for local laboratory (LL) quality control (QC)
- Complete HIV testing and counseling according to Uganda Ministry of Health testing guidelines (provider-initiated HIV testing and counseling) as described in the SSP Manual.
  - All participants will be reminded of the recommendation to complete HIV testing on a quarterly basis, consistent with Ugandan guidelines for persons behaviorally vulnerable to HIV.
- Ask participant to provide urine for gonorrhea and chlamydia testing.
- Collect information about medical history and concomitant medications
- Ask participant about social impacts of study participation
- Complete a symptom-directed physical exam, if clinically indicated, including if they report having a VMMC since the last visit.
- Refer participant for additional medical care, if clinically indicated, including HIV prevention or HIV care services.
- Offer condoms
- Collect information on interest in LAPrEP at week 52

A subset of participants will also be asked to participate in in-depth interviews (see Section 5.9)

## Procedures for Participants with Suspected or Confirmed HIV Infection

The Protocol Team must be notified of any reactive or positive HIV test result obtained during follow-up. Participants who have a confirmed reactive/positive HIV test result during study follow-up visits will have further testing to confirm infection as described in the SSP Manual. Participants who have a reactive/positive HIV result during follow-up will be referred for care and will continue their participation in the study.

If a participant has a reactive/positive test between study visits (e.g. using the HIV self-test kits), they will be counseled to come to the study clinic visit for confirmatory testing and follow-up. Participants will be able to contact study staff on a toll-free number to discuss HIV test results and linkage to care, as needed, between study clinic visits.

Participants will be encouraged to continue to attend study visits and receive HIV risk reduction counseling (e.g., treatment as prevention), STI testing, and complete socio-behavioral questionnaires. Study staff will collect information on linkage to HIV care and start of treatment. Participants will continue with their usual barber services and will not be required to disclose their HIV status to their barber. If the participant prefers not to receive any further HIV education from the barber or attend the group sessions, their decision will be respected. Study staff may notify the barber about the participant’s decision regarding the HIV prevention initiative provided by the barber, only if desired by the participant.

## Sexually Transmitted Infections (STIs)

Individuals will be tested for syphilis, gonorrhea, and chlamydia during the study per the schedule in Appendix I. Participants will be contacted if they have a positive test result and provided treatment at the study clinic following local guidelines for STI treatment and management. If a participant reports symptoms of an STI to study staff during the clinic visit, they will provide an evaluation, testing, and treatment as clinically indicated, according to the local and syndromic STI management guidelines; referrals for treatment may also be provided if needed. STI results, symptoms, and treatment provision/referrals will be recorded on study CRFs. There are no local health guidelines that require reporting of communicable diseases in Uganda and thus the site will not be required to report HIV or STI results to local authorities.

## Questionnaires Data Collection

Participants will complete questionnaires at study visits according to the schedule in Appendix I. Questionnaires/study CRFs will include the following key topics:

- Demographics
- Sexual behavior and other social/health practices associated with HIV acquisition
- Medical history, symptoms assessment, and medication use
- Utilization of HIV/STI testing, use of HIV prevention services (PrEP, PEP, VMMC), and HIV treatment services
- Attendance at barbershops and receipt of barbershop-based interventions, in both groups, including assessment of mobility between shops, frequency of visits, and components of the intervention received.
- Social impacts of study participation and barbershop-based interventions
- Acceptability of barbershop-based interventions
- Interest in long-acting PrEP

## Interim Contacts and Visits

Interim study contacts and study visits (those between regularly scheduled follow-up study visits) may be performed at participant request or as deemed necessary by the IoR/designee at any time during the study. All interim contacts and visits will be documented in participants’ study records and on applicable CRFs. Some interim visits may occur for administrative reasons. For example, the participant may have questions for study staff. Interim visits at which no data are collected are not documented on CRFs. If a participant contacts study staff with HIV test results, self-reported results will be recorded on study CRFs and the participant will be asked to come to the study clinic for follow-up testing and/or referral to care as needed.

## Barbershop-Based Intervention Procedures

Participants will continue attending the respective barbershop from where they were recruited on a schedule that is consistent with their regular barber services. Men may attend their barber at different intervals and this information will be documented as described in Section (6.5). Barbershop visits will not be considered formal study visits.

*Intervention group*

Study staff will notify barbers in intervention shops when one of their clients has been enrolled in the study so they are aware of who will receive the intervention. Participants will also be counseled by study staff to bring their study card to show the barber so they may deliver the intervention during the haircut. During the barber-participant interaction, barbers will provide general, status-neutral HIV education to the participant during their routine services and shops will have stock of HIVST kits available. Participants will be counseled to complete quarterly HIV testing, consistent with guidelines for persons behaviorally vulnerable to HIV.

Barbers will also provide invitations to barber-led support groups every two months. These support groups will take place at a location convenient to both the barber and participants (e.g. at the barbershop, local clinic, etc.).

If a participant in the intervention group permanently relocates to a different area on the Kalangala Islands where there is an intervention barbershop participating in the study, the participant may continue to receive the intervention from this barber, which will be facilitated by study staff, and the transfer of shops will be recorded in study data.

*Control group*

Barbers in the control group will not be notified by study staff about specific clients enrolled in the study. However, study staff will communicate total number of clients enrolled to provide feedback about recruitment targets. Participants in the control group will not receive any HIV prevention services from the barber but will continue with research study visits.

## Qualitative Data Collection

A subset of participants will be recruited to participate in individual in-depth interviews. Approximately 25 participants from the intervention group will be interviewed at Weeks 26 and/or 52. Participants will be purposively selected based on age, behaviors that make a person vulnerable to HIV, HIV prevention behaviors, experiences/challenges faced during the study, which barbershop they attend, or any other reason at the discretion of the investigator. Interviews will focus on the acceptability of the barbershop-based HIV prevention initiative and use of HIV prevention strategies. Interviewers will be trained in qualitative techniques, and they will follow a semi-structured guide with the ability to prompt participants based on the discussion content.

In-depth interviews will occur in a private space convenient for the participant and the study staff. Interviews will be digitally-recorded, transcribed, and translated into English if needed (see Section 8.6.4). Audio recordings will be stored until the conclusion of the primary qualitative data analysis. Details of the qualitative component with be outlined in the SSP Manual.

## Study Visit Windows

For each required study visit, there is an allowable visit window specifying when a post-enrollment visit should be completed. The allowable visit windows are contiguous between Week 26 and Week 52 and do not overlap. Within each allowable visit window, there is a target visit window when the visit should ideally be completed. Visit windows are outlined in Table 2 below. If more than one visit is necessary to complete all visit procedures, these can be completed during multiple days within the allowable visit window. Visit coding is described in the SSP Manual.

*Table 2. Study Visit Windows*

| **Visit** | **Target Visit Day** | **Target Visit Window** | **Allowable Visit Window** |
| --- | --- | --- | --- |
| Screening | Up to 14 days before enrollment | | |
| Enrollment | Day 0 | - | - |
| Week 26 | Day 182 | Day 168-196 | Day 154-336 |
| Week 52 | Day 365 | Day 351-379 | Day 337-427 |

# Study Procedures for Barbers

An overview of the study visits and procedures for barbers is presented in Appendix I. Additional information on specific procedures is provided below. Detailed instructions to guide and standardize study procedures will be provided in the SSP Manual.

## Barber Study Initiation

All barbers eligible to participate in the study (described in Sections 3.1.1 and 4.2) will be asked to sign an MOU to fully detail expectations of study staff and barbers before randomization and commencing training. Basic information about each participating shop will be recorded (e.g. total number of clients, hours of business, cost of services, etc.) by study staff and barber locator information will be collected.

Barbers will also provide informed consent for research-related data collection prior to undergoing any research study procedures.

## Barber Training

Prior to study activation for enrollment of participants, all barbers will undergo 1-2 days of training on the following topics:

- Study overview
- Participant confidentiality
- Expectations of barber in both control and intervention groups
- Recruitment processes and expectations

Barbers from the shops randomized to the intervention group will complete additional training on the following topics:

- HIV education content
- Counseling skills
- HIVST procedures
- Referral services available for HIV prevention and treatment
- Peer group structure and content
- Leading group sessions and protection of confidentiality in groups
- Expectations for recording information
- Support from study staff

Wherever possible, training content will be based on or adapted from existing materials from the Uganda Ministry of Health or other implementing partners. Training for intervention group barbers will take place over the course of approximately one week. A pre- and post- training assessment will be completed, and training content and length may be adapted based on these assessments. Training will be interactive and include role-play for barbers to gain experience with delivering the barbershop-based intervention and leading group sessions.

During the course of the study, study staff will provide support to barbers and check on their knowledge and confidence in delivery of the intervention. If knowledge gaps or more support is needed, the barbers will have opportunities for refresher training and cross-sharing with other barbers delivering the intervention.

A full curriculum for the barber training is included in the SSP.

## Questionnaires Data Collection for Barbers

Basic demographic information about each participating barber will be recorded on study CRFs. All barbers will be asked to complete brief questionnaires about their shop and recruitment experiences during the participant recruitment phase. Barbers in the intervention group will also be asked to complete brief questionnaires approximately quarterly after they begin delivering the intervention. Quarterly questionnaires will include information about the acceptability and feasibility of delivering the intervention. All barbers will also be asked about any social impacts of recruiting clients for the study and/or delivering the intervention.

## Qualitative Data Collection for Barbers

All barbers in the intervention group will be asked to participate in individual in-depth interviews approximately 6 and 12 months after starting delivery of the intervention. The interviews will be conducted to assess feasibility, acceptability, facilitators and barriers, and experiences of delivering the intervention. Interviewers will be trained in qualitative techniques, and they will follow a semi-structured guide with the ability to prompt participants based on the discussion content.

In-depth interviews will occur in a private space convenient for the participant and the study staff. Interviews will be recorded, transcribed, and translated into English if needed. Details of the qualitative component with be outlined in the SSP Manual.

## Barber Delivery of the Intervention

*Documentation requirements*

Barbers in the intervention and control groups will be asked to complete a simple, anonymous checklist on the number of men contacted and linked to study staff for screening.

Barbers in the intervention group will further be asked to complete a simple checklist of the services provided to each client enrolled in the study. The checklist will not contain any direct identifiers and will indicate if HIV education was provided to the client, number of HIVST kits distributed, and if referral information was given. The checklist will be stored in a lockable cabinet at the shop and collected once weekly by study staff.

*Status-neutral HIV education*

Barbers in the intervention group will provide status-neutral education to all enrolled participants while providing haircut services. A laminated counseling guide will be provided to the barber so that they may follow set messages for the education. If there are clients waiting while an enrolled participant is receiving their services, the barber will be trained to provide the education to all persons inside the shop at the time.

*HIV self-test kits*

Barbers in the intervention group will have a stock of study provided HIVST kits within the shop that can be provided to the participant to take home. The barber will be trained to provide counseling on how to complete the test and the recommended frequency for testing (i.e., every 3 months for men behaviorally vulnerable to HIV). Barbershops will also have opaque bags for participants to discretely carry the HIVST kits out of the shop. If a client who is not enrolled in the study is there during HIV education of the participant and would like to take an HIV self-test kit, they will be able to do so.

*Referral information for HIV services*

Barbers in the intervention group will be knowledgeable about existing services for HIV prevention within the Kalangala district. These services will include HIV counseling and testing, free condoms, PrEP and PEP provision, and VMMC. They will also provide information about HIV treatment and the health and prevention benefits.

*Peer groups*

Barbers in the intervention group will facilitate peer support groups every two months that will focus on HIV education and available peer support. The groups will allow for peer support and shared experience between men. Study staff will assist with coordinating the peer groups, recording the number of men in attendance, documenting the HIV education topics covered, and the quality/accuracy of the delivery of information. Men’s attendance at the peer group sessions will be recorded in the database.

# Safety Monitoring

## Safety Monitoring

This protocol involves minimal-risk procedures (recruitment, testing for HIV/STIs, questionnaires, support for linkage to care, HIVST provision) and contains no biomedical intervention or clinical care (e.g. PrEP/ART prescription); thus, standard adverse event reporting is not required. Adverse events will be reported to the local IRB as required per their guidelines. The study team will collect and report all social impacts/harms that are brought to the attention of study staff. Study participants will be provided a 24-hour telephone number and contact information and instructed to contact the study staff to report any concerns or social impacts they may experience. The study staff will also be available via telephone for consultation on HIV test results if testing is done outside the study clinic. For life-threatening events, participants will be instructed to seek immediate emergency care.

## Social Impact Reporting

It is possible that participants' or barbers’ involvement in the study could become known to others, and that a social impact may result (i.e., because participants could be perceived as having HIV or at increased likelihood for HIV acquisition). For example, participants could be treated unfairly, or could have problems being accepted by their families and/or communities. Barbers may lose clients if they learn about their involvement in the study. A social impact that is reported by the participant/barber and judged by the IoR/designee to be serious or unexpected will be reported to the responsible site’s IRBs at least annually, or according to the IRB requirements. Participants and barbers will be advised to contact study staff at any time if they feel they have experienced a social impact due to study participation. Social impacts will be collected and reported on CRFs during regular visits for participants and barbers. In the event that a participant or barber reports a social impact, every effort will be made by study staff to provide appropriate care and counseling to the participant as necessary, and/or referral to appropriate resources and services for the safety of the participant. The site will provide such care and counseling in accordance with standardized guidance in the SSP Manual. While maintaining participant confidentiality, study sites may engage their CAB in exploring the social context surrounding instances of social impacts, to minimize the potential occurrence of such an impact.

## Study Monitoring Committee Review

The SDMC will prepare routine study conduct reports for the Study Monitoring Committee (SMC), which will meet by conference call approximately every six months during study implementation and will review accrual, retention, intervention implementation, data completeness and social impact data during a closed meeting. More frequent or *ad hoc* reviews of study conduct/safety reports may be conducted by the SMC as needed. A recommendation to stop the trial may be made by the SMC at any time that the team agrees the study implementation is futile.

Reports of social impacts and subsequent actions taken will be reviewed at least every six months, if indicated, and reported to the DAIDS MO. Social impacts will be reported to local IRB/ECs as required.

# Statistical Considerations

## Objectives and Endpoints

### Primary Objective and Endpoints

#### To evaluate the feasibility and acceptability of a barbershop based HIV prevention initiative

Consistent with this primary study objective, the following endpoint(s) will be assessed from participants in the intervention group:

- Participant responses from surveys at Week 26 and Week 52 about acceptability of the intervention
- Barber responses from surveys about feasibility and acceptability of intervention
- Recruitment rates during enrollment and retention in study and barbershop activities at Week 26 and Week 52
- Frequency of barber-participant interactions and proportion that include delivery of the intervention

### Secondary Objectives and Endpoints

#### To compare completion of self-initiated HIV testing between intervention and control groups.

Consistent with this primary study objective, the following endpoint(s) will be assessed:

Self-report of HIV test conducted during the study follow-up.

#### To evaluate the preliminary effectiveness of the intervention on change in risk behaviors associated with HIV acquisition

Consistent with this secondary study objective, the following endpoint(s) will be assessed:

- Self-reported risk behaviors during the study follow-up, including condom use and number of total and new sexual partners.

#### To compare interest in or use of HIV prevention services between intervention and control groups

Consistent with this secondary study objective, the following endpoints will be assessed:

- - - - VMMC during the study
      - Self-reported access to PEP care or use of PEP
      - Self-reported access to PrEP care or use of any PrEP method during the study

#### To assess the preliminary interest in LAPrEP among all participants

Consistent with this secondary study objective, the following endpoints will be assessed:

- - - - Self-reported interest in LAPrEP at Week 52

### Exploratory Objective and Endpoints

#### To evaluate the preliminary effectiveness of the intervention on incident STIs

Consistent with this exploratory study objective, the following endpoint(s) will be assessed:

- Incidence of HIV between screening and Week 52
- Incidence of gonorrhea and chlamydia between screening enrollment and Week 52

## Sample Size

Sample size is based on the secondary objective of self-initiated HIV testing since the primary objective pertains only to the intervention arm. For self-initiated testing between enrollment and 26 weeks (or equivalently between 26 weeks and 52 weeks), power is calculated accounting for the cluster randomization and 2:1 randomization [[94](#_ENREF_94)]. We provide power for 18 barbershops recruiting between 10 and 14 participants each. The assumed between-cluster coefficient of variation of the true proportions are the same in each arm and equal to 0.25. If retention targets are met, loss to follow-up will be 10% for responses to questions about HIV testing on the 26- and 52-week questionnaires. We are interested in detecting an absolute difference in testing rates between arms of 20%.

Table 3 shows power to detect an absolute increase of 20% in HIV testing rates in the intervention arm compared to the control arm. Assuming self-initiated testing occurs in only 5% of men in the control arm, we have at least 80% power to detect an increase of 20% (5% control vs. 25% intervention) with 10 men per barbershop and 18 barbershops. Recruiting at least 10 men per shop with 10% loss to follow-up results in high (81%) power for detecting a difference of 10% vs. 30%. If approximately the maximum number of men are recruited and evenly distributed among shops (14 men per shop), we have at least 80% power to detect a difference of 15% in the control arm vs. 35% in the intervention arm, with 10% loss to follow-up.

*Table 3: Power to detect a risk difference of 20% between arms for any self-initiated HIV testing (between enrollment and Week 26 visits) with 2:1 randomization of 18 barbershops and a between-cluster coefficient of variation of 0.25*

| **Proportion testing in control arm** | **Power (10 enrolled per shop)** | **Power (12 enrolled per shop)** | **Power (14 enrolled per shop)** |
| --- | --- | --- | --- |
| 10% loss to follow-up | | | |
| 5% | 92% | 95% | 97% |
| 10% | 81% | 86% | 90% |
| 15% | 70% | 77% | 82% |
| 20% loss to follow-up | | | |
| 5% | 89% | 93% | 96% |
| 10% | 76% | 83% | 87% |
| 15% | 66% | 73% | 78% |

## Accrual and Retention

The enrollment target for each barbershop is at least 10 participants (a minimum of 180 total men). Barbershops will be allowed to recruit as many participants as they can in the enrollment period, up to 250 men, as funding allows. Accrual is expected to last three – six months and all participants will have 52 weeks of follow-up. Further details of the accrual period and enrollment targets are included in the SSP Manual.

If a barbershop is unable to recruit a minimum of 5 participants within the first three months of enrollment, recruitment will cease from this barbershop and the Protocol Team will determine if a replacement barbershop is required from within the same village/area to meet the study objectives. Replacement barbershops will be assessed using the same inclusion criteria, and assigned to the arm of the barbershop they are replacing. Additional details about barbershop replacement is in the SSP. If a barbershop is unable to recruit any participants within the first month of enrollment, study staff will contact the barber to investigate, and the Protocol Team will determine whether a replacement barbershop is needed. Enrollment caps for shops will be based on budget limitations.

The minimum target for retention is 90% for the Week 26 and Week 52 visits.

## Random Assignment/Study Arm Assignment

Barbershops will be randomly assigned to a study arm prior to implementation of training. Stratification or restricted randomization may be used to balance important factors between arms, e.g. distance from satellite study clinic or village size. 2:1 randomization will be used so that there are approximately twice as many shops in the treatment arm as the control arm; this is to allow more information to be gathered about the process of implementing the barbershop-based intervention and its feasibility and acceptability. Randomization procedures will be fully detailed in the SSP Manual prior to the randomization event.

## Blinding

Barbers, participants, and study staff will not be blinded to the intervention.

## Statistical Analysis

### Primary Analyses

Primary analyses will use appropriate methods for cluster-randomized trials or barbershop-level outcomes which are not clustered and will be analyzed using traditional statistical methods appropriate for independent data.

Survey questions about feasibility and acceptability will be summarized. No comparison will be made between arms for barber surveys or participant survey questions that are only applicable to the treatment arm. Questions about acceptability and participant experience that are applicable to both arms will be compared between arms using methods appropriate for the question type. Recruitment by each barbershop will be summarized by rates over time (number of men enrolled per week of recruitment) and rates over client visits (number of men enrolled per visit at which barbers discussed enrollment in the study).

Retention in study activities will be summarized by the percent of enrolled men attending the Week 26 and Week 52 visits. Retention and engagement in barbershop-based activities will be summarized by participant and barber reported engagement (receiving information from barbers at haircuts, attending peer groups, taking self-test kits from barbers). Retention and engagement of barbers will be assessed by barber surveys and reports on study activities. No formal comparison between arms will be made for the recruitment and retention metrics.

Frequency of barber-participant interactions in the intervention arm based on checklists from barbers and surveys of clients will be summarized overall and over time. Where available, descriptions of the interactions will be reported, e.g. proportion of haircuts at which client received counseling.

### Secondary Analyses

Formal comparisons of secondary endpoints will use appropriate methods for cluster-randomized trials for participant-level outcomes.

To assess self-initiated HIV testing, the proportion of participants reporting HIV testing will be compared between arms for the first portion of the study (between enrollment and the 6-month visit) and the second portion of the study (between the 26-week and 52-week visits). In addition, the proportion who reported HIV testing during both periods will be compared between arms. To estimate the difference in proportion of men testing, we will use a risk difference from a cluster-level analysis. Inference will be based on a t-test due to the small number of clusters; details on the testing procedure will be described in the statistical analysis plan.

To assess the effect on change of behaviors associated with HIV acquisition, self-reported behavior will be summarized by arm at all available time points. Behaviors will be compared between arms. To assess linkage to HIV prevention and care services, the proportion of participants who reported VMMC will be compared between arms, and the proportion of participants who reported use of any PEP or PrEP method will be compared between arms. To assess access toPrEP and/or PEP, the proportion of participants who report receiving a prescription for PrEP and/or PEP or attending a clinic to be screening for PrEP/PEP eligibility, will be compared between arms.

To assess the interest in long-acting PrEP (oral and injectable) at Week 52, responses to the long-acting PrEP interest questionnaire will be summarized and compared between arms.

### Exploratory Analyses

Incidence of HIV, gonorrhea, and chlamydia will be compared between arms, using appropriate methods for cluster-randomized trials.

### Qualitative Analyses

All qualitative interviews will be digitally audio recorded and transcribed by study staff, then translated into English, if necessary. Transcripts will be reviewed by the study team to identify any text that may be unclear or to identify areas in which interviewing or transcription techniques could be improved. Transcripts will be uploaded into a qualitative data management software for coding and analysis. Codebooks will be developed and modified iteratively based on emergent themes throughout the coding process. Any discrepancies between coders will be discussed and resolved by consensus. Topical codes will then be read across transcripts and grouped by emergent themes within each topic using a content analysis approach.

### Interim Stopping Plan

No formal stopping rules are in place, due to the short enrollment window and follow-up period of this study, and due to the minimal risk of the intervention to participants.

# Laboratory Specimens and Biohazard Containment

Laboratory procedures are described below and Section 5; additional tests to be performed at a subsequent visit for participants who have a reactive or positive HIV test at or after enrollment are described in the SSP Manual.

## Local Laboratory Specimens

As described in Section 5, the following types of specimens will be collected for testing at the local clinic and/or laboratory (LL):

- Blood
- Urine

As described in Section 5, the following types of testing will be performed at the local clinic or LL:

- HIV testing (see SSP manual)
- Testing for syphilis – Rapid Plasma Reagin (RPR) with TP Hem-Agglutination (TPHA) for confirmation
- Nucleic Acid Amplification Testing (NAAT)for Chlamydia trachomatis and Neisseria gonorrhea (CT/NG)

The local clinic or LL will perform HIV rapid testing. LLs will perform STI testing to include syphilis screening and CT/NG.

The study site must adhere to the HPTN Manual of Operations (MOP), the SSP Manual, and local SOPs, for proper collection, processing, labeling, transport, and storage of specimens to the LL. Non-US laboratories performing these tests will be monitored by an External Quality Assurance (EQA) provider or specified quality assurance contractor. In addition, the study site must adhere to the Requirements for Laboratories in Performing Testing for DAIDS-Supported and/or Sponsored Clinical Trials policy (DAIDS-OD-A-POL-00002) as outlined in the DAIDS SCORE Manual and the DAIDS Good Clinical Laboratory Practice (GCLP) as appropriate. Laboratories must also follow the DAIDS Clinical Research Laboratory and Specimens Management policies at <https://www.niaid.nih.gov/research/daids-clinical-research-laboratory-specimens-management>. Specimen collection and storage at the LL for QC will be documented as described in the SSP Manual.

### Specimen Storage

Blood samples will be stored to complete quality control for HIV testing during the study at the LL, as described in Section 9.3. Samples will be destroyed following completion of the testing and no samples will be stored for long-term storage.

## Quality Control and Quality Assurance Procedures

HPTN LC staff may conduct periodic visits to the site/LL to review the implementation of on-site laboratory quality control (QC) procedures, including proper maintenance of laboratory equipment and use of appropriate supplies and reagents. HPTN LC staff will follow up directly with site staff to resolve any QC or quality assurance (QA) problems identified through proficiency testing or on-site visit reviews.

## HIV Diagnostic Testing

The LL will perform testing for HIV diagnosis at Enrollment and other scheduled visits as described in the SSP Manual. HIV infection status will be confirmed at study sites using local HIV testing guidelines. At every scheduled clinic visit, 10% of the samples tested for HIV at the study clinic will be tested again/verified at an HPTN affiliated local laboratory for quality control purposes. In addition, if a participant has signs or symptoms consistent with acute HIV infection, or expresses a concern about recent HIV acquisition, testing will be performed using an HIV RNA test. Regardless of whether HIV RNA testing is used for diagnostic testing, HIV infection must be confirmed in all cases using two independent samples collected on different days. HIV Algorithms for HIV diagnostic testing are provided in the SSP Manual.

## Biohazard Containment

As the transmission of HIV and other blood-borne pathogens can occur through contact with used needles, blood, and blood products containing HIV, appropriate blood and secretion precautions will be employed by all personnel in the drawing of blood and shipping and handling of all specimens for this study, as currently recommended by the United States Centers for Disease Control and Prevention. All specimens will be shipped using packaging that meets requirements specified by the International Air Transport Association Dangerous Goods Regulations for UN 3373, Biological Substance, Category B, and Packing Instruction 650.

# Human Subjects Considerations

## Ethical Review

The HPTN Ethics Working Group developed the HPTN Ethics Guidance for Research, a network-wide ethical principles document, which is suitable for further elaboration and tailoring for each study.

This protocol and the template informed consent forms (ICFs) contained in Appendix II will be reviewed and approved by the HPTN Scientific Review Committee and NIAID Prevention Science Review Committee with respect to scientific content and compliance with applicable research and human subjects regulations.

The protocol, site-specific ICFs, participant education and recruitment materials, other requested documents, and any subsequent modifications will also be reviewed and approved by the ethical review bodies responsible for oversight of research conducted at the study site.

Subsequent to initial review and approval, the responsible IRBs/ECs will review the protocol at least annually. The Investigator will make safety and progress reports to the IRBs/ECs at least annually, and within three months of study termination or completion. These reports will include the total number of participants enrolled in the study, the number of participants who completed the study, all changes in the research activity, and all unanticipated problems involving risks to human subjects or others. The study site will submit documentation of continuing review to the DAIDS Protocol Registration Office (PRO), in accordance with the current DAIDS Protocol Registration Policy and Procedures Manual.

## Informed Consent

Written informed consent will be obtained from each study participant, including mature and emancipated minors, according to the local IRB and UNCST guidelines. Mature and emancipated minors are not required to have parental consent per these guidelines and attempting to have parental consent could complicate enrollment or breach confidentiality related to HIV or sexual health of these young men who otherwise would make meaningful contributions to the study objectives. The template study ICFs are included in Appendix II, which describes the purpose of the study, the procedures to be followed, and the risks and benefits of participation, in accordance with all applicable regulations. The study site will update the template with pertinent local information and is also responsible for translating the template form into local languages and verifying the accuracy of the translation by performing an independent back-translation.

Literate participants will document their provision of informed consent by signing their ICF. Non-literate participants will be asked to document their informed consent by marking their ICF (e.g., with an X, thumbprint, or other mark) in the presence of a literate third-party witness. Further details regarding DAIDS requirements for documenting the informed consent process with both literate and non-literate participants are provided in the DAIDS SCORE Manual. Any other local IRB/EC requirements for obtaining informed consent from non-literate persons also will be followed.

Participants will be provided with a copy of their ICF(s) if they are willing to receive them.

## Risks

*Participants*

Participants may become embarrassed, worried, or anxious when answering questions about their HIV/STI risk or when receiving HIV/STI counseling and testing. Trained counselors will be available to help the participant with these feelings.

Phlebotomy may lead to discomfort, feelings of dizziness or faintness, bruising, and/or swelling. In rare cases, phlebotomy can lead to infection.

Although the study site will make every effort to protect participant privacy and confidentiality, it is possible that a participants’ involvement in the study or other aspects of their identity could become known to others, and that social harms may result (i.e. because participants may be identified as at-risk for HIV/STIs). For example, participants could be treated unfairly or discriminated against, experience stigma, or have problems being accepted by their families or communities. It is possible that the participant’s barber may become aware of or assume a participant’s HIV status based on their participation in the study.

Any participant who experiences an adverse event related to study participation will receive follow-up by the study team. If management is required beyond the capacity of the study, the participant will be referred to appropriate services or organizations that can provide the required management.

*Barbers*

Although the study will ensure to make barbershop based procedures as neutral as possible to all the clients visiting the shop, it is possible that barbershop/barber’s involvement in the study or study procedures may be perceived or interpreted differently by others, and that social harms may result (i.e., their shop may be identified as a place associated with HIV/STIs). For example, barbers could be treated unfairly or discriminated against, experience stigma, or have problems being accepted by their families or communities. It is possible that the barber may have difficulty with retaining clients or getting new clients if people find out they are participating in the study or delivering the intervention.

## Benefits

*Participants*

There may be no direct benefit to participants in this study, however, participants and others may benefit in the future from information learned in this study.

Participants will receive HIV counseling and testing and will be linked to care, if needed. They will also receive STI testing and treatment.

*Barbers*

There may be no direct benefit to barbers in this study, however, as part of the community, barbers may benefit in the future from information learned in this study.

Barbers will receive HIV education including preventive strategies and may benefit from the information they learn.

## Incentives

*Participants*

In accordance with local IRB/EC approvals, participants will be compensated for their time and effort in this study, and/or be reimbursed for travel to study visits and time away from work. Reimbursement amounts will be specified in the study ICF.

*Barbers*

Barbers will receive compensation for their time recruiting participants, delivering the intervention and completing study documentation (e.g. tracking number of clients reached) and this will be detailed in the SSP Manual and specified in the MOU. Barbers will also be compensated for their time and effort for study procedures (in-depth interviews and study questionnaires) and/or reimbursed for travel for study visits and time away from work. Reimbursement amounts for research procedures will be specified in the study ICF.

## Confidentiality

All study-related information will be stored securely at the satellite clinic site and MU-JHU. All participant information will be stored in locked file cabinets in areas with access limited to study staff. Participant related documents at the barbershops will be anonymized and kept in a locked cabinet at the barbershop. All laboratory specimens, reports, study data collection, process, and administrative forms will be identified by a coded number only to maintain participant confidentiality. All local databases will be secured with password-protected access systems. Forms, lists, logbooks, appointment books, and any other listings that link participant ID numbers to other identifying information will be stored in a separate, locked file in an area with limited access.

Participant’s study information will not be released without the written permission of the participant, except as necessary for review by the NIAID and/or its contractors; representatives of the HPTN LOC, SDMC, and/or LC; other government and regulatory authorities, and/or site IRBs/ECs.

## Study Discontinuation

The study may be discontinued at any time by NIAID, the HPTN, and/or site IRBs/ECs.

# Administrative Procedures

## Protocol Registration

Initial Registration of the protocol by the DAIDS PRO is required prior to the implementation of this protocol. As part of this process, the study site must have the protocol and protocol ICF(s) approved, as appropriate, by their IRB/EC and any other applicable regulatory entity (RE). Upon receiving final approval, the site will submit all required protocol registration documents to the DAIDS PRO at the Regulatory Support Center (RSC). The DAIDS PRO will review the submitted protocol registration packet to ensure that all required documents have been received. In the case of Initial Registration, site-specific ICFs WILL be reviewed and approved by the DAIDS PRO. The site will receive an Initial Registration Notification from the DAIDS PRO that indicates successful completion of the protocol registration process. A copy of the Initial Registration Notification should be retained in the site's regulatory files.

Upon receiving final IRB/EC and any other applicable RE approval(s) for an amendment, sites should implement the amendment immediately. The site is required to submit an amendment registration packet to the DAIDS PRO at the RSC. The DAIDS PRO will review the submitted protocol registration packet to ensure that all the required documents have been received. Site-specific ICF(s) WILL NOT be reviewed and approved by the DAIDS PRO and sites will receive an Amendment Registration Notification when the DAIDS PRO receives a complete registration packet. A copy of the Amendment Registration Notification should be retained in the site’s regulatory files.

For additional information on the protocol registration process and specific documents required for initial and amendment registrations, refer to the current version of the DAIDS Protocol Registration Manual, which can be found at:

<https://www.niaid.nih.gov/sites/default/files/prmanual.pdf>.

## Study Activation

Pending successful protocol registration and submission of all required documents, the HPTN LOC staff will “activate” the site to begin study operations. Study implementation may not be initiated until a study activation notice is provided to the site by the HPTN LOC. In addition, if study activation is determined to be necessary for any subsequent amendments, study implementation may not be initiated until a study activation notice is provided to the site by the HPTN LOC.

## Study Coordination

Study implementation will be directed by this protocol as well as the SSP Manual and the DAIDS SCORE Manual.

Study CRFs and other study instruments will be developed by key members of the protocol team and HPTN SDMC. Data will be submitted to the HPTN SDMC for data cleaning, reporting and analysis. Data management and coding queries will be generated and applied to the data by HPTN SDMC staff on a routine basis for verification and resolution by site staff.

Close coordination between protocol team members will be necessary to track study progress, respond to queries about proper study implementation, and address other issues in a timely manner. Rates of accrual, adherence, follow-up, and visit completion rates will be monitored closely by the team as well as the HPTN SMC. The Protocol Team will address issues related to individual study eligibility, social harms, participant safety, consistent case management, documentation, and information-sharing across teams.

## Study Monitoring

The study is considered low risk by DAIDS and will not be monitored by DAIDS or any non-DAIDS entities. However, oversight will be provided for the implementation of the protocol, including, but not limited to:

- Routine protocol team monitoring of study operations
- HPTN SMC review (as described in Section 7.3)
- Periodic site visits from the HPTN LOC, HPTN LC, or DAIDS and/or their designees

Authorized representatives of the HPTN LOC, HPTN SDMC, HPTN LC, NIAID, Uganda Joint Clinical Research Center (JCRC) IRB, Uganda National Council for Science and Technology (UNCST), Uganda Ministry of Health (MOH), Uganda National Health Research Organization (UNHRO), and US regulatory authorities (Office for Human Research Protections (OHRP), will be allowed to inspect facilities and study documentation. A site visit log will be maintained at all study locations to document visits.

## Protocol Compliance

The study will be conducted in full compliance with the protocol. The protocol will not be amended without prior written approval by the Protocol Chairs and DAIDS Medical Officer. All protocol amendments must be submitted to and approved by the relevant IRB(s)/EC(s) and the RSC prior to implementing the amendment.

## Investigator’s Records

The Investigator will maintain, and store in a secure manner, complete, accurate, and current study records throughout the study. Under the US Department of Health and Human Services (DHHS) regulations, the Investigator is required to retain all study records relating to research for at least three [3] years after completion of the research, or longer if needed to comply with local regulations.

Completion of a clinical research study occurs when the following activities have been completed:

- All research-related interventions or interactions with human subjects (e.g., when all subjects are off study);
- All protocol-required data collection of identifiable private information described in the IRB/EC-approved research plan;
- All analysis of identifiable private information described in the IRB/EC-approved research plan;
- Primary analysis of either identifiable private or de-identified information.

Study records include administrative documentation including protocol registration documents and all reports and correspondence relating to the study; as well as documentation related to each participant screened and/or enrolled in the study, including ICFs, locator forms, CRFs, and notations of all contacts with the participant; and all other source documents. Direct data entry or direct data capture of study data into the study database is allowed when capturing information from the participant. Study data may also be sourced from electronic or paper source documents prior to being entered into the study database. For all study visits, it is recommended that data are entered into electronic Case Report Forms (CRFs) from paper or electronic source; however, there may be data generated for which there are no other source documents such as participant self-report. Site specific CRF source documentation tables found in the Study Management Overview SSP Manual (Section 3, pages 9-12) will specify site-specific source, for all CRF items. Sites will also document back-up, paper-based procedures, in the event that electronic systems are unavailable.

## Use of Information and Publications

Publication of the results of this study will be governed by the HPTN MOP. Any presentation, abstract, or manuscript will undergo review by the HPTN Manuscript Review Committee, and DAIDS prior to submission.

## ClinicalTrials.gov

This protocol is not an FDAAA “applicable clinical trial.” However, this study is subject to the NIH Policy on the Dissemination of NIH-Funded Clinical Trial Information. A description of this clinical trial will be available on [http://www.ClinicalTrials.gov](http://www.clinicaltrials.gov/).

References

1. UNAIDs, J.U.N.P.o.H.A., *Global AIDs update 2020*, in *Seizing the Moment*. 2020: 20 Avenue Appia, 1211 Geneva 27, Switzerland.

2. UNAIDS, t.J.U.N.P.o.H.A., *IN DANGER: UNAIDS Global AIDS Update 2022. Geneva: Joint United Nations Programme on HIV/AIDS; 2022. Licence: CC BY-NC-SA 3.0 IGO*. 2022.

3. UNAIDs, *Global AIDs update 2021: CONFRONTING INEQUALITIES; Lessons for pandemic responses from 40 years of AIDS*. 2021: UNAIDS Joint United Nations Programme on HIV/AIDS, 20 Avenue Appia 1211 Geneva 27 Switzerland.

4. Cornell, M., et al., *HIV services in sub-Saharan Africa: the greatest gap is men.* The Lancet, 2021. **397**(10290): p. 2130-2132.

5. Grimsrud, A., et al., *Shifting the narrative: from “the missing men” to “we are missing the men”.* Journal of the International AIDS Society, 2020. **23**(Suppl 2).

6. UNAIDs. *Male engagement in HIV testing, treatment and prevention in Eastern and Southern Africa: A framework for action*. 2022 [cited 2023 15 Mar 2023]; Available from: <https://reliefweb.int/report/world/male-engagement-hiv-testing-treatment-and-prevention-eastern-and-southern-africa>.

7. Sharma, M., R.V. Barnabas, and C. Celum, *Community-based strategies to strengthen men’s engagement in the HIV care cascade in sub-Saharan Africa.* PLoS medicine, 2017. **14**(4): p. e1002262.

8. Boily, M.C., et al., *Heterosexual risk of HIV-1 infection per sexual act: systematic review and meta-analysis of observational studies.* Lancet Infect Dis, 2009. **9**(2): p. 118-29.

9. De Oliveira, T., et al., *Transmission networks and risk of HIV infection in KwaZulu-Natal, South Africa: a community-wide phylogenetic study.* The lancet HIV, 2017. **4**(1): p. e41-e50.

10. Colvin, C.J., *Strategies for engaging men in HIV services.* The Lancet HIV, 2019. **6**(3): p. e191-e200.

11. Dovel, K., et al., *Gendered health institutions: examining the organization of health services and men’s use of HIV testing in Malawi.* Journal of the International AIDS Society, 2020. **23**: p. e25517.

12. Katz, D.A., et al., *Male perspectives on incorporating men into antenatal HIV counseling and testing.* PLoS One, 2009. **4**(11): p. e7602.

13. Larsson, E.C., et al., *Mistrust in marriage--reasons why men do not accept couple HIV testing during antenatal care- a qualitative study in eastern Uganda.* BMC Public Health, 2010. **10**: p. 769.

14. Betron, M., et al., *Men, masculinities and HIV/AIDS: Strategies for Action.* International Center for Research on Women, Instituto Promundo, MenEngage Alliance, and Sonke Gender Justice Network, 2012.

15. Dworkin, S.L., S. Treves-Kagan, and S.A. Lippman, *Gender-transformative interventions to reduce HIV risks and violence with heterosexually-active men: a review of the global evidence.* AIDS and Behavior, 2013. **17**: p. 2845-2863.

16. Fund, U.N.P., *State of world population 2021: my body is my own. PDF*. 2021: New York.

17. Federation, I.P.P., *Gender equality strategy and implementation plan. PDF*. 2017: London.

18. Ramadanovic, B., et al., *Changing risk behaviours and the HIV epidemic: a mathematical analysis in the context of treatment as prevention.* PLoS One, 2013. **8**(5): p. e62321.

19. HIV/AIDS, J.U.N.P.o., *Global report: UNAIDS report on the global AIDS epidemic 2013. Geneva: Joint United Nations Programme on HIV.* Aids, 2013.

20. Winskell, K., et al., *Social representations of the prevention of heterosexual transmission of HIV among young Africans from five countries, 1997-2014.* Plos one, 2020. **15**(3): p. e0227878.

21. Jia, K.M., et al., *Risk scores for predicting HIV incidence among adult heterosexual populations in sub‐Saharan Africa: a systematic review and meta‐analysis.* Journal of the International AIDS Society, 2022. **25**(1): p. e25861.

22. Nardell, M.F., et al., *Men missing from the HIV care continuum in sub‐Saharan Africa: a meta‐analysis and meta‐synthesis.* Journal of the International AIDS Society, 2022. **25**(3): p. e25889.

23. 2017, J.U.N.P.o.H.A. *Addressing a blind spot in the response to HIV: reaching out to men and boys. Geneva*. 2017; Available from: <http://www.unaids.org/en/resources/documents/2017/blind_spot>.

24. Johnson, L., et al., *Rates of HIV testing and diagnosis in South Africa: successes and challenges.* AIDS, 2015. **29**(11): p. 401-9.

25. Bicego, G.T., et al., *Recent patterns in population-based HIV prevalence in Swaziland.* PLoS One, 2013. **8**(10): p. e77101.

26. Uganda Bureau of Statistics Kampala, U., *Uganda Demographic and Health Survey: HIV/AIDs Related Knoledge, Attitudes, and Behaviour*. 2016: Kampala, Uganda and Rockville, Maryland, USA: UBOS and ICF.

27. Bureau, P.R., *Investing in Sexual and Reproductive Health is a Pathway to Better Health in Kalangala District*. 2018, Population Reference Bureau: Uganda.

28. Commission, U.A., *Facts on HIV and AIDS in Uganda 2021*. 2021: Kampala, Uganda.

29. Uganda Bureau of Statistics Kampala, U., *Uganda Demographic and Health Survey: HIV/AIDS related knowledge, attitudes, and behaviour*. 2016: Kampala Uganda and Rockville Maryland.

30. Uganda AIDS Commision, U., *Facts on HIV and AIDs in Uganda*, in *2022 Fact sheets*. 2022.

31. System, U.s.E.H.M.I. *Kalangala district health management information system*. eHMIS, Kalangala district 2023; Available from: <https://hmis2.health.go.ug/hmis2/dhis-web-commons/security/login.action>.

32. Brou, H., et al., *When do HIV-infected women disclose their HIV status to their male partner and why? A study in a PMTCT programme, Abidjan.* PLoS Med, 2007. **4**(12): p. e342.

33. Byamugisha, R., et al., *Determinants of male involvement in the prevention of mother-to-child transmission of HIV programme in Eastern Uganda: a cross-sectional survey.* Reprod Health, 2010. **7**: p. 12.

34. Reece, M., et al., *Assessing male spousal engagement with prevention of mother-to-child transmission (pMTCT) programs in western Kenya.* AIDS Care, 2010. **22**(6): p. 743-50.

35. Orne-Gliemann, J., et al., *Couple-oriented prenatal HIV counseling for HIV primary prevention: an acceptability study.* BMC Public Health, 2010. **10**: p. 197.

36. Falnes, E.F., et al., *"It is her responsibility": partner involvement in prevention of mother to child transmission of HIV programmes, northern Tanzania.* J Int AIDS Soc, 2011. **14**: p. 21.

37. Drugs, C.A.f. and T.i. Health, *Peer Support for Diabetes.* Heart Disease and HIV/AIDS: A Review of the Clinical Effectiveness, Cost-effectiveness, and Guidelines. Published on: November, 2013. **8**.

38. Colella, T.J. and K.M. King, *Peer support. An under-recognized resource in cardiac recovery.* European Journal of Cardiovascular Nursing, 2004. **3**(3): p. 211-217.

39. Solomon, P., *Peer support/peer provided services underlying processes, benefits, and critical ingredients.* Psychiatric rehabilitation journal, 2004. **27**(4): p. 392.

40. Nesta, *Peer support what is it and does it work?*, in *Summarising evidence from more than 1000 studies*.

41. Berg, R.C., S. Page, and A. Øgård-Repål, *The effectiveness of peer-support for people living with HIV: A systematic review and meta-analysis.* PloS one, 2021. **16**(6): p. e0252623.

42. HIV, N.s.f.p.s.i. *Positively UK. National standards for peer support in HIV. London: Positively UK. 2017*. 2017; Available from: <http://hivpeersupport.com/wp-content/uploads/2017/08/national_standards_final_web.pdf>.

43. Dennis, C.-L., *Peer support within a health care context: a concept analysis.* International journal of nursing studies, 2003. **40**(3): p. 321-332.

44. Doull, M., et al., *Peer support strategies for improving the health and well‐being of individuals with chronic diseases.* The Cochrane database of systematic reviews, 2017. **2017**(6).

45. Cohen S, G.B., Underwood LG. , *Social support measurement and intervention*, in *Measuring and intervening in social support*, G.B. Cohen S, Underwood LG, Editor. 2000: New York: Oxford University.

46. Delany-Moretlwe, S., et al., *Tenofovir 1% vaginal gel for prevention of HIV-1 infection in women in South Africa (FACTS-001): a phase 3, randomised, double-blind, placebo-controlled trial.* The Lancet infectious diseases, 2018. **18**(11): p. 1241-1250.

47. Seay, K., et al., *The vaginal acquisition and dissemination of HIV-1 infection in a novel transgenic mouse model is facilitated by coinfection with herpes simplex virus 2 and is inhibited by microbicide treatment.* Journal of virology, 2015. **89**(18): p. 9559-9570.

48. Leta, T.H., I.F. Sandøy, and K. Fylkesnes, *Factors affecting voluntary HIV counselling and testing among men in Ethiopia: a cross-sectional survey.* BMC public health, 2012. **12**: p. 1-12.

49. Scott-Sheldon, L.A., et al., *HIV testing is associated with increased knowledge and reductions in sexual risk behaviours among men in Cape Town, South Africa.* African journal of AIDS research, 2013. **12**(4): p. 195-201.

50. Ezeanolue, E.E., et al., *What do you need to get male partners of pregnant women tested for HIV in resource limited settings? The baby shower cluster randomized trial.* AIDS and Behavior, 2017. **21**: p. 587-596.

51. DiCarlo, A.L., et al., *‘Men usually say that HIV testing is for women’: gender dynamics and perceptions of HIV testing in Lesotho.* Culture, health & sexuality, 2014. **16**(8): p. 867-882.

52. Mhlongo, S., et al., *Factors associated with not testing for HIV and consistent condom use among men in Soweto, South Africa.* PLoS One, 2013. **8**(5): p. e62637.

53. Gage, A.J. and D. Ali, *Factors associated with self-reported HIV testing among men in Uganda.* AIDS care, 2005. **17**(2): p. 153-165.

54. Hlongwa, M., et al., *Mapping evidence of intervention strategies to improving men’s uptake to HIV testing services in sub-Saharan Africa: A systematic scoping review.* BMC Infectious Diseases, 2019. **19**(1): p. 496.

55. Wilson, T.E., et al., *HIV prevention for black heterosexual men: the barbershop talk with brothers cluster randomized trial.* American journal of public health, 2019. **109**(8): p. 1131-1137.

56. Medley, A., et al., *Effectiveness of peer education interventions for HIV prevention in developing countries: a systematic review and meta-analysis.* AIDS Education and Prevention, 2009. **21**(3): p. 181-206.

57. Shangani, S., et al., *Effectiveness of peer-led interventions to increase HIV testing among men who have sex with men: a systematic review and meta-analysis.* AIDS care, 2017. **29**(8): p. 1003-1013.

58. Simoni, J.M., et al., *Peer interventions to promote health: conceptual considerations.* American Journal of Orthopsychiatry, 2011. **81**(3): p. 351.

59. Bradford, J.B., S. Coleman, and W. Cunningham, *HIV System Navigation: an emerging model to improve HIV care access.* AIDS patient care and STDs, 2007. **21**(S1): p. S-49.

60. Genberg, B.L., et al., *Improving engagement in the HIV care cascade: a systematic review of interventions involving people living with HIV/AIDS as peers.* AIDS and Behavior, 2016. **20**(10): p. 2452-2463.

61. Sagar-Ouriaghli, I., et al., *Improving mental health help-seeking behaviours for male students: a framework for developing a complex intervention.* International journal of environmental research and public health, 2020. **17**(14): p. 4965.

62. Dooley, B.A. and A. Fitzgerald, *My world survey: National study of youth mental health in Ireland*. 2012: Headstrong and UCD School of Psychology.

63. Barry JA, K.R., Seager M, Sullivan L. , *The Palgrave handbook of male psychology and mental health*, ed. Springer. 2019.

64. Girault, P., et al., *Uptake of oral fluid-based HIV self-testing among men who have sex with men and transgender women in Thailand.* PLoS One, 2021. **16**(8): p. e0256094.

65. Health, U.M.o., *Consolidated Guidelines for the Prevention and Treatment of HIV and AIDS in Uganda*. 2020.

66. *Updated recommendations on HIV prevention, infant diagnosis, antiretroviral initiation and monitoring: March 2021.*, W.H. Organization, Editor. 2021: Geneva.

67. International, P.S., *HIV self-testing Africa initiative*. 2017, Population Services International Washington, D.C. .

68. Hlongwa, M., et al., *Linkage to HIV Care Following HIV Self-testing Among Men: Systematic Review of Quantitative and Qualitative Studies from Six Countries in Sub-Saharan Africa.* AIDS Behav, 2023. **27**(2): p. 651-666.

69. Bushby, B., et al., *Long-acting Injectable PrEP: A Scoping Review.* 2022.

70. Agrahari, V., et al., *Long-acting HIV pre-exposure prophylaxis (PrEP) approaches: recent advances, emerging technologies, and development challenges.* Expert opinion on drug delivery, 2022. **19**(10): p. 1365-1380.

71. Spinner, C.D., et al., *HIV pre-exposure prophylaxis (PrEP): a review of current knowledge of oral systemic HIV PrEP in humans.* Infection, 2016. **44**: p. 151-158.

72. Sidebottom, D., A.M. Ekström, and S. Strömdahl, *A systematic review of adherence to oral pre-exposure prophylaxis for HIV–how can we improve uptake and adherence?* BMC infectious diseases, 2018. **18**: p. 1-14.

73. Haberer, J.E., A. Mujugira, and K.H. Mayer, *The future of HIV pre-exposure prophylaxis adherence: reducing barriers and increasing opportunities.* The Lancet HIV, 2023. **10**(6): p. e404-e411.

74. Zhang, J., et al., *Discontinuation, suboptimal adherence, and reinitiation of oral HIV pre-exposure prophylaxis: a global systematic review and meta-analysis.* The lancet HIV, 2022. **9**(4): p. e254-e268.

75. Young, I.C., *Long-Acting Technologies for Prevention of HIV and Unplanned Pregnancy*. 2023, The University of North Carolina at Chapel Hill.

76. Liegeon, G. and J. Ghosn, *Long‐acting injectable cabotegravir for PrEP: a game‐changer in HIV prevention?* HIV medicine, 2023. **24**(6): p. 653-663.

77. Durham, S.H., et al., *Cabotegravir: the first long-acting injectable for HIV preexposure prophylaxis.* Annals of Pharmacotherapy, 2023. **57**(3): p. 306-316.

78. Hitchcock, A.M., et al., *Lenacapavir: A novel injectable HIV-1 capsid inhibitor.* International Journal of Antimicrobial Agents, 2024. **63**(1): p. 107009.

79. WHO. *Long-acting injectable lenacapavir continues to show promising results for HIV prevention*. 2024 [cited 2024 15/12/2024]; Available from: <https://www.who.int/news/item/26-09-2024-long-acting-injectable-lenacapavir-continues-to-show-promising-results-for-hiv-prevention>.

80. Website, G.P.P. *PrEP studies Overview*. Prevention With a Purpose 2024 [cited 2024 15/12/2024]; Available from: <https://www.purposestudies.com/>.

81. Hensen, B., et al., *Systematic review of strategies to increase men's HIV-testing in sub-Saharan Africa.* AIDS (London, England), 2014. **28**(14): p. 2133.

82. Ashaba, S., et al., *Community beliefs, HIV stigma, and depression among adolescents living with HIV in rural Uganda.* African Journal of AIDS Research, 2019. **18**(3): p. 169-180.

83. Hensen, B., et al., *Factors associated with HIV-testing and acceptance of an offer of home-based testing by men in rural Zambia.* AIDS and Behavior, 2015. **19**: p. 492-504.

84. Huerga, H., et al., *Progress towards the UNAIDS 90–90-90 goals by age and gender in a rural area of KwaZulu-Natal, South Africa: a household-based community cross-sectional survey.* BMC Public health, 2018. **18**(1): p. 1-8.

85. Leichliter, J.S., et al., *‘Clinics aren’t meant for men’: sexual health care access and seeking behaviours among men in Gauteng province, South Africa.* SAHARA-J: Journal of Social Aspects of HIV/AIDS, 2011. **8**(2).

86. Matovu, J.K., et al., *“Men are always scared to test with their partners… it is like taking them to the Police”: Motivations for and barriers to couples’ HIV counselling and testing in Rakai, Uganda: a qualitative study.* Journal of the International AIDS Society, 2014. **17**(1): p. 19160.

87. Wippold, G.M., et al., *Implementing barbershop-based health-promotion interventions for Black men: A systematic scoping review.* Health Psychol, 2023.

88. Wilson, T.E., et al., *HIV Prevention for Black Heterosexual Men: The Barbershop Talk with Brothers Cluster Randomized Trial.* Am J Public Health, 2019. **109**(8): p. 1131-1137.

89. (UNAIDS), J.U.N.P.o.H.A., *Confronting Inequalities: Lessons for pandemic responses from 40 years of AIDS*. 2021.

90. Mayne, J., *The COM-B theory of change model.* unpublished www. researchgate. net/publication/314086441_The_COM-B_Theory_of_Change_Model_V3 (accessed July 22, 2019), 2018.

91. Semitala, F.C., et al., *Acceptance and completion of rifapentine-based TB preventive therapy (3HP) among people living with HIV (PLHIV) in Kampala, Uganda—patient and health worker perspectives.* Implementation science communications, 2021. **2**(1): p. 1-12.

92. Carroll, C., et al., *A conceptual framework for implementation fidelity.* Implementation Science, 2007. **2**(1): p. 40.

93. *Overview: Kalangala District*. 2023 [cited 2023; Available from: <https://kalangala.go.ug/lg/overview>

94. Richard J. Hayes, L.H.M., *Cluster Randomised Trials, Second Edition,* . 2017, Boca Raton, FL: CRC Press.

Appendix I: Schedule of Study Visits and Procedures

APpendix Ia: Schedule of Events for Participants

|  | Screening | Enrollment | Week 26 | Week 52 |
| --- | --- | --- | --- | --- |
| **Administrative and Behavioral Procedures** | | | | |
| Informed Consent | X |  |  |  |
| Locator information | X | X | X | X |
| Demographic information | X | X |  |  |
| Social impacts assessment |  | X | X | X |
| Socio-behavioral assessment | X | X | X | X |
| Disclose study group (intervention or control) |  | X |  |  |
| Intervention acceptability assessment |  | X | X | X |
| HIV self-testing assessment |  |  | X | X |
| HIV counseling and testing | X |  | X | X |
| Provide information about recommended HIV testing schedule |  | X | X | X |
| Offer condoms | X | X | X | X |
| In-depth interviews^1,2^ |  |  | X | X |
| Long-Acting PrEP Interest Questionnaire |  |  |  | X |
| **Clinical Procedures** | | | | |
| Complete medical history |  | X |  |  |
| Targeted medical history |  |  | X | X |
| Symptom directed physical exam |  | X | X | X |
| Concomitant drug use |  | X | X | X |
| Blood collection | X |  | X | X |
| Urine collection |  | X | X | X |
| HIV prevention services or HIV care referral | X | X | X | X |
| STI treatment^3^ | X | X | X | X |
| **Laboratory Procedures** | | | | |
| HIV diagnostic testing^3^ | X |  | X | X |
| STI testing: blood for syphilis | X |  | X^4^ | X^4^ |
| STI testing: urine NAAT for gonorrhea and chlamydia |  | X | X | X |
| Blood storage for LL QC |  |  | X | X |

^1^Intervention group only

^2^ Subset of men only

^3^ HIV RNA testing required if participant has acute symptoms of HIV

^4^ If clinically indicated

**Appendix Ib: Schedule of Events for Barbers**

|  | Study Initiation | Weeks 13/39/65 | | Weeks 26/52 |
| --- | --- | --- | --- | --- |
| **Administrative Procedures** | | | | |
| Informed Consent | X |  | |  |
| Memorandum of understanding | X |  | |  |
| Locator information | X |  | |  |
| Collect barber demographics | X |  | |  |
| Collect barbershop details | X |  | |  |
| Training on study and recruitment procedures | X |  | |  |
| Training on barbershop-based intervention procedures^1^ | X |  | |  |
| Social impacts assessment^2^ |  | X | | X |
| Intervention acceptability and feasibility assessment^1^ |  | X | | X |
| In-depth interviews^1^ |  |  | | X |
|  | | | | |
|  | During client-visits to the barbershop | | Every two months | |
| **Barber-led Procedures** |  | |  | |
| Refer clients for study screening/enrollment | X | |  | |
| Record number of clients referred to study | X | |  | |
| Provide HIV education^1^ | X | |  | |
| Provide HIVST kits^1^ | X | |  | |
| Record number of HIVST kits distributed and services provided^1^ | X | | X | |
| HIV prevention services referral^1^ | X | |  | |
| Offer condoms^1^ | X | |  | |
| Lead group sessions^1^ |  | | X | |

^1^ Intervention barbers only

^2^ During enrollment phase for control barbers and during enrollment and follow-up for intervention barbers

Appendix IIA: Sample Informed Consent (participant)

**HPTN 111**

**Feasibility and Acceptability of a Barbershop Based HIV Prevention Initiative Among Heterosexual Men in Kalangala Islands, Uganda: A Cluster Randomized Trial**

**Participant Informed Consent Form**

**Version 2.0**

**31 January 2025**

**DAIDS Document ID:** **39062**

**Sponsored by:** Division of AIDS, US National Institute of Allergy and Infectious Diseases, US National Institutes of Health.

**PRINCIPAL INVESTIGATOR:** [Insert Name]

**PHONE:** [Insert Number]

**INTRODUCTION**

We are asking you to take part in a research study. Joining this study is voluntary. You may refuse to join, or you may change your mind and decide you do not want to be in the study, for any reason, at any time. This research study is for men who live in an area where there may be many people living with Human Immunodeficiency Virus, or HIV. It is also for men who might be more likely to get or acquire HIV themselves. HIV is the virus that causes Acquired Immunodeficiency Syndrome, or AIDS.

Before you decide whether to join the study, we would like to explain the purpose of the study, the risks and benefits to you, and what is expected of you.

1. **You should know key information about this study before you join.**

Here is a summary of important information about the study:

- This is a research study.
- Your participation in this study is voluntary. You can decide not to be in this study at any time.
- We are trying to learn if it is possible for barbers to give HIV prevention services, like education and HIV self-test kits, at barbershops.
- We want to know if men like receiving HIV prevention services at a barbershop and if it helps to increase the number of men getting an HIV test and going to the clinic for HIV prevention or treatment services.
- We have chosen different barbershops in Kalangala district to be part of this study. Some shops were randomly chosen to train barbers to provide HIV prevention services. Other shops were randomly chosen to continue with the standard-of-care, meaning you would be referred to health centers or community services for HIV prevention services but would not get any counseling or tests from your barber. Depending on which shop you use, you will either receive the intervention from your barber or the standard-of-care.
- This study has one visit today. If you are eligible and want to participate after today, then you will come to the clinic for a study visit in 6 months and 12 months. Aside from these times, you can keep to your normal schedule and attend your barbershop for barber services.
- At each study visit we will ask you questions about your medical history, HIV testing, and sexual behavior. We will also ask your permission to collect blood and urine for HIV and sexually transmitted infection (STI) testing.
- There are minimal risks to you if you decide to participate in the study. You may be uncomfortable answering questions or having HIV/STI testing. We will do our best to protect your information, but it is possible other people may learn of your participation in this study. If you are in a shop that provides HIV prevention services, we will have to tell your barber that you are part of the study.
- There may be no direct benefit to you if you decide to participate in the study. However, you may benefit from HIV and STI testing and treatment. You may also benefit from the information you learn from the barber or study staff about HIV prevention.
- If you decide not to participate in the study, you may receive HIV/STI testing, or HIV prevention counseling at local health clinics.

This form gives more information about the study. You should feel that you understand what the study is about before deciding whether you will participate.

**ABOUT THE STUDY**

The HIV Prevention Trials Network (HPTN) and MU-JHU are doing this study to understand if it is possible for barbers to provide HIV prevention services, like counseling and HIV self-test kits, at barbershops. We also want to know if men like receiving these services at a barbershop and if it helps to increase the number of men getting an HIV test or going to the clinic for HIV prevention or treatment services.

About 250 people will participate in this study from Kalangala District, Uganda. Participants will be in the study for about one year.

1. **This study is testing if barbers can provide HIV prevention services to men.**

Men and boys are less likely to test for HIV and receive treatment compared to women. It can be difficult for men to test and get HIV prevention services for many reasons. Health clinics can be hard to get to or may only be open when people are working during the day. Sometimes men feel judged by health care providers. We want to see if a barber can provide HIV prevention services and if men would like these services. The barber is often a trusted community member and may be able to help men get tested and receive care. The study may show a new way to improve the health of men who find it hard to engage in HIV prevention in other ways.

1. **Barbershops in the study will be placed in one of two groups. Participants will receive services depending on their shop.**

All the barbershops included in the study were randomly put into one of two groups. Random means the study staff did not choose and the barber did not choose. It is like rolling a dice. The difference between the shops is that one group will have trained barbers. These trained barbers will provide clients in the study with HIV education, give them HIV self-test kits, and invite them to group sessions to discuss HIV prevention. The other group of shops will continue to have the standard-of-care HIV prevention services in health facilities. For every two barbershops randomly assigned to have trained barbers, one barbershop will be assigned to provide the standard-of-care. This is called a 2:1 randomization.

If your shop is in the group with trained barbers, you will receive these HIV prevention services from your barber. If your shop is in the other group, you will not receive these services from your barber. All participants will have study visits approximately six months and twelve months after starting the study.

**JOINING THE STUDY**

1. **It is your decision whether to participate in the study.**

This consent form gives information about the study that we will discuss together. We will help you understand the form and answer your questions before you sign this form. Once you understand the study, and if you agree to take part, you will be asked to sign your name or make your mark on this form. You will be offered a copy of this form to keep.

Before you learn about the study, it is important that you know the following:

- Your participation is voluntary. You do not have to take part in any of the tests or procedures in the study.
- You may decide not to take part in the study, or you may decide to leave the study at any time without losing your regular medical care. You can also continue to attend your barber for haircuts without being in the study.
- If you decide not to take part in the study, you can still join another study at a later time if there is one available and you qualify.
- You cannot join this study if you are taking part in another study of drugs or medical devices. You are asked to tell the study staff about any other studies you are taking part in or thinking of taking part in. This is very important for your safety.

1. **You must qualify before you can join the study.**

If you decide to join this study, we will first do some tests and collect some information from you to find out if you qualify. These tests and the information collected are described in #6 below. If you do not qualify, you cannot join the study.

1. **We will ask you questions, examine you, and test your blood and urine.**

To find out if you qualify, we will first ask some questions and do some tests. This will happen after you read, discuss, understand, and sign this form. These tasks will take about 30 minutes.

At the Screening Visit, we will:

- Ask you questions about yourself, like your age
- Ask you to answer questions on a computer about your sexual practices
- Ask about where you live and how to contact you
- Talk with you about HIV and ways to protect yourself from getting it. We will also offer you condoms.
- Collect ~10mL (about 2 teaspoons) of blood for HIV and syphilis testing. Some blood will be stored during the study for quality control testing. Quality control testing is used to make sure HIV testing is working the way it should in the laboratory.

Your HIV test results will be available during the visit. You will be contacted about the results of your other tests when they are available. If you test positive for syphilis, you will be provided or referred for treatment.

1. **If you qualify and are interested, you will join the study.**

We will review your test results and the information you provide today. If you have a positive HIV test you will not be eligible for the study, and you will be referred for the appropriate medical care.

If you are eligible for this study and decide to join, you will be asked to continue with today’s visit for an “Enrollment Visit.” If you are unable to continue with the visit today, you may come back to the clinic on another day to finish the Enrollment Visit. We ask that you come for enrollment within two weeks. This visit will last about 2 hours.

During the Enrollment Visit, we will:

- Confirm information already provided
- Ask you questions about yourself, like your use of HIV prevention methods, medical history, and if you are taking any medication.
- Ask you to answer questions on a computer about your sexual practices
- If you report any medical concerns, we will give you a brief physical exam.
- Ask you to provide urine for gonorrhea and chlamydia testing
- Provide treatment if you have symptoms of an STI
- Tell you if your barber is providing the intervention

**BEING IN THE STUDY**

1. **You will have up to two study visits over 12 months.**

If you decide to join the study, you will be asked to come to this clinic six months and twelve months after your enrollment visit.

Each visit will last about 2 hours.

During these study visits, we will:

- Confirm where you live and how to contact you
- Ask you questions about yourself, like your use of HIV prevention methods, updates to your medical history, and if you are taking any new medication.
- Ask you to answer questions on a computer about your sexual practices
- Talk with you about HIV and ways to protect yourself from getting it. We will also offer you condoms.
- If you report any medical concerns, we will give you a brief physical exam.
- Collect ~10 mL (about 2 teaspoons) of blood for HIV testing. Some blood will be stored during the study for quality control testing. Quality control testing is used to make sure HIV testing is working the way it should in the laboratory.
- Ask you to provide urine for gonorrhea and chlamydia testing
- Provide treatment if you have symptoms of an STI

You may have more visits if you are sick or we need to check on your health.

At your final study visit, we will ask you questions about interest in long-acting PrEP and will talk with you about the end of the study and when the results of the study will be available.

1. **If you get HIV during the study, we will help you get care and support.**

We will test your blood for HIV during this study. If you get HIV while you are in the study, we will help you find the care and support you need. We will ask you to come back for your study visits after confirming your HIV status. We will examine you and ask you about any medications you are taking. We will also provide you with condoms.

If you attend a barbershop with a trained barber, you can decide if you want the barber to stop providing HIV education and services. You may tell your barber to stop providing HIV services. Or, if you want, study staff can tell them to stop providing HIV services. You will be able to continue with your regular haircut services.

1. **You will continue to receive haircuts from your barber.**

If your barbershop is in the group with trained barbers, we will tell your barber that you have joined the study. We will also ask you to tell your barber that you are in the study. At your haircuts with the barber, they will:

- Provide you with HIV education
- Give you HIV self-test kits to take with you
- Invite you to group sessions that will happen about every two months
- Record the number of times you come for a haircut, receive counseling, and take HIV self-test kits. Your barber will give this information to study staff.

If your barbershop is in the group without trained barbers, you will not receive any of the HIV prevention services from your barber. We will provide you with information about HIV prevention services available to you in the community.

1. **We may ask you to participate in a long discussion about the study**

Some participants will be asked to come for an interview with study staff. At the interview we will ask:

- How you feel about the barbershop-based services
- Your feelings about HIV and HIV prevention services
- About your relationships and sexual behaviors

The interviews require one or two visits and will last around 1 hour. During the interview we will audio record your responses and then later write them down (called a transcript). The written transcript will not include any identifying information. The audio recordings will be destroyed after all analysis is completed.

1. **Use of samples.**

Blood and urine samples will only be used for HIV and STI testing and quality control. Quality control testing is used to make sure HIV testing is working the way it should in the laboratory.

Biospecimens collected for this study will only be used for this study and will be destroyed at the end of the study. The specimens will not be shared or used for other studies.

**RISKS OF THE STUDY**

1. **There may be risks to being in this study.**

*STUDY PROCEDURES*

Taking blood samples may cause some pain, bruise your arm, or make you feel lightheaded. In rare cases you may faint. There is also a slight chance of infection when blood is drawn. You may be nervous while you are waiting for your HIV test result. If the tests show that you have HIV, you may worry about your health and future. You will receive counseling before and after the test to help address your concerns.

*DISCLOSURE OF PERSONAL INFORMATION*

We will make every effort to protect your confidentiality during the study. However, it is possible that others may learn that you are part of this study and they may think that you are living with HIV or are at increased likelihood of getting HIV. Because of this you could have trouble finding or keeping a job. You could also have problems with your family, friends and community.

If you were told about this study by your barber, it is possible they may think they know your HIV status based on your enrollment in the study or if you decide to stop the study. We will not tell the barber your HIV status. If you attend the group sessions with a trained barber, we cannot guarantee that other participants will keep the discussions private.

We will not tell anyone about your HIV status or if you have a sexually transmitted infection without your permission. We can tell you more about how we will protect your information.

*SENSITIVE QUESTIONS*

The questions we will ask you about your sexual behavior may make you feel uneasy. However, you do not have to answer any question that you do not want to and you can stop answering the questions at any time.

*SOCIAL*

There may also be some social risks to participating in this study. You may feel embarrassed or uncomfortable with some of the questions you will be asked, some of the procedures that will be done, or some of the test results that you will receive. You may also experience stigma as a result of being involved in a study about HIV because people may assume that you are living with HIV. Family or friends may worry, get upset or angry, or assume that you are living that there is a high likelihood of you getting HIV and treat you unfairly as a result.

*OTHER RISKS*

We do not know if there are other risks if you participate in this study. You should report any problems to the researchers immediately.

**BENEFITS OF THE STUDY**

1. **There may be no direct benefit to you by participating in the study.**

Since we do not know if the barbershop-based HIV prevention services help men, there may be no direct benefit to you to being in the study. However, being in the study may still help you in some ways. We will test you for HIV and other sexually transmitted infections throughout this study. The counseling you get from study staff may help you to avoid HIV and other sexually transmitted infections. If you are living with or get HIV, this counseling may help you to learn how to better care for yourself and avoid passing HIV to others. Participants who go to a barber who is delivering the HIV services may benefit from the information provided by the barber or from getting an HIV self-test kit.

If you get HIV, or have another sexually transmitted infection, we will treat you or refer you for care and/or treatment. At every visit you will receive condoms free of charge.

You may not receive any other direct benefit from being in this study; however, you or others in your community may benefit from this study later. The information gathered during this study may help to prevent HIV and other infections. This may be beneficial to you and your community.

**OTHER INFORMATION ABOUT THE STUDY**

1. **We will tell you any new information that may affect your decision to be in the study.**

You will be told any new information learned during this study that might affect your willingness to stay in the study. For example, if information becomes available that shows that the barbershop-based HIV prevention services seem to work very well or do not work very well, you will be told about this. You will also be told when the results of the study may be available, and how to learn about them.

1. **You may be withdrawn from the study without your consent.**

We may take you out of the study at any time without your consent. This may happen if:

- You are unable or unwilling to follow all of the study procedures or instructions.
- The study is stopped or canceled.
- The study staff feels that staying in the study would be harmful to you.
- Other reasons, as decided by the study staff
- If we take you out of the study, we may ask you to come back to the clinic one last time to check your blood and urine and ask you questions

1. **You have other choices if you choose not to be in this study.**

There may be other studies going on here or in the community that you may qualify for. If you wish, we will tell you about other studies that we know about. There also may be other places where you can go for HIV counseling and testing. We will tell you about those places if you wish.

1. **There is no cost to you to be in this study.**

There will be no cost to you for study related visits, physical examinations, laboratory tests, or other procedures.

1. **We will give you [site to insert amount] for each study visit.**

You will receive [*$xx*] for your time, effort, and travel to and from the clinic at each scheduled visit. [*Site to insert information about local reimbursement for the study.]*

1. **We will do our best to protect your private information.**

Every effort will be made to keep your personal information confidential, but we cannot guarantee absolute confidentiality. To keep your information private, your personal information and samples will be labeled with a code, after removing information that could identify you. The coded information can only be traced back to your study clinic. Your name, where you live, and other personal information will be protected by the study clinic. Your coded information, without personal identifiers, could be used for future research studies or distributed to another investigator for future studies without additional informed consent being obtained.

The results of any tests done on your samples will not be included in your health records. Personal information from your study records will not be released without your written permission. Any publication of this study will not use your name or identify you personally. Your personal information may be disclosed if required by law.

Study staff will have access to your study records. Your records may also be reviewed, under guidelines of the US Federal Privacy Act, by:

- Uganda Joint Clinical Research Center (JCRC) IRB
- The Uganda National Council for Science and Technology
- Uganda Ministry of Health (MOH)
- Uganda National Health Research Organization (UNHRO)
- The sponsor of the study (US National Institutes of Health [NIH]), its contractors, and its study monitors
- The US Office for Human Research Protections (OHRP)
- Other local, US, or international regulatory authorities/entities
- The HPTN that is conducting this study

The study staff will also use your personal information, if needed, to verify that you are not taking part in any other research studies. This includes other studies conducted by MU-JHU and studies conducted by other researchers that study staff know about.

A description of this clinical trial will be available on <http://www.ClinicalTrials.gov>. This Web site will not include information that can identify you. At most, the Web site will include a summary of the results. You can search this Web site at any time.

1. **If you get sick or injured during the study, contact us immediately.**

It is unlikely that you will be injured as a result of study participation. If you are injured, the study team will give you immediate necessary treatment for your injuries. You will not have to pay for this treatment. If the study team cannot treat your injury, you will be referred to a facility that can treat you. There is no program to pay money or give other forms of compensation for such injuries either through this institution or the US NIH. You do not give up any legal rights by signing this consent form.

1. **Contact us at any time if you have questions or problems.**

If you ever have any questions about the study, or if you have a research-related injury, you should contact *[insert name of the investigator or other study staff]* at *[insert telephone number and/or physical address*].

If you have questions about your rights as a research participant, you should contact [*insert name or title of person on the IRB or other organization appropriate for the site*] at [*insert physical address and telephone number].*

If you have questions about who to contact at the research site, you should contact [*insert name of the investigator or community educator or Community Advisory Board member*] at [*insert physical address and telephone number].*

**SIGNATURE PAGE**

**HPTN 111**

**Feasibility and Acceptability of a Barbershop Based HIV Prevention Initiative Among Heterosexual Men in Kalangala Islands, Uganda: A Cluster Randomized Trial Participant Informed Consent Form**

**Version 2.0**

**31 January 2025**

Screening and Enrollment Consent

If you have read this consent form, or had it read and explained to you, and you understand the information, and you voluntarily agree to join the study, please sign your name or make your mark below. Also, please indicate by providing your initials in the spaces below if you agree to have a study visit outside of the study clinic or participate in an interview.

____ I agree to take part in this study.

_____ I agree to complete study visits at a location outside of the study clinic. Study staff will coordinate with me about an acceptable location.

____ I do not agree to complete study visits at a location outside of the study clinic.

____ I agree to participate in an interview where I will be asked questions about this research, and the interview will be recorded.

____ I do not agree to participate in an interview where I will be asked questions about this research, and the interview will be recorded.

________________________________ _____________________________________

Participant Name (print) Participant Signature and Date

________________________________ _____________________________________

Study Staff Conducting Consent Discussion (print) Study Staff Signature and Date

________________________________ _____________________________________

Witness Name (print) (As appropriate) Witness Signature and Date

Appendix IIB: Sample Informed Consent (Barber)

**HPTN 111**

**Feasibility and Acceptability of a Barbershop Based HIV Prevention Initiative Among Heterosexual Men in Kalangala Islands, Uganda: A Cluster Randomized Trial**

**Barber Informed Consent Form**

**Version 2.0**

**31 January 2025**

**DAIDS Document ID: 39062**

**Sponsored by:** Division of AIDS, US National Institute of Allergy and Infectious Diseases, US National Institutes of Health.

**PRINCIPAL INVESTIGATOR:** [Insert Name]

**PHONE:** [Insert Number]

**INTRODUCTION**

We are asking you to take part in a research study. Joining this study is voluntary. You may refuse to join, or you may withdraw your consent to be in the study, for any reason. This research study is for barbers who are participating in a barbershop-based program for HIV prevention.

Before you decide whether to join the study, we would like to explain the purpose of the study, the risks and benefits to you, and what is expected of you.

1. **You should know key information about this study before you join.**

Here is a summary of important information about the study:

- This is a research study.
- Your participation in this study is voluntary.
- We are trying to learn if it is possible for barbers to provide HIV prevention services, like education and HIV self-test kits, at barbershops. We also want to know if barbers like providing these services and if it helps to increase the number of men getting an HIV test and going to the clinic for HIV prevention or treatment services.
- We have chosen different barbershops in Kalangala district to be part of this study. Some shops will be randomly chosen to train barbers to provide HIV prevention services. Other shops will be randomly chosen to continue with no HIV prevention services. Depending on which shop you work in, you will either deliver the intervention from your shop or not. All barbers in both groups will help refer clients to the study.
- This study has up to six visits with study staff after today. If you are eligible and want to participate after today, then you will have an initial visit. If you work at a shop chose to deliver the intervention, you will have a visit in approximately 6 and 12 months at the study clinic to talk about your experiences with the program. We will also ask some questions about the program when we come visit you at your shop in approximately 3, 9 and 15 months.
- At each study visit we will ask questions about how you feel about the program and if you like providing HIV prevention services to your clients.
- There are minimal risks to you if you decide to participate in the study. You may be uncomfortable answering questions about your experiences. We will do our best to protect your information, but it is possible other people may learn of your participation in this study. If other people learn about your participation, it may impact whether clients want to attend your shop.
- There may be no direct benefit to you if you decide to participate in the study. However, you may benefit from the information you learn from study staff about HIV prevention.
- If you decide not to participate in the study, you may learn about HIV prevention at local health clinics.

This form gives more information about the study. You should feel that you understand the study before deciding whether you will participate.

**ABOUT THE STUDY**

The HIV Prevention Trials Network (HPTN) and MU-JHU are doing this study to understand if it is possible for barbers to provide HIV prevention services, like education and HIV self-test kits, at barbershops. We also want to know if barbers like providing these services at their shop and if it helps to increase the number of men getting an HIV test or going to the clinic for HIV prevention or treatment services.

About 18 barbers will participate in this study from Kalangala district, Uganda. Barbers will be in the study for approximately 15 months.

1. **This study is testing if barbers can provide HIV prevention services to men.**

Men and boys are less likely to test for HIV and receive treatment compared to women. It can be difficult for men to test and get HIV prevention services for many reasons. Health clinics can be hard to get to or may only be open when men are working during the day. Sometimes men feel judged by health care providers. We want to see if a barber can provide HIV prevention services and if men would like these services. We also want to know if barbers like providing these services. The barber is often a trusted community member and may be able to help men get tested and receive care. The study may show a new way to improve the health of men who find it hard to engage in HIV prevention in other ways.

1. **Barbershops in the study will be placed in one of two groups. Clients in the study will receive services depending on their shop.**

All the barbershops included in the study will be randomly put into one of two groups. Random means the study staff did not choose and the barbers did not choose. It is like rolling a dice. The difference between shops is that one group will have barbershops with trained barbers. These trained barbers will provide clients in the study with HIV education, give them HIV self-test kits, and invite them to group sessions to discuss HIV prevention. The other group of barbershops will not deliver any HIV prevention services. Men at these shops will continue to have the standard-of-care HIV prevention services in health facilities. For every two barbershops randomly assigned to have trained barbers, one barbershop will be assigned to provide the standard-of-care. This is called a 2:1 randomization.

Barbers in both groups will help refer clients to the study. Before you help recruit clients or deliver the services, you will attend a training to learn about the study and the services.

**JOINING THE STUDY**

1. **It is your decision whether to participate in the study.**

This consent form gives information about the study that will be discussed with you. We will help you understand the form and answer your questions before you sign this form. Once you understand the study, and if you agree to take part, you will be asked to sign your name or make your mark on this form. You will be offered a copy of this form to keep.

Before you learn about the study, it is important that you know the following:

- Your participation is voluntary. You do not have to take part in any of the procedures in the study.
- You may decide not to take part in the study, or you may decide to leave the study at any time.
- If you decide not to take part in the study, you can still join another study at a later time if there is one available and you qualify.

**BEING IN THE STUDY**

1. **You will refer clients to study staff. If you are in the group of shops with trained barbers, you will deliver HIV prevention services to your clients who are in the study. You will also have your own study visits.**

If you decide to join the study, both groups of barbers will:

- Be trained on how to keep your client’s confidentiality.
- Be trained on the study and how to recruit clients. Training will take approximately 1-2 days.
- Provide information about your shop and experiences as a barber.
- Provide information about your experience referring clients to the study. You will also be asked to complete a simple checklist that includes the number of clients you refer to the study.

If you are in the group chosen to deliver HIV prevention to clients, you will:

- Be trained on how to provide education and HIV test kits. This extra training will take another 2-3 days.
- Provide peer support groups every two months.
- Complete a simple checklist of the services provided to study participants.
- You will have up to six study visits during the 15 months. These visits will occur approximately quarterly. At some study visits you will be asked to provide information about your experience and opinions about delivering HIV prevention services in this study. We will ask these questions when we visit you at your shop.
- At the visits at 6 and 12 months at the study clinic, we will ask questions in an individual interview. During the interview we will audio record your responses and then later write them down (called a transcript). The written transcript will not include any identifying information. The audio recordings will be destroyed after all analysis is completed.

Each study visit will last about one hour.

**RISKS OF THE STUDY**

1. **There may be risks to being in this study.**

*DISCLOSURE OF PERSONAL INFORMATION*

We will make every effort to protect your confidentiality during the study. However, it is possible that others may learn that you are part of this study, and they may not be comfortable with your participation. Because of this you could have trouble finding or keeping a job or it may mean that some clients do not want to come to your barbershop. You could also have problems with your family, friends, and community. We can tell you more about how we will protect your information.

*SENSITIVE QUESTIONS*

It is possible that the questions we ask you about your experiences will make you feel uneasy. However, you do not have to answer any question that you do not want to and you can stop answering the questions at any time.

*SOCIAL*

There may also be some social risks to participating in this study. You may experience stigma as a result of being involved in a study about HIV. It is possible that clients may not want come for haircut services if they know you are part of this study or associate your shop with HIV and sexually transmitted infections.

**BENEFITS OF THE STUDY**

.

1. **There may be no direct benefit to you by participating in the study.**

There may be no direct benefit to you to being in the study. However, you or others in your community may benefit from this study later. The information gathered during this study may help to prevent HIV and other infections. It is possible that the information you learn during study training will help you understand how to prevent HIV.

**OTHER INFORMATION ABOUT THE STUDY**

1. **We will tell you any new information that may affect your decision to be in the study.**

You will be told any new information learned during this study that might affect your willingness to stay in the study. You will also be told when the results of the study may be available, and how to learn about them.

1. **You may be withdrawn from the study without your consent.**

We may take you out of the study at any time without your consent. This may happen if:

- You are unable or unwilling to follow all of the study procedures or instructions.
- You are unable to refer enough clients to study staff.
- The study is stopped or canceled.
- The study staff feels that staying in the study would be harmful to you.
- Other reasons, as decided by the study staff.

1. **You have other choices if you choose not to be in this study.**

There may be other studies going on here or in the community that you may be eligible for. If you wish, we will tell you about other studies that we know about.

1. **There is no cost to you to be in this study.**

There will be no cost to you for the study visits.

1. **We will give you [*site to insert amount*] for each study visit and up to [*site to insert amount*] for providing the HIV prevention services to your clients.**

You will receive [*$xx*] for your time, effort, and travel for each visit. You will receive up to [*$xx*] each month for your time and effort providing the study services to clients.

1. **We will do our best to protect your private information.**

Every effort will be made to keep your personal information confidential, but we cannot guarantee absolute confidentiality. You will be identified by a code. The coded information can only be traced back to your study clinic. Your name, where you live, and other personal information will be protected by the study clinic. Your coded information, without personal identifiers, could be used for future research studies or distributed to another investigator for future studies without additional informed consent being obtained. Personal information from your records will not be released without your written permission. Any publication of this study will not use your name or identify you personally. Your personal information may be disclosed if required by law.

Study staff will have access to your study records. Your records may also be reviewed, under guidelines of the US Federal Privacy Act, by:

- Uganda Joint Clinical Research Center (JCRC) IRB
- The Uganda National Council for Science and Technology
- Uganda Ministry of Health (MOH)
- Uganda National Health Research Organization (UNHRO)
- The sponsor of the study (US National Institutes of Health [NIH]), its contractors, and its study monitors
- The US Office for Human Research Protections (OHRP)
- Other local, US, or international regulatory authorities/entities
- The HPTN that is conducting this study

A description of this clinical trial will be available on <http://www.ClinicalTrials.gov>. This Web site will not include information that can identify you. At most, the Web site will include a summary of the results. You can search this Web site at any time.

1. **If you get injured during the study, contact us immediately.**

It is unlikely that you will be injured as a result of study participation. If you are injured, the study team will give you immediate necessary treatment for your injuries. You will not have to pay for this treatment. You will be told where you can get additional treatment for your injuries. There is no program to pay money or give other forms of compensation for such injuries either through this institution or the US NIH. You do not give up any legal rights by signing this consent form.

1. **Contact us at any time if you have questions or problems.**

If you ever have any questions about the study, or if you have a research-related injury, you should contact [*insert name of the investigator or other study staff] at [insert telephone number and/or physical address*].

If you have questions about your rights as a research participant, you should contact [*insert name or title of person on the IRB or other organization appropriate for the site*] at [*insert physical address and telephone number*].

If you have questions about who to contact at the research site, you should contact [*insert name of the investigator or community educator or Community Advisory Board member*] at [*insert physical address and telephone number].*

**SIGNATURE PAGE**

**HPTN 111**

**Feasibility and Acceptability of a Barbershop Based HIV Prevention Initiative Among Heterosexual Men in Kalangala Islands, Uganda: A Cluster Randomized Trial Participant Informed Consent Form**

**Version 2.0**

**31 January 2025**

Barber Consent

If you have read this consent form, or had it read and explained to you, and you understand the information, and you voluntarily agree to join the study, please sign your name or make your mark below.

____ I agree to take part in this study.

________________________________ _____________________________________

Participant Name (print) Participant Signature and Date

________________________________ _____________________________________

Study Staff Conducting Consent Discussion (print) Study Staff Signature and Date

________________________________ _____________________________________

Witness Name (print) (As appropriate) Witness Signature and Date
