# Supplementary material for: Feasibility and Acceptability of Barbershop-Based HIV Prevention Among Heterosexual Men in Kalangala Islands, Uganda: Protocol for a Cluster Randomized Trial (HPTN 111)
Source: JMIR Res Protoc. 2026 Apr 17;15:e87612. doi: 10.2196/87612 (PMC13135168; doi:10.2196/87612)
Supplement: Multimedia Appendix 2 [file resprot_v15i1e87612_app2.docx]

Sample Informed Consent (participant)

**HPTN 111**

**Feasibility and Acceptability of a Barbershop Based HIV Prevention Initiative Among Heterosexual Men in Kalangala Islands, Uganda: A Cluster Randomized Trial**

**Participant Informed Consent Form**

**Version 1.0**

**18 September 2023**

**DAIDS Document ID:** **39062**

**Sponsored by:** Division of AIDS, US National Institute of Allergy and Infectious Diseases, US National Institutes of Health.

**PRINCIPAL INVESTIGATOR:** [Insert Name]

**PHONE:** [Insert Number]

**INTRODUCTION**

We are asking you to take part in a research study. Joining this study is voluntary. You may refuse to join, or you may change your mind and decide you do not want to be in the study, for any reason, at any time. This research study is for men who live in an area where there may be many people living with Human Immunodeficiency Virus, or HIV. It is also for men who might be more likely to get or acquire HIV themselves. HIV is the virus that causes Acquired Immunodeficiency Syndrome, or AIDS.

Before you decide whether to join the study, we would like to explain the purpose of the study, the risks and benefits to you, and what is expected of you.

1. **You should know key information about this study before you join.**

Here is a summary of important information about the study:

- This is a research study.
- Your participation in this study is voluntary. You can decide not to be in this study at any time.
- We are trying to learn if it is possible for barbers to give HIV prevention services, like education and HIV self-test kits, at barbershops.
- We want to know if men like receiving HIV prevention services at a barbershop and if it helps to increase the number of men getting an HIV test and going to the clinic for HIV prevention or treatment services.
- We have chosen different barbershops in Kalangala district to be part of this study. Some shops were randomly chosen to train barbers to provide HIV prevention services. Other shops were randomly chosen to continue with the standard-of-care, meaning you would be referred to health centers or community services for HIV prevention services but would not get any counseling or tests from your barber. Depending on which shop you use, you will either receive the intervention from your barber or the standard-of-care.
- This study has one visit today. If you are eligible and want to participate after today, then you will come to the clinic for a study visit in 6 months. You may also be asked to come for a visit in 12 months. Aside from these times, you can keep to your normal schedule and attend your barbershop for barber services.
- At each study visit we will ask you questions about your medical history, HIV testing, and sexual behavior. We will also ask your permission to collect blood and urine for HIV and sexually transmitted infection (STI) testing.
- There are minimal risks to you if you decide to participate in the study. You may be uncomfortable answering questions or having HIV/STI testing. We will do our best to protect your information, but it is possible other people may learn of your participation in this study. If you are in a shop that provides HIV prevention services, we will have to tell your barber that you are part of the study.
- There may be no direct benefit to you if you decide to participate in the study. However, you may benefit from HIV and STI testing and treatment. You may also benefit from the information you learn from the barber or study staff about HIV prevention.
- If you decide not to participate in the study, you may receive HIV/STI testing, or HIV prevention counseling at local health clinics.

This form gives more information about the study. You should feel that you understand what the study is about before deciding whether you will participate.

**ABOUT THE STUDY**

The HIV Prevention Trials Network (HPTN) and MU-JHU are doing this study to understand if it is possible for barbers to provide HIV prevention services, like counseling and HIV self-test kits, at barbershops. We also want to know if men like receiving these services at a barbershop and if it helps to increase the number of men getting an HIV test or going to the clinic for HIV prevention or treatment services.

About 250 people will participate in this study from Kalangala District, Uganda. Participants will be in the study for about one year.

1. **This study is testing if barbers can provide HIV prevention services to men.**

Men and boys are less likely to test for HIV and receive treatment compared to women. It can be difficult for men to test and get HIV prevention services for many reasons. Health clinics can be hard to get to or may only be open when people are working during the day. Sometimes men feel judged by health care providers. We want to see if a barber can provide HIV prevention services and if men would like these services. The barber is often a trusted community member and may be able to help men get tested and receive care. The study may show a new way to improve the health of men who find it hard to engage in HIV prevention in other ways.

1. **Barbershops in the study will be placed in one of two groups. Participants will receive services depending on their shop.**

All the barbershops included in the study were randomly put into one of two groups. Random means the study staff did not choose and the barber did not choose. It is like rolling a dice. The difference between the shops is that one group will have trained barbers. These trained barbers will provide clients in the study with HIV education, give them HIV self-test kits, and invite them to group sessions to discuss HIV prevention. The other group of shops will continue to have the standard-of-care HIV prevention services in health facilities. For every two barbershops randomly assigned to have trained barbers, one barbershop will be assigned to provide the standard-of-care. This is called a 2:1 randomization.

If your shop is in the group with trained barbers, you will receive these HIV prevention services from your barber. If your shop is in the other group, you will not receive these services from your barber. All participants will have study visits approximately six months after starting the study. Some participants will also have a visit at 12 months.

**JOINING THE STUDY**

1. **It is your decision whether to participate in the study.**

This consent form gives information about the study that we will discuss together. We will help you understand the form and answer your questions before you sign this form. Once you understand the study, and if you agree to take part, you will be asked to sign your name or make your mark on this form. You will be offered a copy of this form to keep.

Before you learn about the study, it is important that you know the following:

- Your participation is voluntary. You do not have to take part in any of the tests or procedures in the study.
- You may decide not to take part in the study, or you may decide to leave the study at any time without losing your regular medical care. You can also continue to attend your barber for haircuts without being in the study.
- If you decide not to take part in the study, you can still join another study at a later time if there is one available and you qualify.
- You cannot join this study if you are taking part in another study of drugs or medical devices. You are asked to tell the study staff about any other studies you are taking part in or thinking of taking part in. This is very important for your safety.

1. **You must qualify before you can join the study.**

If you decide to join this study, we will first do some tests and collect some information from you to find out if you qualify. These tests and the information collected are described in #6 below. If you do not qualify, you cannot join the study.

1. **We will ask you questions, examine you, and test your blood and urine.**

To find out if you qualify, we will first ask some questions and do some tests. This will happen after you read, discuss, understand, and sign this form. These tasks will take about 30 minutes.

At the Screening Visit, we will:

- Ask you questions about yourself, like your age
- Ask you to answer questions on a computer about your sexual practices
- Ask about where you live and how to contact you
- Talk with you about HIV and ways to protect yourself from getting it. We will also offer you condoms.
- Collect ~10mL (about 2 teaspoons) of blood for HIV and syphilis testing. Some blood will be stored during the study for quality control testing. Quality control testing is used to make sure HIV testing is working the way it should in the laboratory.

Your HIV test results will be available during the visit. You will be contacted about the results of your other tests when they are available. If you test positive for syphilis, you will be provided or referred for treatment.

1. **If you qualify and are interested, you will join the study.**

We will review your test results and the information you provide today. If you have a positive HIV test you will not be eligible for the study, and you will be referred for the appropriate medical care.

If you are eligible for this study and decide to join, you will be asked to continue with today’s visit for an “Enrollment Visit.” If you are unable to continue with the visit today, you may come back to the clinic on another day to finish the Enrollment Visit. We ask that you come for enrollment within two weeks. This visit will last about 2 hours.

During the Enrollment Visit, we will:

- Confirm information already provided
- Ask you questions about yourself, like your use of HIV prevention methods, medical history, and if you are taking any medication.
- Ask you to answer questions on a computer about your sexual practices
- If you report any medical concerns, we will give you a brief physical exam.
- Ask you to provide urine for gonorrhea and chlamydia testing
- Provide treatment if you have symptoms of an STI
- Tell you if your barber is providing the intervention

**BEING IN THE STUDY**

1. **You will have up to two study visits over 12 months.**

If you decide to join the study, you will be asked to come to this clinic six months after your enrollment visit. We may also ask you to come back for a visit twelve months after your enrollment visit.

Each visit will last about 2 hours.

During these study visits, we will:

- Confirm where you live and how to contact you
- Ask you questions about yourself, like your use of HIV prevention methods, updates to your medical history, and if you are taking any new medication.
- Ask you to answer questions on a computer about your sexual practices
- Talk with you about HIV and ways to protect yourself from getting it. We will also offer you condoms.
- If you report any medical concerns, we will give you a brief physical exam.
- Collect ~10 mL (about 2 teaspoons) of blood for HIV testing. Some blood will be stored during the study for quality control testing. Quality control testing is used to make sure HIV testing is working the way it should in the laboratory.
- Ask you to provide urine for gonorrhea and chlamydia testing
- Provide treatment if you have symptoms of an STI

You may have more visits if you are sick or we need to check on your health.

At your final study visit, we will talk with you about the end of the study and when the results of the study will be available.

1. **If you get HIV during the study, we will help you get care and support.**

We will test your blood for HIV during this study. If you get HIV while you are in the study, we will help you find the care and support you need. We will ask you to come back for your study visits after confirming your HIV status. We will examine you and ask you about any medications you are taking. We will also provide you with condoms.

If you attend a barbershop with a trained barber, you can decide if you want the barber to stop providing HIV education and services. You may tell your barber to stop providing HIV services. Or, if you want, study staff can tell them to stop providing HIV services. You will be able to continue with your regular haircut services.

1. **You will continue to receive haircuts from your barber.**

If your barbershop is in the group with trained barbers, we will tell your barber that you have joined the study. We will also ask you to tell your barber that you are in the study. At your haircuts with the barber, they will:

- Provide you with HIV education
- Give you HIV self-test kits to take with you
- Invite you to group sessions that will happen about every two months
- Record the number of times you come for a haircut, receive counseling, and take HIV self-test kits. Your barber will give this information to study staff.

If your barbershop is in the group without trained barbers, you will not receive any of the HIV prevention services from your barber. We will provide you with information about HIV prevention services available to you in the community.

1. **We may ask you to participate in a long discussion about the study**

Some participants will be asked to come for an interview with study staff. At the interview we will ask:

- How you feel about the barbershop-based services
- Your feelings about HIV and HIV prevention services
- About your relationships and sexual behaviors

The interviews require one or two visits and will last around 1 hour. During the interview we will audio record your responses and then later write them down (called a transcript). The written transcript will not include any identifying information. The audio recordings will be destroyed after all analysis is completed.

1. **Use of samples.**

Blood and urine samples will only be used for HIV and STI testing and quality control. Quality control testing is used to make sure HIV testing is working the way it should in the laboratory.

Samples will be destroyed after completion of testing. We will not store any samples after this testing is complete for long-term storage.

**RISKS OF THE STUDY**

1. **There may be risks to being in this study.**

*STUDY PROCEDURES*

Taking blood samples may cause some pain, bruise your arm, or make you feel lightheaded. In rare cases you may faint. There is also a slight chance of infection when blood is drawn. You may be nervous while you are waiting for your HIV test result. If the tests show that you have HIV, you may worry about your health and future. You will receive counseling before and after the test to help address your concerns.

*DISCLOSURE OF PERSONAL INFORMATION*

We will make every effort to protect your confidentiality during the study. However, it is possible that others may learn that you are part of this study and they may think that you are living with HIV or are at increased likelihood of getting HIV. Because of this you could have trouble finding or keeping a job. You could also have problems with your family, friends and community.

If you were told about this study by your barber, it is possible they may think they know your HIV status based on your enrollment in the study or if you decide to stop the study. We will not tell the barber your HIV status. If you attend the group sessions with a trained barber, we cannot guarantee that other participants will keep the discussions private.

We will not tell anyone about your HIV status or if you have a sexually transmitted infection without your permission. We can tell you more about how we will protect your information.

*SENSITIVE QUESTIONS*

The questions we will ask you about your sexual behavior may make you feel uneasy. However, you do not have to answer any question that you do not want to and you can stop answering the questions at any time.

*SOCIAL*

There may also be some social risks to participating in this study. You may feel embarrassed or uncomfortable with some of the questions you will be asked, some of the procedures that will be done, or some of the test results that you will receive. You may also experience stigma as a result of being involved in a study about HIV because people may assume that you are living with HIV. Family or friends may worry, get upset or angry, or assume that you are living that there is a high likelihood of you getting HIV and treat you unfairly as a result.

*OTHER RISKS*

We do not know if there are other risks if you participate in this study. You should report any problems to the researchers immediately.

**BENEFITS OF THE STUDY**

1. **There may be no direct benefit to you by participating in the study.**

Since we do not know if the barbershop-based HIV prevention services help men, there may be no direct benefit to you to being in the study. However, being in the study may still help you in some ways. We will test you for HIV and other sexually transmitted infections throughout this study. The counseling you get from study staff may help you to avoid HIV and other sexually transmitted infections. If you are living with or get HIV, this counseling may help you to learn how to better care for yourself and avoid passing HIV to others. Participants who go to a barber who is delivering the HIV services may benefit from the information provided by the barber or from getting an HIV self-test kit.

If you get HIV, or have another sexually transmitted infection, we will treat you or refer you for care and/or treatment. At every visit you will receive condoms free of charge.

You may not receive any other direct benefit from being in this study; however, you or others in your community may benefit from this study later. The information gathered during this study may help to prevent HIV and other infections. This may be beneficial to you and your community.

**OTHER INFORMATION ABOUT THE STUDY**

1. **We will tell you any new information that may affect your decision to be in the study.**

You will be told any new information learned during this study that might affect your willingness to stay in the study. For example, if information becomes available that shows that the barbershop-based HIV prevention services seem to work very well or do not work very well, you will be told about this. You will also be told when the results of the study may be available, and how to learn about them.

1. **You may be withdrawn from the study without your consent.**

We may take you out of the study at any time without your consent. This may happen if:

- You are unable or unwilling to follow all of the study procedures or instructions.
- The study is stopped or canceled.
- The study staff feels that staying in the study would be harmful to you.
- Other reasons, as decided by the study staff
- If we take you out of the study, we may ask you to come back to the clinic one last time to check your blood and urine and ask you questions

1. **You have other choices if you choose not to be in this study.**

There may be other studies going on here or in the community that you may qualify for. If you wish, we will tell you about other studies that we know about. There also may be other places where you can go for HIV counseling and testing. We will tell you about those places if you wish.

1. **There is no cost to you to be in this study.**

There will be no cost to you for study related visits, physical examinations, laboratory tests, or other procedures.

1. **We will give you [site to insert amount] for each study visit.**

You will receive [*$xx*] for your time, effort, and travel to and from the clinic at each scheduled visit. [*Site to insert information about local reimbursement for the study.]*

1. **We will do our best to protect your private information.**

Every effort will be made to keep your personal information confidential, but we cannot guarantee absolute confidentiality. To keep your information private, your samples will be labeled with a code that can only be traced back to your study clinic. Your name, where you live, and other personal information will be protected by the study clinic. The results of any tests done on these samples will not be included in your health records. You will be identified by a code, and personal information from your records will not be released without your written permission. Any publication of this study will not use your name or identify you personally. Your personal information may be disclosed if required by law.

Study staff will have access to your study records. Your records may also be reviewed, under guidelines of the US Federal Privacy Act, by:

- The Uganda Virus Research Institute (UVRI) Research Ethics Committee
- The Uganda National Council for Science and Technology
- Uganda Ministry of Health (MOH)
- Uganda National Health Research Organization (UNHRO)
- The sponsor of the study (US National Institutes of Health [NIH]), its contractors, and its study monitors
- The US Office for Human Research Protections (OHRP)
- Other local, US, or international regulatory authorities/entities
- The HPTN that is conducting this study

The study staff will also use your personal information, if needed, to verify that you are not taking part in any other research studies. This includes other studies conducted by MU-JHU and studies conducted by other researchers that study staff know about.

A description of this clinical trial will be available on <http://www.ClinicalTrials.gov>. This Web site will not include information that can identify you. At most, the web site will include a summary of the results. You can search this Web site at any time.

1. **If you get sick or injured during the study, contact us immediately.**

It is unlikely that you will be injured as a result of study participation. If you are injured, the study team will give you immediate necessary treatment for your injuries. You will not have to pay for this treatment. If the study team cannot treat your injury, you will be referred to a facility that can treat you. There is no program to pay money or give other forms of compensation for such injuries either through this institution or the US NIH. You do not give up any legal rights by signing this consent form.

1. **Contact us at any time if you have questions or problems.**

If you ever have any questions about the study, or if you have a research-related injury, you should contact *[insert name of the investigator or other study staff]* at *[insert telephone number and/or physical address*].

If you have questions about your rights as a research participant, you should contact [*insert name or title of person on the IRB or other organization appropriate for the site*] at [*insert physical address and telephone number].*

If you have questions about who to contact at the research site, you should contact [*insert name of the investigator or community educator or Community Advisory Board member*] at [*insert physical address and telephone number].*

**SIGNATURE PAGE**

**HPTN 111**

**Feasibility and Acceptability of a Barbershop Based HIV Prevention Initiative Among Heterosexual Men in Kalangala Islands, Uganda: A Cluster Randomized Trial Participant Informed Consent Form**

**Version 1.0**

**18 September 2023**

Screening and Enrollment Consent

If you have read this consent form, or had it read and explained to you, and you understand the information, and you voluntarily agree to join the study, please sign your name or make your mark below. Also, please indicate by providing your initials in the spaces below if you agree to have a study visit outside of the study clinic or participate in an interview.

____ I agree to take part in this study.

_____ I agree to complete study visits at a location outside of the study clinic. Study staff will coordinate with me about an acceptable location.

____ I do not agree to complete study visits at a location outside of the study clinic.

____ I agree to participate in an interview where I will be asked questions about this research, and the interview will be recorded.

____ I do not agree to participate in an interview where I will be asked questions about this research, and the interview will be recorded.

________________________________ _____________________________________

Participant Name (print) Participant Signature and Date

________________________________ _____________________________________

Study Staff Conducting Consent Discussion (print) Study Staff Signature and Date

________________________________ _____________________________________

Witness Name (print) (As appropriate) Witness Signature and Date

Sample Informed Consent (Barber)

**HPTN 111**

**Feasibility and Acceptability of a Barbershop Based HIV Prevention Initiative Among Heterosexual Men in Kalangala Islands, Uganda: A Cluster Randomized Trial**

**Barber Informed Consent Form**

**Version 1.0**

**18 September 2023**

**DAIDS Document ID: 39062**

**Sponsored by:** Division of AIDS, US National Institute of Allergy and Infectious Diseases, US National Institutes of Health.

**PRINCIPAL INVESTIGATOR:** [Insert Name]

**PHONE:** [Insert Number]

**INTRODUCTION**

We are asking you to take part in a research study. Joining this study is voluntary. You may refuse to join, or you may withdraw your consent to be in the study, for any reason. This research study is for barbers who are participating in a barbershop-based program for HIV prevention.

Before you decide whether to join the study, we would like to explain the purpose of the study, the risks and benefits to you, and what is expected of you.

1. **You should know key information about this study before you join.**

Here is a summary of important information about the study:

- This is a research study.
- Your participation in this study is voluntary.
- We are trying to learn if it is possible for barbers to provide HIV prevention services, like education and HIV self-test kits, at barbershops. We also want to know if barbers like providing these services and if it helps to increase the number of men getting an HIV test and going to the clinic for HIV prevention or treatment services.
- We have chosen different barbershops in Kalangala district to be part of this study. Some shops will be randomly chosen to train barbers to provide HIV prevention services. Other shops will be randomly chosen to continue with no HIV prevention services. Depending on which shop you work in, you will either deliver the intervention from your shop or not. All barbers in both groups will help refer clients to the study.
- This study has up to six visits with study staff after today. If you are eligible and want to participate after today, then you will have an initial visit. If you work at a shop chose to deliver the intervention, you will have a visit in approximately 6 and 12 months at the study clinic to talk about your experiences with the program. We will also ask some questions about the program when we come visit you at your shop in approximately 3, 9 and 15 months.
- At each study visit we will ask questions about how you feel about the program and if you like providing HIV prevention services to your clients.
- There are minimal risks to you if you decide to participate in the study. You may be uncomfortable answering questions about your experiences. We will do our best to protect your information, but it is possible other people may learn of your participation in this study. If other people learn about your participation, it may impact whether clients want to attend your shop.
- There may be no direct benefit to you if you decide to participate in the study. However, you may benefit from the information you learn from study staff about HIV prevention.
- If you decide not to participate in the study, you may learn about HIV prevention at local health clinics.

This form gives more information about the study. You should feel that you understand the study before deciding whether you will participate.

**ABOUT THE STUDY**

The HIV Prevention Trials Network (HPTN) and MU-JHU are doing this study to understand if it is possible for barbers to provide HIV prevention services, like education and HIV self-test kits, at barbershops. We also want to know if barbers like providing these services at their shop and if it helps to increase the number of men getting an HIV test or going to the clinic for HIV prevention or treatment services.

About 18 barbers will participate in this study from Kalangala district, Uganda. Barbers will be in the study for approximately 15 months.

1. **This study is testing if barbers can provide HIV prevention services to men.**

Men and boys are less likely to test for HIV and receive treatment compared to women. It can be difficult for men to test and get HIV prevention services for many reasons. Health clinics can be hard to get to or may only be open when men are working during the day. Sometimes men feel judged by health care providers. We want to see if a barber can provide HIV prevention services and if men would like these services. We also want to know if barbers like providing these services. The barber is often a trusted community member and may be able to help men get tested and receive care. The study may show a new way to improve the health of men who find it hard to engage in HIV prevention in other ways.

1. **Barbershops in the study will be placed in one of two groups. Clients in the study will receive services depending on their shop.**

All the barbershops included in the study will be randomly put into one of two groups. Random means the study staff did not choose and the barbers did not choose. It is like rolling a dice. The difference between shops is that one group will have barbershops with trained barbers. These trained barbers will provide clients in the study with HIV education, give them HIV self-test kits, and invite them to group sessions to discuss HIV prevention. The other group of barbershops will not deliver any HIV prevention services. Men at these shops will continue to have the standard-of-care HIV prevention services in health facilities. For every two barbershops randomly assigned to have trained barbers, one barbershop will be assigned to provide the standard-of-care. This is called a 2:1 randomization.

Barbers in both groups will help refer clients to the study. Before you help recruit clients or deliver the services, you will attend a training to learn about the study and the services.

**JOINING THE STUDY**

1. **It is your decision whether to participate in the study.**

This consent form gives information about the study that will be discussed with you. We will help you understand the form and answer your questions before you sign this form. Once you understand the study, and if you agree to take part, you will be asked to sign your name or make your mark on this form. You will be offered a copy of this form to keep.

Before you learn about the study, it is important that you know the following:

- Your participation is voluntary. You do not have to take part in any of the procedures in the study.
- You may decide not to take part in the study, or you may decide to leave the study at any time.
- If you decide not to take part in the study, you can still join another study at a later time if there is one available and you qualify.

**BEING IN THE STUDY**

1. **You will refer clients to study staff. If you are in the group of shops with trained barbers, you will deliver HIV prevention services to your clients who are in the study. You will also have your own study visits.**

If you decide to join the study, both groups of barbers will:

- Be trained on how to keep your client’s confidentiality.
- Be trained on the study and how to recruit clients. Training will take approximately 1-2 days.
- Provide information about your shop and experiences as a barber.
- Provide information about your experience referring clients to the study. You will also be asked to complete a simple checklist that includes the number of clients you refer to the study.

If you are in the group chosen to deliver HIV prevention to clients, you will:

- Be trained on how to provide education and HIV test kits. This extra training will take another 2-3 days.
- Provide peer support groups every two months.
- Complete a simple checklist of the services provided to study participants.
- You will have up to six study visits during the 15 months. These visits will occur approximately quarterly. At some study visits you will be asked to provide information about your experience and opinions about delivering HIV prevention services in this study. We will ask these questions when we visit you at your shop.
- At the visits at 6 and 12 months at the study clinic, we will ask questions in an individual interview. During the interview we will audio record your responses and then later write them down (called a transcript). The written transcript will not include any identifying information. The audio recordings will be destroyed after all analysis is completed.

Each study visit will last about one hour.

**RISKS OF THE STUDY**

1. **There may be risks to being in this study.**

*DISCLOSURE OF PERSONAL INFORMATION*

We will make every effort to protect your confidentiality during the study. However, it is possible that others may learn that you are part of this study, and they may not be comfortable with your participation. Because of this you could have trouble finding or keeping a job or it may mean that some clients do not want to come to your barbershop. You could also have problems with your family, friends, and community. We can tell you more about how we will protect your information.

*SENSITIVE QUESTIONS*

It is possible that the questions we ask you about your experiences will make you feel uneasy. However, you do not have to answer any question that you do not want to and you can stop answering the questions at any time.

*SOCIAL*

There may also be some social risks to participating in this study. You may experience stigma as a result of being involved in a study about HIV. It is possible that clients may not want come for haircut services if they know you are part of this study or associate your shop with HIV and sexually transmitted infections.

**BENEFITS OF THE STUDY**

.

1. **There may be no direct benefit to you by participating in the study.**

There may be no direct benefit to you to being in the study. However, you or others in your community may benefit from this study later. The information gathered during this study may help to prevent HIV and other infections. It is possible that the information you learn during study training will help you understand how to prevent HIV.

**OTHER INFORMATION ABOUT THE STUDY**

1. **We will tell you any new information that may affect your decision to be in the study.**

You will be told any new information learned during this study that might affect your willingness to stay in the study. You will also be told when the results of the study may be available, and how to learn about them.

1. **You may be withdrawn from the study without your consent.**

We may take you out of the study at any time without your consent. This may happen if:

- You are unable or unwilling to follow all of the study procedures or instructions.
- You are unable to refer enough clients to study staff.
- The study is stopped or canceled.
- The study staff feels that staying in the study would be harmful to you.
- Other reasons, as decided by the study staff.

1. **You have other choices if you choose not to be in this study.**

There may be other studies going on here or in the community that you may be eligible for. If you wish, we will tell you about other studies that we know about.

1. **There is no cost to you to be in this study.**

There will be no cost to you for the study visits.

1. **We will give you [*site to insert amount*] for each study visit and up to [*site to insert amount*] for providing the HIV prevention services to your clients.**

You will receive [*$xx*] for your time, effort, and travel for each visit. You will receive up to [*$xx*] each month for your time and effort providing the study services to clients.

1. **We will do our best to protect your private information.**

Every effort will be made to keep your personal information confidential, but we cannot guarantee absolute confidentiality. You will be identified by a code, and personal information from your records will not be released without your written permission. Any publication of this study will not use your name or identify you personally. Your personal information may be disclosed if required by law.

Study staff will have access to your study records. Your records may also be reviewed, under guidelines of the US Federal Privacy Act, by:

- The Uganda Virus Research Institute (UVRI) Research Ethics Committee
- The Uganda National Council for Science and Technology
- Uganda Ministry of Health (MOH)
- Uganda National Health Research Organization (UNHRO)
- The sponsor of the study (US National Institutes of Health [NIH]), its contractors, and its study monitors
- The US Office for Human Research Protections (OHRP)
- Other local, US, or international regulatory authorities/entities
- The HPTN that is conducting this study

A description of this clinical trial will be available on <http://www.ClinicalTrials.gov>. This Web site will not include information that can identify you. At most, the Web site will include a summary of the results. You can search this Web site at any time.

1. **If you get injured during the study, contact us immediately.**

It is unlikely that you will be injured as a result of study participation. If you are injured, the study team will give you immediate necessary treatment for your injuries. You will not have to pay for this treatment. You will be told where you can get additional treatment for your injuries. There is no program to pay money or give other forms of compensation for such injuries either through this institution or the US NIH. You do not give up any legal rights by signing this consent form.

1. **Contact us at any time if you have questions or problems.**

If you ever have any questions about the study, or if you have a research-related injury, you should contact [*insert name of the investigator or other study staff] at [insert telephone number and/or physical address*].

If you have questions about your rights as a research participant, you should contact [*insert name or title of person on the IRB or other organization appropriate for the site*] at [*insert physical address and telephone number*].

If you have questions about who to contact at the research site, you should contact [*insert name of the investigator or community educator or Community Advisory Board member*] at [*insert physical address and telephone number].*

**SIGNATURE PAGE**

**HPTN 111**

**Feasibility and Acceptability of a Barbershop Based HIV Prevention Initiative Among Heterosexual Men in Kalangala Islands, Uganda: A Cluster Randomized Trial Participant Informed Consent Form**

**Version 1.0**

**18 September 2023**

Barber Consent

If you have read this consent form, or had it read and explained to you, and you understand the information, and you voluntarily agree to join the study, please sign your name or make your mark below.

____ I agree to take part in this study.

________________________________ _____________________________________

Participant Name (print) Participant Signature and Date

________________________________ _____________________________________

Study Staff Conducting Consent Discussion (print) Study Staff Signature and Date

________________________________ _____________________________________

Witness Name (print) (As appropriate) Witness Signature and Date
