# Supplementary material for: Feasibility and Acceptability of Barbershop-Based HIV Prevention Among Heterosexual Men in Kalangala Islands, Uganda: Protocol for a Cluster Randomized Trial (HPTN 111)
Source: JMIR Res Protoc. 2026 Apr 17;15:e87612. doi: 10.2196/87612 (PMC13135168; doi:10.2196/87612)
Supplement: Multimedia Appendix 4 [file resprot_v15i1e87612_app4.docx]

**Supplemental Appendix II: Schedule of Events for Participants**

|  | Screening | Enrollment | Week 26 | Week 52 |
| --- | --- | --- | --- | --- |
| **Administrative and Behavioral Procedures** | | | | |
| Informed Consent | X |  |  |  |
| Locator information | X | X | X | X |
| Demographic information | X | X |  |  |
| Social impacts assessment |  | X | X | X |
| Socio-behavioral assessment | X | X | X | X |
| Disclose study group (intervention or control) |  | X |  |  |
| Intervention acceptability assessment |  | X | X | X |
| HIV self-testing assessment |  |  | X | X |
| HIV counseling and testing | X |  | X | X |
| Provide information about recommended HIV testing schedule |  | X | X | X |
| Offer condoms | X | X | X | X |
| In-depth interviews^1,2^ |  |  | X | X |
| **Clinical Procedures** | | | | |
| Complete medical history |  | X |  |  |
| Targeted medical history |  |  | X | X |
| Symptom directed physical exam |  | X | X | X |
| Concomitant drug use |  | X | X | X |
| Blood collection | X |  | X | X |
| Urine collection |  | X | X | X |
| HIV prevention services or HIV care referral | X | X | X | X |
| STI treatment^3^ | X | X | X | X |
| **Laboratory Procedures** | | | | |
| HIV diagnostic testing^3^ | X |  | X | X |
| STI testing: blood for syphilis | X |  | X^4^ | X^4^ |
| STI testing: urine NAAT for gonorrhea and chlamydia |  | X | X | X |
| Blood storage for LL QC |  |  | X | X |

^1^Intervention group only

^2^ Subset of men only

^3^ HIV RNA testing required if participant has acute symptoms of HIV

^4^ If clinically indicated
