# Supplementary material for: Feasibility and Acceptability of Barbershop-Based HIV Prevention Among Heterosexual Men in Kalangala Islands, Uganda: Protocol for a Cluster Randomized Trial (HPTN 111)
Source: JMIR Res Protoc. 2026 Apr 17;15:e87612. doi: 10.2196/87612 (PMC13135168; doi:10.2196/87612)
Supplement: Multimedia Appendix 5 [file resprot_v15i1e87612_app5.docx]

**Supplemental Appendix III: Schedule of Events for Barbers**

|  | Study Initiation | Weeks 13/39/65 | | Weeks 26/52 |
| --- | --- | --- | --- | --- |
| **Administrative Procedures** | | | | |
| Informed Consent | X |  | |  |
| Memorandum of understanding | X |  | |  |
| Locator information | X |  | |  |
| Collect barber demographics | X |  | |  |
| Collect barbershop details | X |  | |  |
| Training on study and recruitment procedures | X |  | |  |
| Training on barbershop-based intervention procedures^1^ | X |  | |  |
| Social impacts assessment^2^ |  | X | | X |
| Intervention acceptability and feasibility assessment^1^ |  | X | | X |
| In-depth interviews^1^ |  |  | | X |
|  | | | | |
|  | During client-visits to the barbershop | | Every two months | |
| **Barber-led Procedures** |  | |  | |
| Refer clients for study screening/enrollment | X | |  | |
| Record number of clients referred to study | X | |  | |
| Provide HIV education^1^ | X | |  | |
| Provide HIVST kits^1^ | X | |  | |
| Record number of HIVST kits distributed and services provided^1^ | X | | X | |
| HIV prevention services referral^1^ | X | |  | |
| Offer condoms^1^ | X | |  | |
| Lead group sessions^1^ |  | | X | |

^1^ Intervention barbers only

^2^ During enrollment phase for control barbers and during enrollment and follow-up for intervention barbers
