# Supplementary material for: Feasibility and Acceptability of Barbershop-Based HIV Prevention Among Heterosexual Men in Kalangala Islands, Uganda: Protocol for a Cluster Randomized Trial (HPTN 111)
Source: JMIR Res Protoc. 2026 Apr 17;15:e87612. doi: 10.2196/87612 (PMC13135168; doi:10.2196/87612)
Supplement: Multimedia Appendix 6 [file resprot_v15i1e87612_app6.pdf]

HPTN 111 TRIM Study

In-Depth Interview (IDI) Topic Guide for Barbers

INSTRUCTIONS for the Interviewer: How to use the IDI Guide

1. Section topics are in shaded in gray and **bolded**.
2. *Instructions/suggestions to interviewer are in italics and [brackets].*
3. Not ALL questions need to be asked. It is up to the interviewer's discretion if a question should be skipped if the participant has already provided a response to the question earlier in the interview. Please ensure that by the end of the interview, all the topics and key themes have been covered.
4. Purpose statements should be considered notes to the interviewer and are not meant to be read aloud. They explain the reason for asking that question or set of questions in order to provide more context to the interviewer who can then rephrase in her own words or clarify to the participant as necessary.
5. There are two levels of questions:
   a. Primary interview questions: appear in **bold** text. They address the topics that you as the interviewer should ask and discuss with participants. You are not required to read them verbatim, but they are written to ensure some consistency across IDIs.
   b. Probing topics are indicated with a bullet. If you find that the participant does not provide much information in response to the primary question, these probing topics may be used to encourage further discussion. Probes with the words "*KEY PROBE*" written before it are probes that are the most important to try to address. Depending on what has already been discussed, and the IDI context, you may or may not ask the rest of the probes.
6. Words found in (parentheses) are meant to provide wording options to interviewers to fit various situations. For example, they often provide a present or past tense verb.
7. The IDI guide is not meant to be used to take notes. Rather, you should use the separate notes form, where you will also insert your initials, the participant's PTID, as well as the date, start and end time of the interview.

Before starting the IDI, confirm the participant(s) are willing to proceed with the interview.

APPROVED
EFFECTIVE ★ 0 2 NOV 2023 — EXPIRY 0 2 NOV 2024 ★
JOINT CLINICAL RESEARCH CENTRE REC

[*Start Recorder and Read Introduction*]: **My name is __________________. Thank you again for your willingness to be in this study. The main goal of this discussion is to better understand your experiences with providing HIV self-test kits to your clients at your barbershop. I want to remind you that there are no right or wrong answers, and what we discuss here will be kept confidential; we will not share your personal information or responses with anyone outside of the study.**

If during our discussion, there are issues or concerns that you would like to talk about, feel free to bring them up; I will take note of them and answer them directly after the interview. If I cannot answer them, I can refer you to someone who may be able to help. Before we start, can you confirm for the recorder that you are still willing to participate in the interview? [*Wait for oral confirmation to begin*].

Theme: Warm-up (~5 minutes)
Purpose: To warm-up the participants (barber) to the qualitative interview

1. **Tell me about how business is going on in your Barbershop?**
   - How has business been in the past 3 months?
   - Tell me about the type and number of customers you get at your barbershop.

Theme: Experience taking part in the Study
Purpose: To explore Barber's experience of taking part in the study including providing HIV self-test kits, HIV Status neutral information and conducting support group meetings.

2. **Tell me your experience providing HIV self-test kits/HIV Status neutral information/ conducting support group meetings? Ask for each intervention separately, do not combine them.**
   Possible probing topics:
   - What did or didn't you like about providing HIV self-test kits/HIV Status neutral information/conducting support group meetings?
   - How prepared were you to provide these interventions to your customers in the barber shop?
   - Mention any advantages or challenges you found implementing these interventions.
   - What could be done to support you to implement this implementation?

Theme: Interest in HIV-self test kits
Purpose: To understand men's interest in and motivation to use a HIV-self test kits delivered by a Barber in the Barbershop.

APPROVED
EFFECTIVE 02 NOV 2023 EXPIRY 02 NOV 2024
JOINT CLINICAL RESEARCH CENTRE REC

3. **Let's talk now about HIV Self-Test Kits and their delivery by Barbers in the Barbershop. First, what are some of the reasons why men want to use HIV Self-Test Kits?**
   Possible probing topics:
   - What do you think was the primary reason or motivation for men to use/access HIV Self-Test Kits? Explain.
   - What are some of the things that influenced Men's decision to use (or not to use) the HIV Self-Test Kit?
   - What did the Men say about their experience using the HIV Self-Test Kit?
   - What are some of the things that may happen in men's lives in the future that would influence their intention to use a HIV Self-Test Kit?
   - What are reasons men give for not wanting to use a HIV Self-Test Kit?

4. **How easy or difficult was it for Men to use the HIV Self-Test Kit? Explain.**
   Possible probing topics:

- What made it difficult and how can this be addressed?
- What made it easy?

Theme: Barbershop intervention and HIV self-test kits

Now let's talk about your experience being in this study, this includes your experience with providing HIV self-test kits to your clients at the Barbershop, status neutral information and the support group meetings held by the Barber.

5. **How did you feel about providing HIV self-test kits to your clients [men who came to cut their hair in your barbershop]?**

   Possible probing topics:
   Probe for positives and negative feelings about providing the HIV self-test kits to your clients?
   - What worked and what did not work?
   - If there are any negative feelings or barriers to providing the intervention, how can these be addressed?
   - How different was it to provide the HIV self-test kits to your clients at your Barbershop compared to getting them from the health center/Hospital?
   - What are some of the things that the men wanted you as their Barber to do, which may have influenced their use of the HIV Self-Test Kit
   - How willing were you to share information about the interventions including HIV self-test from the Barbershop with your friends/partner/family members?

APPROVED
EFFECTIVE 02 NOV 2023 ★ EXPIRY 02 NOV 2024 ★
JOINT CLINICAL RESEARCH CENTRE REC

6. **Now let's talk about specific conversations you've had recently with the men [your clients] regarding HIV prevention. Think about the last conversation you had about [HIV prevention/support group discussions** *(Note: Interviewer to ask about HIV prevention education in the Barbershop then the discussions during the support groups or vice versa, do not combine the two)*]**. Tell me about it.**

   Possible probing topics:
   - How did the conversations about [HIV prevention/Support Group Discussions] come up in the Barbershop/community location?
   - What are some of the circumstances that prompted a conversation about HIV prevention?
   - What information did you give you during the HIV prevention sessions in the barber shop/During the support group meetings? [*Probe for Topics of discussion*]
   - What other activities could be done to help men access HIV self-test kits in the Barbershop?

7. **What are Men's preferences for where to get HIV Self-Test kits? Explain?**

   Possible probing topics:
   - Where would men prefer to get the HIV Self-Test Kits? Health Center/Barbershop/Other locations
   - What needs to be available for Men to access HIV self-Test Kits from the Barbershop?
   - What are your thoughts about men getting HIV Self-Test Kits from the Barbershop? Will other men be willing to get HIV Self-Test Kits from their Barbershops? Explain.

Theme: Community perceptions, rumors and myths

Purpose: To gain insight on community perceptions, rumors and myths that may influence preference for and interest in HIV Self-Test Kits including the Barbershop Intervention.

8. **Tell me about the HIV Self-Testing in your community.**
   Possible probing topics:
   - What are some of the things that the community says about HIV self-testing?
   - How may these influence Men's choice of HIV Self-Testing?
   - How are these likely to influence future use of HIV Self-Tests?
   - What are some of the strategies that can be employed to address these?
   - What are the things that the community may say about delivery of HIV Self-Test Kits in the Barbershop? How can these be addressed?

Theme: Wrap Up and Closing Remarks

13. **Thank you for taking the time to talk to me and share your opinions. We truly appreciate your willingness to participate and discuss your experience with us. Before we end, I want to give you the chance to tell us anything else you would like to share about HIV Self-Testing and the Barbershop Intervention.**

14. **Do you have any questions for me?**

APPROVED
EFFECTIVE EXPIRY
★ 0 2 NOV 2023 0 2 NOV 2024 ★
JOINT CLINICAL RESEARCH CENTRE
REC
