# Supplementary material for: Feasibility and Acceptability of Barbershop-Based HIV Prevention Among Heterosexual Men in Kalangala Islands, Uganda: Protocol for a Cluster Randomized Trial (HPTN 111)
Source: JMIR Res Protoc. 2026 Apr 17;15:e87612. doi: 10.2196/87612 (PMC13135168; doi:10.2196/87612)
Supplement: Multimedia Appendix 7 [file resprot_v15i1e87612_app7.pdf]

Specific survey endpoints

This appendix describes the specific endpoints used to assess the feasibility and acceptability of the barbershop-based HIV prevention initiative.

Both barbers and participants answer a large number of questions relevant to feasibility and acceptability. For all these endpoints, the general objective is to evaluate the feasibility and acceptability of the barbershop-based HIV prevention intervention. The intervention has three main components: HIV education and counseling, HIV self-test kits, and barber-led peer group sessions. Participants can choose which components to engage with and how frequently they visit the barber. Although the intervention was designed with the idea that people will receive all three components, depending on their availability and preference, participants randomized to the intervention arm may choose not to receive some or all components. In addition to choosing which components to engage with, participants also decide how frequently they want to receive education and counseling, self-test kits, and attend peer group sessions. Summarizing the feasibility/acceptability across all participants in the intervention arm will reflect an average intervention effect across people who have varying levels of engagement with the intervention.

The primary estimands for participants are the average feasibility/acceptability scores for a typical client from a typical shop delivering barbershop based HIV prevention services. Survey responses about feasibility and acceptability (answered at Week 26 and Week 52) are converted into three separate scores and averaged across visits. Average feasibility and acceptability scores weight villages equally and weight participants equally within villages. See below for details on how scores are calculated.

The primary estimands for barbers are the average feasibility/acceptability scores for a typical barber from a typical shop delivering barbershop based HIV prevention services. Survey responses about feasibility and acceptability (answered at quarterly visits) are converted into two separate scores and averaged across visits. Average feasibility and acceptability scores weight villages equally and weight barbers equally within villages. See below for details on how scores are calculated.

Calculation of scores

Multiple endpoints described below involve scores to combine a group of related questions about feasibility or acceptability. Questions that inform these scores comprise of a statement or question, with a five-level Likert scale response. For each question answers will be scored from -2 to 2 so that the answer that is least supportive of the intervention will be given a score of -2, and the answer that is most supportive of the intervention will be given a score of 2. To combine multiple questions into a score for a particular person at a particular time point, we will average the responses to all the questions. As a result, all scores will have a possible range from -2 (least supportive) to 2 (most supportive). Note that the scores are computed for each individual (barber or participant) at each visit. To get an overall score for each individual, we will average scores across all visits. To get an overall score for each village or shop, we will average scores across all individuals within that village or shop. For example, suppose y_{ijk} is a feasibility or acceptability score from participant *j* in village *i* at time *k*. We are interested in the average score $\bar{y} = \frac{1}{12}\sum_i \frac{1}{N_i}\sum_j \frac{1}{N_j}\sum_k y_{ijk}$, which first takes the average over the $k = 1, \dots, N_j$ visits for participant *j*, then takes the average over the $j = 1, \dots, N_i$ participants within village *i*, then takes the average over the *i=1,…,12* villages randomized to the intervention. This is a village-level summary of scores across the duration of the trial. Note that the number of visits may differ between individuals, but this weights individuals equally within village regardless.

Similarly, the number of individuals may differ between villages, but this weights villages equally regardless. If there were one shop per village, this would be equivalent to equally weighting shops. However, in the case of replacement shops within villages, this method weights shops within villages by the number of participants, so that shops which enrolled or serviced more customers within a single village have more influence on the village-level average.

Interpretation of scores

The table below provides a guide on interpreting the feasibility and acceptability scores:

Score	Interpretation
2	Most favorable: all the questions were answered with the option reflecting the best possible feasibility/acceptability
1	Favorable
0	Neutral: there were a mix of favorable and unfavorable responses, indicating questionable feasibility/acceptability
-1	Unfavorable
-2	Least favorable: all the questions were answered with the option reflecting the worst possible feasibility/acceptability

Domains

Scores will be calculated for five different domains of questions. The domains and the questions they contain are listed below.

Participant-assessed feasibility of barber qualities

1) The barber was knowledgeable about HIV and prevention. Strongly disagree, Disagree, Neutral, Agree, Strongly agree
2) The barber was knowledgeable about HIV self-testing. Strongly disagree, Disagree, Neutral, Agree, Strongly agree
3) The barber was non-judgmental and listened to my thoughts. Strongly disagree, Disagree, Neutral, Agree, Strongly agree
4) I felt that the barber-led group sessions provided a safe and comfortable space to discuss HIV-related topics. Strongly disagree, Disagree, Neutral, Agree, Strongly agree *(note – participants who did not attend a group session will be given a score of zero for this question)*

Participant-assessed feasibility of intervention experience

1) I feel that I have more knowledge about general HIV prevention and treatment services after talking with my barber. Strongly disagree, Disagree, Neutral, Agree, Strongly agree
2) I feel that I have more knowledge about HIV testing after talking with my barber. Strongly disagree, Disagree, Neutral, Agree, Strongly agree
3) I feel that I have more knowledge about PrEP after talking with my barber. Strongly disagree, Disagree, Neutral, Agree, Strongly agree
4) I feel that I have more knowledge about PEP after talking with my barber. Strongly disagree, Disagree, Neutral, Agree, Strongly agree

5) I feel that I have more knowledge about safe male circumcision after talking with my barber. Strongly disagree, Disagree, Neutral, Agree, Strongly agree

Participant-assessed acceptability of the intervention

1) How satisfied are you with the HIV information provided by your barber? Very satisfied, Satisfied, Neutral, Dissatisfied, Very dissatisfied
2) How comfortable were you discussing HIV and other health related topics with your barber? Very comfortable, Somewhat comfortable, Neutral, Somewhat uncomfortable, Very uncomfortable
3) I am more comfortable seeking HIV prevention (i.e., testing) and treatment services after talking with my barber. Strongly disagree, Disagree, Neutral, Agree, Strongly agree
4) I felt that talking to my barber about HIV topics and receiving a haircut was too time consuming. Strongly disagree, Disagree, Neutral, Agree, Strongly agree
5) I felt uncomfortable with the idea of taking an HIV self-test kit from my barber. Strongly disagree, Disagree, Neutral, Agree, Strongly agree
6) I liked being able to get an HIV self-test kit from my barber. Strongly disagree, Disagree, Neutral, Agree, Strongly agree
7) I worried about other people in the shop hearing my barber talking to me about HIV. Strongly disagree, Disagree, Neutral, Agree, Strongly agree
8) I worried that I might experience stigma going to the barber who talks about HIV. Strongly disagree, Disagree, Neutral, Agree, Strongly agree
9) Talking with my barber has allowed me to think positively about seeking and/or using HIV prevention (i.e., PrEP and PEP) and treatment (i.e., ART) medications. Strongly disagree, Disagree, Neutral, Agree, Strongly agree
10) I felt that the barber-led group sessions were useful in improving my knowledge about HIV prevention and treatment services. Strongly disagree, Disagree, Neutral, Agree, Strongly agree *(note – participants who did not attend a group session will be given a score of zero for this question)*

Barber-assessed feasibility of intervention delivery

1) How easy was it to provide HIV prevention information to your clients while giving a haircut? Very easy, Somewhat easy, It was not easy or hard, Somewhat difficult, Very difficult
2) How comfortable were you discussing HIV and other health related topics with your clients? Very comfortable, Somewhat comfortable, Neutral, Somewhat uncomfortable, Very uncomfortable
3) I felt comfortable providing HIV prevention information to my clients. Strongly disagree, Disagree, Neutral, Agree, Strongly agree
4) I felt uncomfortable with the idea of giving HIV self-test kits to my clients. Strongly disagree, Disagree, Neutral, Agree, Strongly agree
5) I was uncomfortable talking about HIV with a client when there were other clients waiting in the shop. Strongly disagree, Disagree, Neutral, Agree, Strongly agree
6) I felt comfortable providing information about HIV prevention and treatment services to my clients. Strongly disagree, Disagree, Neutral, Agree, Strongly agree
7) I felt comfortable providing information about HIV testing to my clients. Strongly disagree, Disagree, Neutral, Agree, Strongly agree

8) I felt comfortable providing information about PrEP to my clients. Strongly disagree, Disagree, Neutral, Agree, Strongly agree
9) I felt comfortable providing information about PEP to my clients. Strongly disagree, Disagree, Neutral, Agree, Strongly agree
10) I felt comfortable providing information about safe male circumcision to my clients. Strongly disagree, Disagree, Neutral, Agree, Strongly agree
11) I felt that I was able to answer the questions my clients had about HIV. Strongly disagree, Disagree, Neutral, Agree, Strongly agree
12) I felt comfortable leading the group sessions with my clients. Strongly disagree, Disagree, Neutral, Agree, Strongly agree *(note – barbers who did not lead a group session will be given a score of zero for this question)*
13) I felt knowledgeable about the topics we discussed during the group session. Strongly disagree, Disagree, Neutral, Agree, Strongly agree *(note – barbers who did not lead a group session will be given a score of zero for this question)*
14) I felt confident leading a group of my clients in discussing HIV information. Strongly disagree, Disagree, Neutral, Agree, Strongly agree *(note – barbers who did not lead a group session will be given a score of zero for this question)*

Barber-assessed acceptability of intervention delivery

1) I felt that my clients appreciated discussing HIV prevention information with me. Strongly disagree, Disagree, Neutral, Agree, Strongly agree
2) I like being able to give HIV self-test kits to my clients. Strongly disagree, Disagree, Neutral, Agree, Strongly agree
3) I worried about people not wanting to come to my shop because they don't want to hear about HIV. Strongly disagree, Disagree, Neutral, Agree, Strongly agree
4) I enjoyed providing HIV information and discussing other health related topics with my clients. Strongly disagree, Disagree, Neutral, Agree, Strongly agree
5) I felt that the barber-led group sessions were useful for my clients to learn more about HIV. Strongly disagree, Disagree, Neutral, Agree, Strongly agree *(note – barbers who did not lead a group session will be given a score of zero for this question)*
6) I felt that my clients appreciated attending the barber-led group sessions. Strongly disagree, Disagree, Neutral, Agree, Strongly agree *(note – barbers who did not lead a group session will be given a score of zero for this question)*
