# Supplementary material for: Feasibility and Acceptability of Barbershop-Based HIV Prevention Among Heterosexual Men in Kalangala Islands, Uganda: Protocol for a Cluster Randomized Trial (HPTN 111)
Source: JMIR Res Protoc. 2026 Apr 17;15:e87612. doi: 10.2196/87612 (PMC13135168; doi:10.2196/87612)
Supplement: Multimedia Appendix 8 [file resprot_v15i1e87612_app8.pdf]

HPTN 111: **Feasibility and Acceptability of a Barbershop Based HIV Prevention Initiative Among Heterosexual Men in Kalangala Islands, Uganda: A Cluster Randomized Trial**
SRC DRAFT Version 0.3
15 June 2023

SRC Reviewer Comments
SRC Re-Review Date: 21 June 2023
Response Date: 30 June 2023

Primary Review Group:

- SRC Chair: Sten Vermund (sten.vermund@yale.edu)
- SDMC statistician: Sayan Dasgupta (sdasgup2@fredhutch.org)
- *Ad hoc* scientific reviewer: Joseph Kagaayi (jkagaayi@musph.ac.ug)
- NIH representative: Dianne Rausch (drausch@mail.nih.gov)

Invited Observers:

- HPTN LOC: Caitlin Scoville, Bethany Coombs

Protocol Chairs: Zubair Lukyamuzi, Brenda Gati Mirembe

Action:

Return to the protocol team for revision and resubmission to the SRC

Note: Only Drs. Vermund and Rausch will be reviewing the next protocol draft on behalf of the SRC that we hope will be complete and ready for PSRC review.

==

The SRC subgroup reviewing the revised protocol (V0.3, 15 June 2023) was pleased to see substantial clarifications from the protocol team as to the nature of the barber intervention, alongside a host of improvements in design, detail, and streamlining. Nonetheless, we believe additional issues must be addressed before the PSRC can be approached with a final protocol.

Issues:

1. P. 12, Schema: At the 26- and 52-week visits, both intervention and standard of care (SOC) groups will received both HIV counseling and testing and also "HIV risk reduction counseling and referral". The SRC subgroup is concerned that this distorts the intention of the trial, namely the barber intervention vs. the SOC, since both groups will receive this intensive counseling, an intervention in its own right. We urge a modification that the 26- and 52-week visit receive only the counseling needed in HIV testing, and not any additional counseling. In fact, one might have supervised HIV self-testing at 26 and 52 weeks along with the educational material within the test kit as the only counseling offered, to try to mimic real world circumstances. Otherwise, the risk of a substantial bias towards the null hypothesis threatens the integrity of the experiment.

   Protocol Team Response:

   Thank you for this feedback. We agree and have updated the protocol (schema, Sections 5.2, 5.3) to clarify that all participants will receive basic HIV testing and counseling at the 26- and 52- week study

visits. The counseling provided at visits will follow the provider-initiated HIV testing and counseling (PITC) model which is a standard of care HIV testing strategy recommended by Ugandan national guidelines. PITC involves giving the client information about the risk of HIV from exposures, the potential outcomes of HIV testing, and linkage to post-test services based on outcome of the test. For instance, those with HIV negative results should be linked to HIV prevention services. Participants in the intervention group will receive further information about HIV risk-reduction, available HIV prevention services (e.g. PrEP, PEP, VMMC), and linkage to HIV prevention services from the barber.

2. 2.1: HIV self-testing as documented by the personal assertion of the participant is an unvalidated outcome; hence, the second of the principal outcomes should be considered a secondary outcome rather than a primary outcome. If validation is contemplated via a photo of the self-test result, then one must assure that all participants have a cell phone camera and the ability to convey the results. Even then, one will not know that the test is actually that of the volunteer.

   Protocol Team Response:

   We agree that it would be challenging to collect a validated outcome for self-initiated HIV testing between study visits. We will continue to ask for participants to call study staff when they test and, for that those are able, to send pictures or bring used test kits to the study clinic. However, we recognize the limitations in these methods of reporting. Thus, we have now included this objective as a secondary objective (Protocol Sections 2.0, 8.1).

3. 2.2: The first secondary objective is more suitable as an "exploratory outcome" since STI assessment may be limited in the islands and incidence in a short 6 to 12-month time period will not be adequate to ensure that STI differences will be a properly powered element of this study.

   Protocol Team Response:

   We agree that the study may not be well suited to detect and diagnose incident STIs due to the small number of study visits and limited assessments. We have now included STI incidence as an exploratory objective (Protocol Sections 2.0, 8.1).

4. 3.1.1: Criteria for barber and barbershop eligibility are too complex and restrictive. Can elements that are essential be listed, rather than ones that are desirable? Perhaps a barbershop open for at least six months and a barbershop with at least 15 weekly clients is adequate for eligibility, for example. The requirement that "local stakeholders" (vague, who exactly) testimonials be favorable seems vague and challenging.

   Protocol Team Response:

Thank you for this feedback. We have reviewed and revised the barbershop/barber criteria within the protocol (Section 3.1.1) as follows:
Barbershops:

- Adequate space to deliver the intervention to clients (e.g. space to store HIV self-test kits, laminated counseling sheets, lockable cabinet to store de-identified checklists, etc.)
- Have existed as a shop for ≥ six months
- Have a customer base of ≥ 15 clients per week
- A radius of ≥ 5km maintained between other participating shops

Barbers:

- Barber must have at least a primary level of education
- Barber must be willing and able to participate in the study, recruit participants, and deliver the intervention if their shop is randomized to the intervention group

5. 3.1.1: The >5 km separation between participating barbershops is easily understood methodologically but is complex logistically. How many islands will be included, most likely? Will they be proximate or distant from each other. Might you have to use 12 islands to meet this criterion? We would like to be reassured that the logistics are feasible; otherwise perhaps shoreline villages should be considered for inclusion, for logistical reasons.

Protocol Team Response:

Additional information has been included in Protocol Section 3.1 to indicate that a maximum of 4 islands will be included. Preliminary mapping and data collection on the villages/towns, distances between them, number of barbershops within the village, and ease of reaching the village has been completed by the study team. With this information, we anticipate being able to select 15 barbershops from the largest island, Bugala Island. This island has 18 villages/towns that have potentially eligible barbershops and we will plan to select first from this island. If we are unable to include 15 shops from this island, we will choose shops from an additional 3 islands.

6. 4.1 and 8.3: In the responses to the SRC, the minimal sample size is 180 (12*15) and the maximum is 250. Since this is still unclear in the protocol where it seems that "approximately 250" is the sample size, but 12*15 is listed elsewhere as the sample size, this requires a rewrite within the protocol for clarity.

Protocol Team Response:

We have clarified this within the text of the protocol (Sections 3.0, 8.3). The protocol is designed to recruit a minimum of 12 men from each shop (minimum of 180 total) and continue enrollment up to 250, as funding allows. If the time limit for recruiting men that will have full follow-up of 52 weeks is reached (3 months for recruitment) and fewer than 250 men have been recruited, recruitment may continue until six months have passed or up to 250 men have been enrolled. Men enrolled after the first three months will be followed for 26 weeks.

Where a single number is included for the sample size, we have indicated that up to 250 men will be enrolled.

7. 5.2: The subgroup does not recommend ACASI since its development, programming, and recording will be time consuming and will delay the protocol launch, we believe.

Protocol Team Response:

We appreciate the concern regarding timelines for developing the ACASI. The protocol team feels that ACASI will provide the best opportunity to receive accurate responses regarding sexual behavior in this population. ACASI has been successfully used by protocol team members in other studies and we perceive great benefit to this method.

We have been able to consult with the SDMC regarding the timeline for finalizing an ACASI survey. In order to streamline the process, the protocol team will identify a small number of the most pertinent questions to be delivered via ACASI and will finalize the questionnaire content in conjunction with protocol development. The SDMC will need up to 16 weeks to complete final testing of the system, which in this case will align with the finalization of the protocol and study specific training. Therefore, we believe it will be feasible to have the ACASI ready by first participant enrollment. We will monitor timelines closely and revert to interview-administered questionnaires or locally developed ACASI if ACASI timelines with the SDMC might contribute to study delay.

8. 5.5: Although STIs will be addressed, 5.5. remains devoid of any diagnostic or syndromic diagnostic details.

Protocol Team Response:

We have updated the protocol to further address both diagnostic and syndromic management of STIs in Sections 5.5 and 9.1. NAAT testing will be used for GC/CT and syphilis testing will be done using RPR, followed by TPHA if the RPR is positive (Section 9.1). Syndromic management will follow local Ugandan guidelines (Section 5.5).

9. 8.6.4: An additional rationale for eschewing stopping rules is the minimal risk of the intervention.

Protocol Team Response:

Thank you for noting this. We have included this rationale within the protocol (Section 8.6.4).

10. 1.4: There continues to be little information about formative research that support evidence of trusted relationships between barbers and their clients. The team's preliminary conversations with barbers and customers are not strong evidence compared to published studies.

Protocol Team Response:

We appreciate this comment and concern for the basis of this study. This pilot study was designed to explore if a barbershop-based HIV prevention initiative is acceptable and feasible in Uganda and we aim to contribute to published studies on this topic. Based on our findings, we hope to conduct

additional full-scale studies on the effectiveness of the intervention and implementation on a wider scale.

We were inspired by similar studies for health-related interventions in barbershops in the United States that successfully leveraged these barber-client relationships. While we acknowledge that the context is different in the United States compared to Uganda, we are hopeful based on our preliminary conversations that there is enough interest from both the barbers and clients to make this pilot study a success in understanding the acceptability and feasibility of the intervention.

11. 6.0: The protocol does not adequately address the burden on barbers, how it will be mitigated, and exactly how the intervention will work within the barbershop.

Protocol Team Response:

We agree that it is important to minimize the burden that barbers may face in recruiting participants and delivering this intervention. Barbers will be compensated for their time during study trainings, participant recruitment, completion of checklists and questionnaires, and delivery of the intervention (Section 10.5). They will also be compensated for their time during questionnaire completion and in-depth interviews.

Details about the delivery of the intervention are included in Section 6.0.

12. 4.0 We would like further information and reassurance that men will continue to go to the same barber once enrolled, despite the mobility of this population.

Protocol Team Response:

The barber-client relationship is based on the fact the most men use the same barber over time as it has been reported in previous studies (see section 1.4, paragraph 2). Although the island population is occasionally mobile, we shall minimize this risk by recruiting participants who are regular customers at the participating shop (Section 4.1.1).

While we expect most men to stay customers at the barbershop from where they were recruited, we acknowledge that men may change barbershops during the study or visit a different barber occasionally if they are temporarily away from their primary barber. We will record any changes in barbershop in the study data to understand the mobility of this population (Section 5.6). If a participant in the intervention group relocates permanently to another study area, he will be linked to a study intervention barbershop in their new area and continue with regular study follow-up (Section 5.8). We will record the frequency of interaction with the barber to identify and acknowledge men who may occasionally travel to other islands/locations temporarily due to changes in fishing patterns. The study will provide opportunity to understand any mobility related challenges that might affect the feasibility of the intervention in the island setting.

General comments:

1. The investigators may want to think about assessing for contamination. This could involve simple questions for the control group participants asking if they attended a barbershop other than the

barbershop where they were recruited and whether they heard any HIV education messages from their barber.

Protocol Team Response:

Thank you for this feedback. We have more clearly specified that we will collect information about potential contamination and mobility between barbershops (Section 5.6) and ensure this is part of data collection.

2. We agree that staff and participant safety should be of paramount concern. Will asking men if they self-identify as heterosexual protect participants and staff in Uganda? Perhaps instead clients could be asked questions about recent sexual behaviors with women.

   Protocol Team Response:

   We appreciate the reviewer's attention to this issue and the local context. In discussion with the HPTN Community Working Group, members felt that most men would be comfortable identifying as heterosexual, even if they also had sex with men. We will also review pre-screening and CRF questions about sexual behavior with the local Community Advisory Board before use with participants. This will help us ensure that questions are most appropriate for the context and to maintain participant and staff safety.

3. In the response on p. 9 (Primary Reviewer 3, comment 12 about confidentiality of information at the barbershop), the investigators note that the barber will keep a simple checklist of clients to indicate if they received HIV education, took HIV test kits, or HIV prevention referral information. The checklist will not include the participant's name or identifying information. Will this just be a checklist of how many times the barber provided these things, with no way to link to a specific participant? Providing HIV education to the same participant 5 times would be different than providing HIV education to 5 different participants, so there should be some way to account for that.

   Protocol Team Response:

   We acknowledge the complexity of tracking exact details of participant's receiving the intervention. We will encourage clients to bring their study card, which will include their study ID number, to the barber. The barber will be able to record this number on the checklist as a way for study staff to link to other participant data. However, if the client does not bring the card, the barber will simply record that a participant-client received services. This has been clarified within the protocol (Section 6.5).

   We will also ask all participants about the frequency of their haircut visits and if they received any HIV education, referrals, or HIVST kits from the barber (Section 5.6).

4. The enrollment procedures are still a bit unclear, which leads to questions about feasibility. What does it mean that "barbers will link directly to study staff for pre screening"? Will the pre screening occur at the barbershop and, if so, how will privacy and confidentiality be protected? And if the study team can transport the participant from the barbershop to the clinic for enrollment on the

same day, how far is the clinic from some of the barbershops? Will clients need to wait around for a staff member to get there to pick them up, and how long might that take?

Protocol Team Response:

Barbers will be able to contact study staff via phone or SMS as soon as they have identified an interested client. If the study staff are nearby the barbershop, they can meet the potential participant in person to complete the pre-screening checklist in-person in a private space (within the barbershop or a nearby private space). If the study staff is unable to come to the barbershop immediately, they will be able to administer the pre-screening checklist via the phone. Barbers will be trained to assess interest in the study at the beginning of the haircut to minimize any wait time and so study staff can plan for pre-screening and travel.

The clinic is centrally located within the island since it is co-located in the primary health facility for residents and meant to be accessible to all communities. Thus, transport time from the barbershops will take between 15 minutes – 1 hour. Participants will be notified of how long they may need to wait for transport to the study clinic for enrollment and if they are unable to wait, study staff will arrange to pick them up at a difference location, if desired, while still making every effort to complete the enrollment on the same day. Additionally, the study will have field vehicles and motorcycles (commonly used mean of transport in the area) which will be on standby to pick potential participants from the barbershops to the study site.

The processes for pre-screening, transport of participants, etc. will be reviewed by the study team in real-time to maximize efficiency and minimize inconvenience to study participants. Details of procedures will be included in the SSP Manual and SOPs, so that real-time adjustments to these processes may be made most efficiently.

5. The investigators may want to give further attention to fidelity, as fidelity information may be important to informing a future, larger trial. The response to the initial reviews notes that study staff will attend peer sessions and document messages, but section 6.5 in the protocol merely states that study staff will record the number of men in attendance at the peer groups. A checklist of services that the barber provided is a start at ensuring fidelity to status-neutral HIV education, but it seems like more could be done here as well.

Protocol Team Response:

We agree that understanding the fidelity to the intervention is important to understand.

For fidelity measures, we plan to assess adherence, facilitation strategies, quality of delivery, and participant responsiveness, as described below (background added to Section 1.5). Study staff will frequently check-in with barbers and provide support and refresher trainings as the need is identified (Section 6.2).

1) *Adherence to the intervention* (i.e. was the intervention delivered?) – barbers will complete checklists about which components of the intervention were delivered (Section 6.5). Study staff will complete checklists about the components provided by the barber during the peer group

sessions (Section 6.5), and participants will be asked about which components they received (Section 5.6). We have also added measures of intervention adherence (frequency of barber-participant interaction and proportion that include delivery of the intervention) to the primary objective endpoints (Section 8.1.1.1).

2) *Facilitation strategies* (i.e. standardizing implementation so that everyone receives the same information) – barbers will complete the same training and have access to work instructions and laminated counseling sheets in order to ensure that participants receive the intervention as planned (Sections 6.2, 6.5). Study staff will also provide ongoing support to barbers and refresher training/reorientation on the intervention as needed (Section 6.2).
3) *Quality of delivery* (i.e. is the intervention delivered well to achieve intended effect) –Study staff will attend the peer group sessions to observe the intervention barbers while they deliver the HIV education to clients and record their observations on a standardized form to understand what components were delivered and the quality of messaging (Section 6.5)
4) *Participant responsiveness* (i.e. are the participants actually interested in receiving the intervention and is it acceptable?) – participants will be asked about the acceptability of the intervention and their experience in receiving the intervention in study questionnaires and in-depth interviews (Sections 5.6, 5.9).
